# The *Oplopanax elatus* genome reveals dammaradienol synthase evolution enabling reconstruction of RK type ginsenosides biosynthesis

**DOI:** 10.1038/s41467-026-74686-6

**Published:** 2026-06-24

**Authors:** Xin Wang, He Zhang, Mengying Kang, Zeyu An, Yuxin Song, Dongran Zheng, Wanjie Xue, Chang Yang, Yangxin Wang, Jikang Zhang, Shuping Zhou, Zhuo Zhang, Chang Li, Hao Wu, Wei Song, Peng Zhang, Hailong Shen, Zhong-Jian Liu, Saneyuki Kawabata, Zhichao Xu, Xin Hua, Yuhua Li, Yang Zhang, Yu Wang

**Affiliations:** 1 State Key Laboratory of Utilization of Woody Oil Resource, College of Life Science, Northeast Forestry University, Harbin, China; 2 Key Laboratory of National Forestry and Grassland Administration on Chinese Herbal Medicine, College of Life Science, Northeast Forestry University, Harbin, China; 3 School of Forestry, Northeast Forestry University, Harbin, China; 4 Department of Medicinal Chemistry and Natural Medicine Chemistry, College of Pharmacy, Harbin Medical University, Harbin, China; 5 Key Laboratory of National Forestry and Grassland Administration for Orchid Conservation and Utilization, Fujian Agriculture and Forestry University, Fuzhou, China; 6 Engineering Technology Research Center of National Forestry and Grassland Administration for Orchid Conservation and Utilization, Guangzhou Institute of Forestry and Landscape Architecture, Guangzhou, China; 7 Institute for Sustainable Agroecosystem Services, Graduate School of Agricultural and Life Sciences, The University of Tokyo, Midoricho, Nishitokyo, Tokyo Japan

**Keywords:** Secondary metabolism, Plant evolution, Genome, Natural product synthesis

## Abstract

RK-type ginsenosides are a subgroup of dehydrated dammarane saponins with valuable bioactivities, but they are not naturally produced in cultivated ginseng. The ginseng relative *Oplopanax elatus* accumulates dammaradienol, the precursor scaffold of RK-type ginsenosides, but does not produce RK-type ginsenosides. Here, we identify OeOSC14 as a specialized dammaradienol synthase that evolved through duplication and neofunctionalization of an ancestral multifunctional triterpene synthase by combining chromosome-level genome assembly, comparative genomics and biochemical analyses. Consistent with this evolutionary transition, a single N260Y substitution converts OeOSC14 from a dammaradienol-specific enzyme back into a multifunctional triterpene synthase. We further show that loss of functional C12 hydroxylase blocks the downstream oxidative step required for RK-type ginsenoside biosynthesis in *O. elatus*. Reconstitution of the pathway in *Nicotiana benthamiana* enables de novo production of ginsenosides Rk1, Rk2, and Rk3, providing evolutionary insight into triterpene diversification and establishing a plant-based route for producing these compounds.

## Abstract (web summary)

RK type ginsenosides are rare saponins not naturally produced in plants. Here, the authors use genome assembly, comparative analysis, biochemical characterization and pathway reconstruction to show that *Oplopanax elatus* evolved a specialized dammaradienol synthase, enabling RK saponins de novo production in *Nicotiana benthamiana*.

## Introduction

Plant-derived triterpenoid saponins are a major class of specialized metabolites composed of a C30 scaffold decorated with glycosyl modifications, exhibiting remarkable structural diversity, with more than 20,000 structures reported from plants^1^. Beyond their ecological roles in protecting against pests and pathogens, shaping the root microbiome, and influencing crop quality, they also contribute to pharmacological activities in humans (e.g., immunomodulatory, anti-inflammatory, and anticancer-related activities)^2^. Accordingly, triterpenoid saponins are widely valued in medicinal plants such as *Panax* (ginsenosides)^3^, *Glycyrrhiza* (glycyrrhizin)^4^, *Quillaja* (QS saponins)^5,6^ and many others, making them prominent targets for metabolic engineering and synthetic biology.

**Fig. 1:**
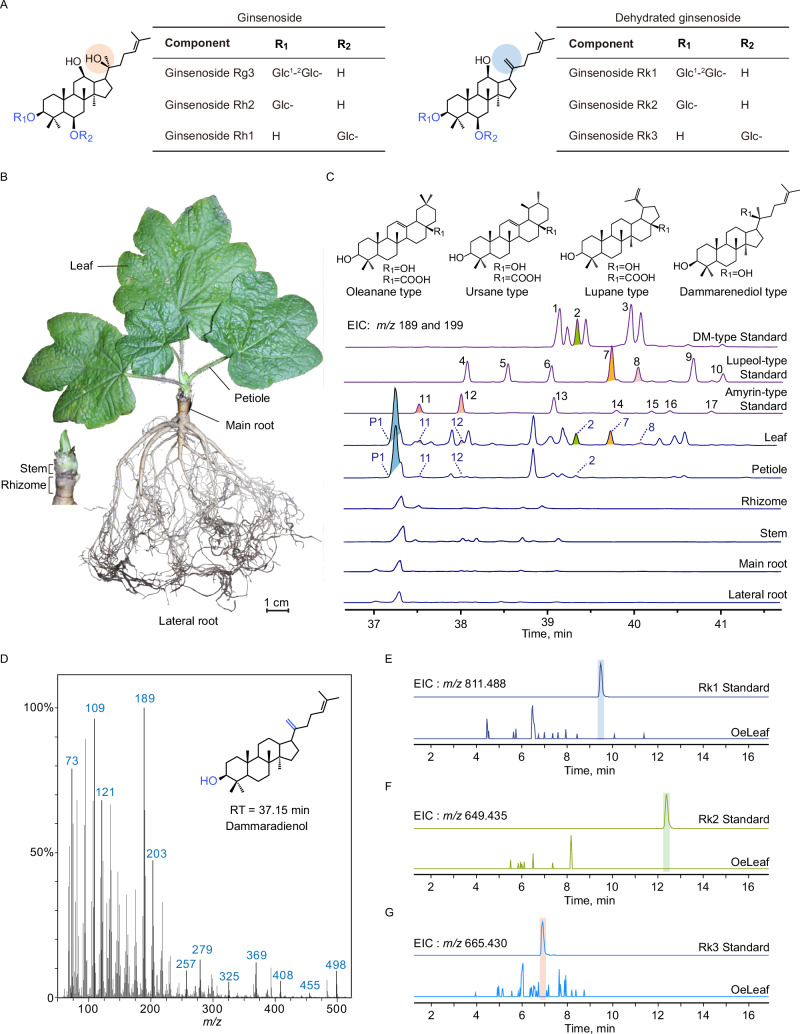
Structure and tissue distribution of triterpenoid saponins in *O. elatus.* **A** Structures of dammarane-type rare ginsenosides in comparison to their thermally dehydrated derivatives, known as RK-type ginsenosides. R_1_ and R_2_ denote the substituents at C3 and C6 of the skeleton, respectively. Glc stands for glucose. **B** Overview of a whole *O. elatus* plant, displaying tissues such as the leaf, petiole, stem, rhizome, main root, and lateral root. **C** Extracted ion chromatograms (*m*/*z* 189 and 199) from GC–MS analysis of extracts from different tissues of *O. elatus*, including leaf, petiole, stem, rhizome, main root, and lateral root. DM-type standard: 1, protopanaxadiol; 2, dammarenediol II; 3, protopanaxatriol. Lupeol-type standard: 4, lupeol; 5, *α*-23-hydroxylupeol; 6, *β*-23-hydroxylupeol; 7, betulin; 8, betulinic acid; 9, 23-hydroxybetulin; 10, 23-hydroxybetulinic acid. Amyrin-type standard: 11, *β*-amyrin; 12, *α*-amyrin; 13, erythrodiol; 14, oleanolic acid; 15, echinocystic acid; 16, ursolic acid; 17, hederagenin. Metabolite groups in *O. elatus* are highlighted in different colors. **D** Mass spectrum (EI-GC–MS) and structure of dammaradienol purified from *O. elatus*. **E–G** LC–MS extracted-ion chromatograms of *O. elatus* leaf extracts for ginsenosides Rk1 (*m*/*z* 811.488), Rk2 (*m*/*z* 649.435), and Rk3 (*m*/*z* 665.430); stacked chromatograms are shown with each trace normalized to 100% relative intensity on the y-axis.

Ginseng (*Panax ginseng*) contains hundreds of structurally distinct ginsenoside monomers, predominantly built on a dammarane backbone with diverse glycosylation at C-3 and C-6 and/or C-20^7^. RK-type ginsenosides (Rk1, Rk2, and Rk3) belong to the family of dehydrated ginsenosides, characterized by dehydration on the dammarane side chain that introduces a C-20/21 or C-20/22 double bond and increases hydrophobicity relative to their parent compounds^8^. Unlike the major ginsenosides that dominate fresh *Panax* tissues, RK-type ginsenosides are essentially absent from cultivated ginseng and are generally obtained during thermal or acid-associated processing (e.g., steaming for red ginseng), in which stepwise deglycosylation and dehydration remodel the ginsenoside profile toward less-polar products^8,9^. Mechanistically, RK-type ginsenosides (Rk1–Rk3) share a dammaradienol-type aglycone that is typically obtained by C-20/21 dehydration conversion of dammarenediol II–derived ginsenosides (e.g., Rg3 → Rk1, Rh2 → Rk2, and Rh1 → Rk3)^10^ (Fig. 1A). Importantly, RK-type ginsenosides exhibit notable anti-tumor and anti-inflammatory/immune-modulatory activities^11–13^. For example, Rk1 inhibits lung adenocarcinoma growth by inducing apoptosis and suppressing PD-L1 expression through blockade of NF-κB signaling^14^; Rk2 alleviates alcoholic liver disease by inhibiting hepatic NLRP3 inflammasome signaling while enhancing intestinal NLRP6 activity to improve barrier function, whereas Rk3 suppresses tumorigenesis through gut microbiota–immune regulation and can directly target PI3K/AKT to induce autophagy and apoptosis in hepatocellular carcinoma^15,16^. However, current access to RK-type ginsenosides still relies largely on low-efficiency conversion with substantial byproducts, which severely constrains their supply^17^.

The ginsenoside biosynthetic pathway primarily proceeds through the mevalonate (MVA) pathway to generate precursors, producing 2,3-oxidosqualene and then undergoing (i) cyclization of 2,3-oxidosqualene by oxidosqualene cyclases (OSCs) to establish the core scaffold, (ii) oxidative tailoring by cytochrome P450 monooxygenases (CYPs), and (iii) glycosylation by UDP-dependent glycosyltransferases (UGTs), thereby generating extensive structural diversity^18^. For example, dammarane-type ginsenosides are built on dammarenediol II produced by dammarenediol II synthase (DDS), followed by C-12 and C-6 hydroxylation catalyzed by PgCYP716A47 and PgCYP716A53v2, respectively, and subsequent glycosylation at C-3, C-6, and C-20 by distinct UGTs^19^. However, whether plants can synthesize RK-type ginsenosides de novo remains unclear.

From an evolutionary perspective, plant triterpene synthases originated from cycloartenol synthase (CAS), and repeated duplication events—particularly tandem duplication—enabled neofunctionalization toward specialized triterpenes (e.g., lupeol, *α*/*β*-amyrin, friedelin, and dammarane-like scaffolds)^20–22^. Such new products can arise from small sequence changes that alter carbocation rearrangements during cyclization, and multifunctional triterpene synthases (mTTSs) may further specialize over long-term evolution to predominantly produce one major triterpene scaffold^23^. For instance, the *Panax*-specific enzyme DDS likely evolved from a *β*-amyrin synthase (BAS)–like OSC ancestor via gene duplication and neofunctionalization, a process that was driven or accelerated by genome duplication events in Araliaceae^20,24^. Recently, an OSC (AarOSC20433) from *Artemisia argyi* (Asteraceae) was identified as a dammaradienol synthase (DDES) that catalyzes the conversion of 2,3-oxidosqualene into dammara-20,24-dien-3*β*-ol (dammaradienol)^25^. However, the evolutionary origin of dammaradienol synthase and the key molecular determinants underlying this product specificity remain unknown.

*Oplopanax elatus* (Araliaceae) is a close relative of *Panax* and an important medicinal plant in Traditional Chinese Medicine^26,27^. Previous phytochemical studies have shown that the triterpenoid saponins identified in *O. elatus* are predominantly of the oleanane and lupane types, including representative compounds such as cirenshenosides S, T, U, and V^28^. In a related species, *O. horridus*, the dammarane derivative dammara-20,24-dien-3*β*-acetate has also been reported^29^. These observations suggest the presence of dammaradienol-related metabolism in *Oplopanax*. However, the genetic and enzymatic foundations of this metabolic capability, as well as its connection to RK-type ginsenoside biosynthesis, have yet to be elucidated.

In this work, we show that *O. elatus* leaves accumulate dammaradienol but do not produce RK-type ginsenosides. We assemble a chromosome-level genome of *O. elatus* and identify OeOSC14 as a specialized dammaradienol synthase that evolved from a BAS-like ancestor through gene duplication, neofunctionalization and functional specialization. We further show that N260 is a key residue controlling dammaradienol synthase specificity, and that loss of the functional C12 hydroxylase blocks downstream RK-type ginsenoside biosynthesis in *O. elatus*. Finally, we reconstruct the pathway in *Nicotiana benthamiana* and achieve de novo biosynthesis of ginsenosides Rk1, Rk2, and Rk3. Together, these findings link OSC functional evolution with downstream pathway disruption to explain the absence of natural RK-type ginsenoside biosynthesis, while establishing a technical platform for the engineered heterologous production of RK-type ginsenosides.

## Results and discussion

### ***O. elatus*leaves accumulate dammaradienol, but not RK-type ginsenosides**

*O. elatus* is known to be rich in triterpenoid saponins, and previous study on root and rhizome extracts has reported predominantly oleanane- and lupane-type triterpenoids^30^. To examine the diversity and tissue-specific distribution of triterpenoid skeletons in *O. elatus*, we performed gas chromatography–mass spectrometry (GC–MS)-based metabolite profiling of extracts from root, stem, rhizome, leaf, and petiole (Fig. 1B). By comparing retention times and mass spectral fragmentation patterns with authentic triterpene standards, we detected several compounds, including betulin, betulinic acid, *β*-amyrin, *α*-amyrin and dammarenediol II (Fig. 1C; and Supplementary Fig. 1). Notably, the total ion chromatograms (TICs) and extracted ion chromatograms (EICs) at *m*/*z* 189 of leaf extracts revealed a prominent, unknown product peak (P1) at 37.15 min that showed the highest intensity (Fig. 1C; Supplementary Fig. 2). The mass spectral characteristics of this compound were distinct from those of *α*-amyrin, *β*-amyrin, or lupeol, precluding structural identification (Fig. 1D); therefore, we performed large-scale extraction, isolation and purification of P1 from leaves and determined its structure by nuclear magnetic resonance (NMR) spectroscopy, identifying it as dammara-20,24-dien-3*β*-ol (dammaradienol) (Supplementary Figs. 3–8; Supplementary Data 1). Next, to evaluate whether RK-type ginsenosides accumulate in *O. elatus*, we performed targeted LC–MS analysis using commercially available standards of ginsenosides Rk1, Rk2, and Rk3. None of these three ginsenosides were detected in leaves or any other examined tissues of *O. elatus* (Fig. 1E-G and Supplementary Fig. 9). Together, these findings indicate that *O. elatus* accumulates the RK-precursor scaffold dammaradienol in leaves, yet does not detectably produce RK-type ginsenosides (Rk1–Rk3) across the different tissues.

### Genome sequencing, assembly, and annotation of O. elatus

**Fig. 2:**
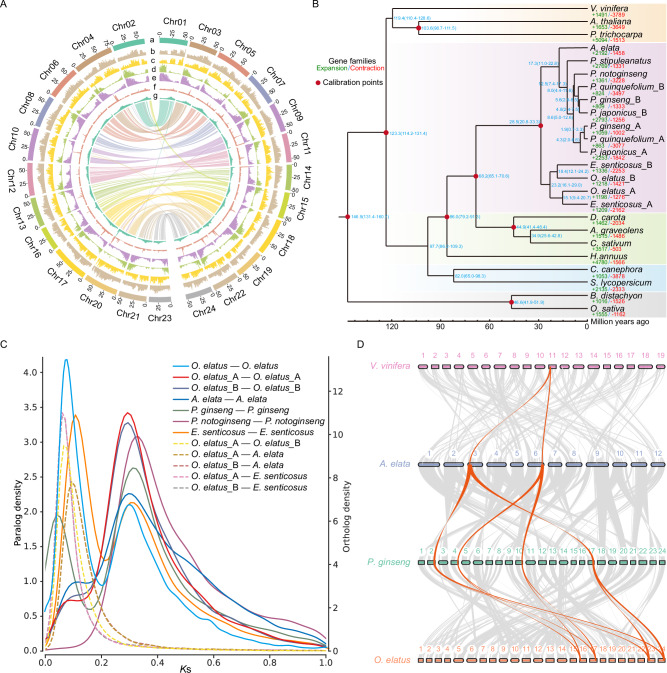
Evolutionary analysis of the *O. elatus* genome. **A** Features of the chromosome-level *O. elatus* genome. a, the outer colored bands represent scale bars of 24 chromosomes (megabases); b, distribution of Gypsy LTR retrotransposons is shown in a 100-kb window; c, distribution of Copia LTR retrotransposons is shown in a 100-kb window; d, distribution of DNA transposons; e, distribution of genes on chromosomes; f, sequencing depth; g, GC content and synteny blocks of paralogous sequences within *O. elatus* genome. **B** An inferred phylogenetic tree based on orthologs of single gene families among 19 selected species. Divergence times were estimated by MCMCTree, and the 95% confidence intervals of divergence times are shown on the internal nodes. The numbers of gene family expansions are indicated in green, and gene family contractions are indicated in red. **C** The synonymous substitutions per synonymous site (*K*s) distributions of orthologous and paralogous genes among *O. elatus*, *P. ginseng*, *P. notoginseng*, *A. elata*, and *E. senticosus*. **D**, Macrosynteny plots among *V. vinifera*, *P. ginseng, A. elata*, and *O. elatus* genomes show a 1:2:4:4 syntenic relationship.

To explore the genomic basis of dammaradienol biosynthesis, we generated a chromosome-level genome assembly of allotetraploid *O. elatus* (2n = 4x = 48). The haplotype genome size of *O. elatus* was estimated to be 762 Mb, with a high heterozygosity rate of 8.5% (Supplementary Fig. 10). In total, 45.76 Gb ( ~ 30×) PacBio HiFi reads and 126.91 Gb ( ~ 71×) Hi-C reads were generated and used for assembly (Supplementary Tables 1–2). The de novo assembly comprised 964 contigs with an N50 length of 7.5 Mb and a total assembly size of 1.49 Gb (Supplementary Table 3). Through chromosomal anchoring, the final assembly comprised two haplotypes anchored onto 24 pseudochromosomes, totaling 1.45 Gb (accounting for ~97.4% of the genome), with a contig N50 of 7.5 Mb and high BUSCO completeness of 99.38% (Fig. 2A; and Supplementary Figs. 11, 12; Supplementary Tables 3–5). Genome annotation identified repetitive sequences covering 63.68% of the assembly and predicted 61,842 high-confidence protein-coding genes, 92.01% of which were functionally annotated (Supplementary Tables 6-9).

### **Phylogenetic analysis and whole-genome duplication events in*O. elatus***

To resolve the evolutionary history of Araliaceae and the phylogenetic position of *O. elatus*, we analyzed 19 representative plant genomes, including eight Araliaceae species. A maximum-likelihood tree was constructed using 145 single-copy genes resolved Araliaceae into two strongly supported clades: one comprising *Eleutherococcus senticosus* and *O. elatus*, and the other containing *Aralia. elata* together with five *Panax* species. This topology indicates that *O. elatus* is more closely related to *E. senticosus* than to *A. elata*^31^ or *Panax*^32,33^. Notably, subgenomes A and B of *E. senticosus* formed two separate branches that clustered with subgenomes A and B of *O. elatus*, respectively, consistent with a shared allopolyploid origin involving two divergent ancestral lineages. A similar two-subgenome pattern was observed in allotetraploid *Panax* species, and the B subgenomes showed higher similarity to diploid Araliaceae genomes (e.g., *Panax notoginseng* and *A. elata*), suggesting that the B subgenome likely derives from a diploid Araliaceae-like progenitor (Fig. 2B).

Divergence-time estimation with MCMCTREE based on the same 116 orthogroups (with fossil-based calibrations from TimeTree) dated the split between Araliaceae and Apiaceae to ~68.2 Mya and the subsequent separation of the two major Araliaceae lineages to ~28.5 Mya; importantly, subgenomes A and B of *O. elatus* diverged ~23.2 Mya, predating the species divergences within the *A. elata*–*Panax* clade (Fig. 2B). We then compared the genomes of candidate plant species to obtain gene families that are significantly expanded in *O. elatus* or unique to *O. elatus*. GO and KEGG enrichment analyses showed that expanded families were overrepresented in triterpenoid metabolic processes (GO) and sesquiterpenoid and triterpenoid biosynthesis (KEGG), consistent with the rich triterpenoid chemistry of this species (Supplementary Figs. 13–14).

Whole-genome duplication (WGD) is a major driver of gene-family diversification and metabolic innovation in plants^34,35^. We therefore analyzed synonymous substitution rate (*K*s) distributions for paralogous gene pairs in *O. elatus*, *E. senticosus*, *P. ginseng*, *P. notoginseng*, and *A. elata*. These analyses revealed two prominent peaks at ~0.3 and ~0.1 (Fig. 2C). Among Araliaceae species, the shared peak at *K*s ≈ 0.3 is indicative of a common WGD event (Pg-*β*)^33,36^. This inference was further supported by the 2:1 syntenic relationship between diploid Araliaceae genomes (e.g., *P. notoginseng* and *A. elata*) and *Vitis vinifera*^37^ (Supplementary Fig. 15). In addition, allotetraploid species, including *O. elatus*, *E. senticosus*, and *P. ginseng*, exhibited an additional *K*s peak near ~0.1 that was absent from diploid species. Orthologous gene pairs between the two subgenomes within each allotetraploid also showed a peak at ~0.1. Consistent with this pattern, synteny analysis revealed a 1:2:4:4 relationship among *V. vinifera*, *A. elata*, *P. ginseng*, and *O. elatus*, supporting a relatively recent allopolyploidization event in Araliaceae that occurred after the shared Araliaceae WGD (Fig. 2D; and Supplementary Fig. 16).

### **Identification of functional dammaradienol synthase in*O. elatus***

**Fig. 3:**
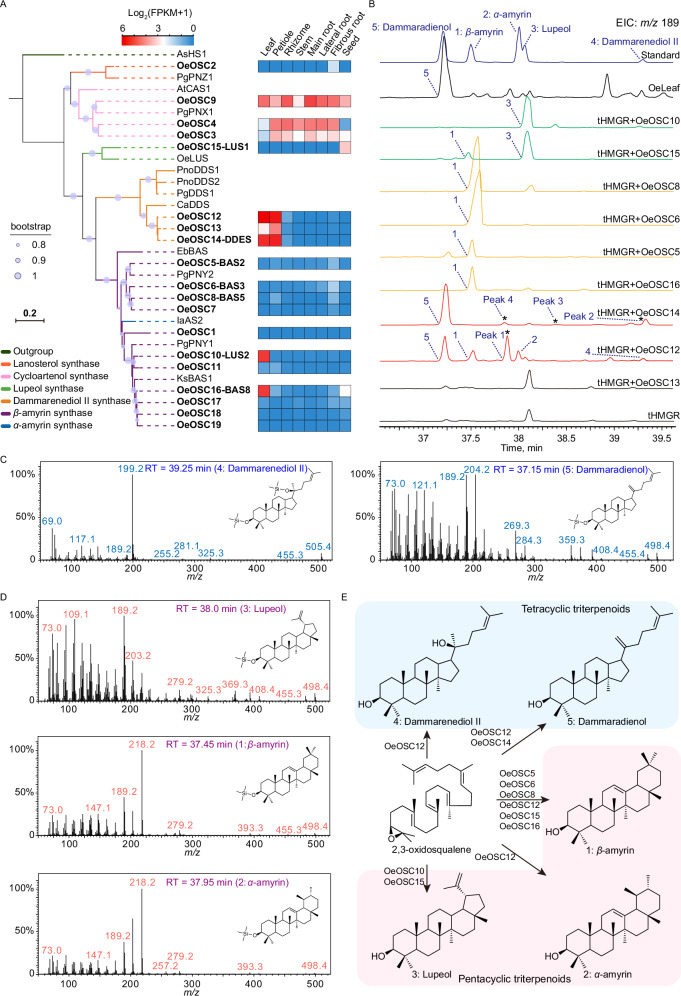
Gene function validation of *O. elatus* oxidosqualene cyclases (OeOSCs). **A** Maximum-likelihood phylogeny of OSCs identified in *O. elatus*, inferred with FastTree using the JTT model. Filled purple circles denote nodes with bootstrap support >0.9; the scale bar indicates substitutions per site. OSC subfamilies are color-coded, and *O. elatus* OSCs are shown in bold. A heatmap shows expression profiles of *OSC* genes across *O. elatus* tissues (leaf, petiole, rhizome, stem, seed, main root, lateral root, and fibrous root). **B** GC–MS extracted-ion chromatograms (*m*/*z* 189) of leaf extracts from *N. benthamiana* transiently co-expressing *tHMGR* with individual *OeOSC* genes. Peaks marked with asterisks indicate products of unknown structure. **C** Mass spectrometry fragmentation profiles of OeOSC-derived tetracyclic triterpene products. **D** Mass spectrometry fragmentation profiles of OeOSC-derived pentacyclic triterpene products. **E** Enzymatic cyclization of 2,3-oxidosqualene to *β*-amyrin, *α-*amyrin, lupeol, dammaradienol, and dammarenediol II by OeOSCs. Source data are provided as a Source Data file.

Given the accumulation of dammaradienol in *O. elatus* leaves, we hypothesized that the genome of *O. elatus* encodes an oxidosqualene cyclase (OSC) that specifically catalyzes the cyclization of 2,3-oxidosqualene to form dammaradienol. To test this hypothesis, we performed a BLAST search of the *O. elatus* genome using known ginseng OSC sequences as queries. After excluding incomplete proteins shorter than 600 amino acids, we identified 19 full-length putative *OSC* genes (*OeOSC1*–*OeOSC19*; Supplementary Data 2) encoding proteins of 751–776 amino acids. Phylogenetic analysis of the 19 OeOSC protein sequences using a maximum-likelihood approach grouped them into five subfamilies (Fig. 3A). Among them, OeOSC2, OeOSC3, OeOSC4, and OeOSC9 clustered with known cycloartenol synthase (CAS) and lanosterol synthase (LAS) genes, suggesting roles in sterol biosynthesis. The remaining 15 *OeOSC* genes are grouped with lupeol synthase (LUS), *β*-amyrin synthase (BAS), and dammarane-type OSC clades, which are typically associated with pentacyclic and tetracyclic triterpene scaffolds formation.

Transcriptomic profiling across tissues further showed that these 15 triterpene-related *OeOSC* genes exhibited distinct expression patterns. After excluding six lowly expressed genes (*OeOSC1*, *OeOSC7*, *OeOSC11*, *OeOSC17*, *OeOSC18*, and *OeOSC19*; FPKM < 1), we selected nine candidates for functional characterization by transient expression in *N. benthamiana* leaves. Of these, five genes (*OeOSC10*, *OeOSC12*, *OeOSC13*, *OeOSC14*, and *OeOSC16*) were predominantly expressed in leaves, consistent with the tissue distribution of triterpenoid scaffolds, whereas the other four candidates were mainly expressed in roots and seeds (Supplementary Fig. 17). To enhance metabolic flux toward 2,3-oxidosqualene, we co-expressed each candidate with a truncated oat 3-hydroxy-3-methylglutaryl coenzyme A reductase lacking the N-terminal transmembrane domain (AstHMGR)^38^. GC–MS analysis of infiltrated leaves revealed that seven candidates produced detectable triterpene products from 2,3-oxidosqualene, whereas OeOSC13 showed no detectable activity (Fig. 3B). Comparison of retention times and mass spectra with authentic standards identified OeOSC10 as a lupeol synthase (Supplementary Fig. 18); OeOSC5, OeOSC6, OeOSC8, and OeOSC16 as *β*-amyrin synthases; and OeOSC15 as a multifunctional enzyme producing both lupeol and *β*-amyrin.

Compared with the control lacking a heterologous OSC, transient expression of OeOSC12 produced four additional peaks at 37.15, 37.45, 37.95, and 39.25 min, corresponding to dammaradienol, *β*-amyrin, *α*-amyrin, and dammarenediol II, respectively. In addition, OeOSC12 generated a prominent peak at 37.85 min that did not match any available standard, indicating the formation of an uncharacterized triterpene product. By contrast, OeOSC14 produced a major new peak at 37.15 min whose retention time and mass spectrum matched those of dammaradienol, identifying OeOSC14 as a functional dammaradienol synthase (Fig. 3B–E). We therefore designated OeOSC14 as dammaradienol synthase (DDES). In addition to this major product, OeOSC14 also generated several minor unknown peaks at 37.80, 38.35, and 39.30 min, suggesting that although it predominantly produces dammaradienol, it also forms low-abundance byproducts (Supplementary Fig. 18).

### Origin and evolution of dammaradienol synthase

**Fig. 4:**
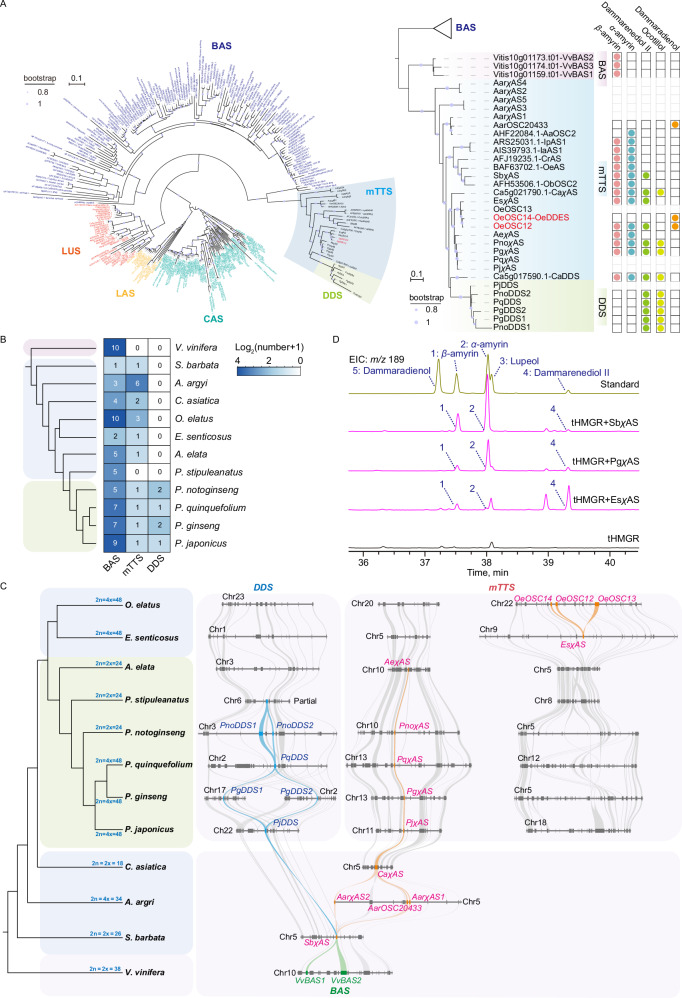
Evolution and diversification of dammaradienol synthase inferred from comparative genomics. **A** A maximum-likelihood phylogeny of functionally characterized eudicot OSCs together with all predicted OSCs from *V. vinifera*, *S. barbata, A. argyi, C. asiatica,* and eight Araliaceae species. Filled purple circles denote nodes with bootstrap support >0.8; the scale bar indicates substitutions per site. OSC subfamilies are color-coded, and enlarged views of BAS, mTTSs, and DDS subfamilies are shown at right with their products. **B** Copy-number distribution of OSC subfamilies across *V. vinifera*, *S. barbata, A. argyi, C. asiatica,* and eight Araliaceae species. The species phylogeny is shown on the left; detailed phylogenetic results are provided in Supplementary Fig. 21. **C** Synteny analysis of *OSC* loci across *V. vinifera*, *S. barbata*, *A. argyi*, *C. asiatica*, and eight Araliaceae species. The left panel shows the species phylogeny, and the right panel displays microsynteny within the corresponding syntenic blocks. Colored rectangles indicate *OSC* genes connected by synteny, whereas gray rectangles indicate flanking genes; line thickness reflects sequence identity. *OSC* genes and links are colored by subfamily (BAS, green; mTTS, orange; DDS, blue). **D** GC–MS extracted-ion chromatograms (*m*/*z* 189) of leaf extracts from *N. benthamiana* transiently co-expressing tHMGR and representative mTTSs from *P. ginseng*, *E. senticosus*, and *S. barbata*. Standard: 1, *β*-amyrin; 2*, α*-amyrin; 3, lupeol; 4, dammarenediol II. Source data are provided as a Source Data file.

To trace the evolutionary origin of dammaradienol synthase, we performed a phylogenetic analysis that integrated 129 functionally characterized eudicot oxidosqualene cyclases (OSCs) with all OSCs identified from eight Araliaceae genomes (*O. elatus*, *E. senticosus*, *A. elata*^31^, *Panax stipuleanatus*^33^, *P. notoginseng*^32^, *Panax quinquefolium*^33^, *P. ginseng*^33^, and *Panax japonicus*^33^), together with OSCs from *Scutellaria barbata*^39^, *A. argyi*^40^, *Centella asiatica*^41^, and *V. vinifera*^37^ (Fig. 4A; and Supplementary Fig. 19). Based on their relationships to characterized enzymes, the sequences were assigned to putative functional groups, including cycloartenol synthase (CAS), lanosterol synthase (LAS), lupeol synthase (LUS), *β*-amyrin synthase (BAS), and other multifunctional triterpene synthases (mTTSs). Notably, the functionally validated OeOSC14 from *O. elatus* and *Panax* dammarenediol synthases (DDSs) were nested within the BAS/mTTS lineage, indicating that these tetracyclic triterpene synthases likely originated from a BAS-like ancestor.

The BAS-like group contained canonical BASs that predominantly produce *β*-amyrin, occasionally together with minor pentacyclic byproducts such as *α*-amyrin. The mTTS-like group, which includes OeOSC14, comprised multifunctional OSCs that generate *α*- and/or *β*-amyrin as major products while producing trace amounts of additional triterpenes, including dammaradienol and dammarenediol II. By contrast, the DDS-like group contained the *Panax* DDSs, which primarily produce dammarenediol II with only low-abundance byproducts such as ocotillol-type triterpenes^20^. We next examined the lineage distribution of BAS-like, mTTS-like, and DDS-like OSCs across representative species (Fig. 4B). BAS-like OSCs were broadly distributed across all examined taxa, whereas mTTS-like OSCs were detected in multiple Araliaceae species as well as several non-Araliaceae eudicots, although with variable copy numbers. By contrast, DDS-like OSCs were restricted to a subset of *Panax* species and were not detected in *O. elatus*, *E. senticosus*, or *A. elata*.

To further resolve the genomic relationships among these OSC types, we carried out comparative synteny analyses across representative genomes (Fig. 4C; Supplementary Figs. 20 and 21). The BAS-containing region in *V. vinifera* showed clear microsyntenic relationships with OSC-containing regions in *S. barbata*, *A. argyi*, *C. asiatica*, and Araliaceae, supporting an origin of mTTSs from ancestral *BAS* genes followed by lineage-specific neofunctionalization^20,24^. Within this framework, two related OSC-associated branches were apparent in Araliaceae. One branch contained the DDS-associated loci, which were well conserved across *Panax* species. In the corresponding syntenic interval, however, *A. elata* retained only a partial DDS-related sequence, whereas no intact DDS ortholog was detected in *O. elatus*^31^. The second branch contained mTTS-associated loci, including *AeχAS*, *Panax χAS* genes, *EsχAS*, and the *O. elatus* region harboring *OeOSC12*, *OeOSC13*, and *OeOSC14*. These observations suggest that the two OSC-associated branches were retained differentially after the shared Araliaceae genome duplication, with DDS-like copies preserved in *Panax* but pseudogenized or lost in *A. elata* and *O. elatus*^31^.

In *O. elatus*, *OeOSC12*, *OeOSC13*, and *OeOSC14* are located within a locally expanded mTTS-associated region on Chr22 that is syntenic with the *EsχAS* locus but not conserved as a comparable local expansion in *Panax* (Fig. 4C). This pattern suggests that small-scale tandem duplication and local genome rearrangement contributed to the expansion of mTTS-like OSCs in the *Oplopanax/Eleutherococcus* lineage after its divergence from *Panax*. Functional assays further supported the distinct catalytic properties of this lineage: uncharacterized mTTS-like OSCs from *P. ginseng*, *E. senticosus*, and *S. barbata*, when transiently expressed in *N. benthamiana* together with a truncated HMGR to enhance precursor supply, all exhibited multifunctional triterpene synthase activity and produced mixtures of *α*-amyrin, *β*-amyrin, and dammarenediol II, albeit with species-specific product ratios (Fig. 4D). Taken together, these results support a model in which *OeOSC14* emerged from a BAS-like ancestor via neofunctionalization within the mTTS-like lineage and was further shaped by local duplication and functional specialization in *O. elatus*, ultimately yielding an enzyme that preferentially channels 2,3-oxidosqualene toward dammaradienol production.

### N260 drives dammaradienol synthase specificity

**Fig. 5:**
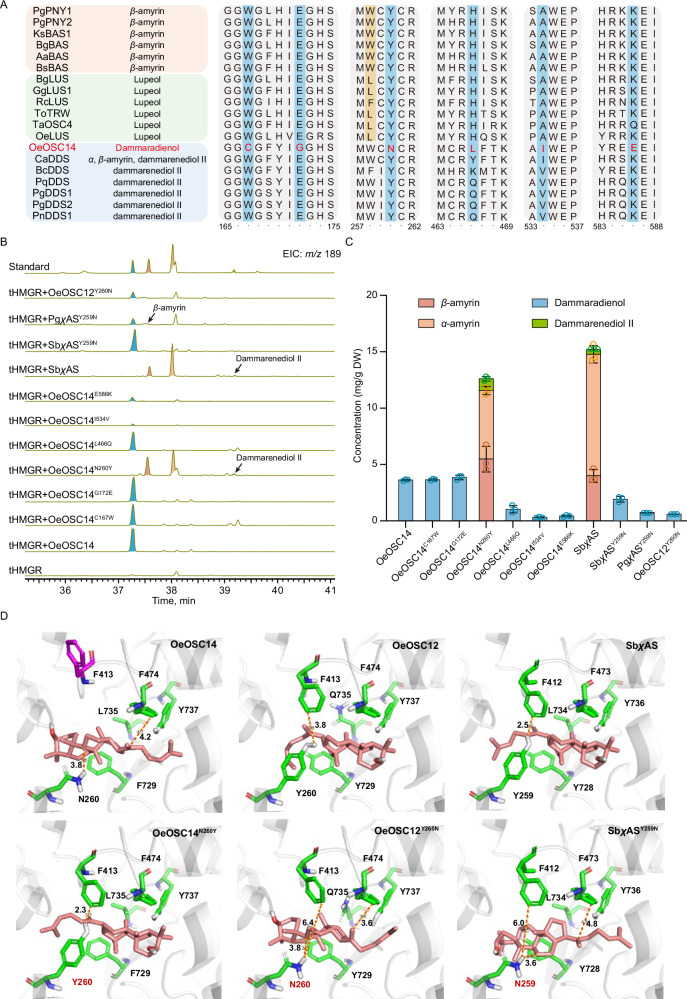
Residues underlying product specificity of OeOSC14. **A** Multiple sequence alignment of OeOSC14 and representative OSCs, highlighting active-site residues (blue) and positions selected for mutagenesis (red). **B** GC–MS extracted-ion chromatograms (*m*/*z* 189) of leaf extracts from *N. benthamiana* transiently co-expressing tHMGR with OeOSCs or their mutants. Peaks corresponding to dammaradienol (blue), *β*-amyrin (orange), *α*-amyrin (yellow), and dammarenediol II (green) are indicated. **C** Quantification of triterpenes produced by OeOSC14, Sb*χ*AS and their variants as well as Pg*χ*AS^Y259N^, OeOSC12^Y260N^. Bars show mean ± s.e.m. (*n* = 3 biological replicates). Absolute yields were calculated using standard curves generated from purified standards; **D** Molecular docking models of dammaradienol complexes with OeOSC14, OeOSC12, Sb*χ*AS, and their respective variants (OeOSC14^N260Y^, OeOSC12^Y260N^, and Sb*χ*AS^Y259N^), showing the spatial interactions within the mTTSs active pocket. Dammaradienol is depicted as pink sticks. Green and magenta labels indicate amino acid residues located within and beyond 5 Å of the ligand, respectively. Interatomic distances are marked by red dashed lines with numerical values. Source data are provided as a Source Data file.

To identify the key amino acid residues underlying the functional shift from multifunctional triterpene synthases (mTTSs) to dammaradienol synthase (DDES), we performed a multiple sequence alignment of OeOSC14 with representative members of the DDS, BAS, and LUS families. Comparative analysis across OSC proteins revealed six candidate active-site residues that are highly conserved in other OSC subfamilies but divergent in OeOSC14, suggesting that they may contribute to catalytic specificity (Fig. 5A; and Supplementary Fig. 22).

To evaluate the functional contributions of these residues, we generated single-site mutants in the OeOSC14 background, and each candidate residue was individually replaced with the corresponding conserved amino acid found in other OSC subfamilies. The product profiles of the resulting mutants were compared with those of WT after transient expression in *N. benthamiana*. GC–MS analysis showed that, among all substitutions tested, N260Y caused the most dramatic change in product outcome. Whereas wild-type OeOSC14 produced dammaradienol as the predominant product, OeOSC14^N260Y^ lost dammaradienol synthesis and instead generated a typical mTTS-like product mixture consisting of *α*-amyrin, *β*-amyrin, and dammarenediol II (Fig. 5B, C). By contrast, substitutions at C167 and G172 had little effect on product identity or yield, as OeOSC14^C167W^ and OeOSC14^G172E^ retained dammaradienol production at levels comparable to the wild type. Mutations at L466, I534, and E586 did not alter product identity but markedly reduced dammaradienol accumulation, with OeOSC14^I534V^ and OeOSC14^E586K^ showing near-complete loss of activity and OeOSC14^L466Q^ showing a substantial but partial reduction. Together, these results identify N260 as a major determinant of DDES specificity, whereas L466, I534, and E586 appear to contribute primarily to catalytic efficiency.

Next, we performed a reciprocal mutagenesis in representative mTTS-like OSCs from *S. barbata* (Sb*χ*AS) and *P. ginseng* (Pg*χ*AS). Wild-type Sb*χ*AS produced a mixture of *α*-amyrin, *β*-amyrin, and trace dammarenediol II, consistent with its multifunctional profile. Strikingly, introduction of the reciprocal substitution in Sb*χ*AS (Y259N) redirected product formation almost completely toward dammaradienol and largely abolished the accumulation of *α*-/*β*-amyrin and dammarenediol II (Fig. 5B, C). Similarly, the Pg*χ*AS^Y259N^ mutant also acquired dammaradienol-producing activity, although at a substantially lower level than Sb*χ*AS^Y259N^. These reciprocal substitutions indicate that residue 260 acts as a key switch controlling the cyclization trajectory of OSCs and strongly influences the transition between multifunctional triterpene synthase and dammaradienol synthase activities.

It is noteworthy that OeOSC12 and OeOSC14 are highly similar and differ at only 20 amino acid positions (Supplementary Fig. 23). Unlike OeOSC14, OeOSC12 carries tyrosine at position 260 but still exhibits a multifunctional product profile that includes dammaradienol. When the reciprocal substitution was introduced into OeOSC12, the resulting OeOSC12^Y260N^ mutant shifted toward a simplified product profile and produced dammaradienol as the major detectable product (Fig. 5C). This result demonstrates that residue 260 is a major determinant of dammaradienol synthase specificity.

Nevertheless, the production of dammaradienol as a minor product by wild-type OeOSC12 indicates that residue 260 alone cannot fully account for the ability to generate this scaffold. Thus, additional amino acid differences between OeOSC12 and OeOSC14 may also contribute to shaping the catalytic trajectory that leads to dammaradienol formation. This conclusion is further supported by evidence from other OSCs: the Y407A mutation in oat AsOSC6, which contains Phe at the position corresponding to residue 260, was reported to redirect catalysis toward dammaradienol^42^, whereas the dammaradienol synthase AarOSC20433 from *A. argyi* naturally carries Leu at the corresponding position^25^ (Supplementary Fig. 24). Together, these observations suggest that residue 260 acts as a key determinant of product specificity, but that full dammaradienol-forming capacity likely requires coordinated contributions from additional residues.

To gain structural insight into this residue-dependent shift in product specificity, we next performed molecular docking analyses of OSCs and their mutants with dammaradienol using AlphaFold3-predicted structures and AutoDock. The docking results revealed that the N260Y substitution altered the orientation of dammaradienol within the catalytic pocket. In wild-type OeOSC14, the docked dammaradienol was surrounded within 5 Å by N260, F474, F729, L735, and Y737. Specifically, N260 was positioned close to the C10 atom of dammaradienol, with a distance of 3.8 Å, whereas F474 was located near the C20 atom, with a distance of 4.2 Å. These spatial relationships suggest that N260 and F474 may help stabilize a specific binding pose of dammaradienol in wild-type OeOSC14. By contrast, in the OeOSC14^N260Y^ mutant, the ligand-proximal environment was reorganized, with F413 additionally recruited into the 5 Å range of docked dammaradienol. Moreover, the introduced Y260 was positioned close to F413, with a distance of 2.3 Å. These spatial rearrangements indicate that the N260Y substitution may reshape the active-site pocket through a potential Y260–F413 interaction, thereby altering the binding pose of dammaradienol. Consistent with this hypothesis, electrostatic surface potential analysis showed an apparent change in the charge distribution and surface properties of the binding pocket in OeOSC14^N260Y^ compared with wild-type OeOSC14, further supporting the idea that N260Y induces local pocket reconfiguration associated with altered product specificity (Supplementary Fig. 25).

Moreover, a comparable structural pattern was observed for OeOSC12, Pg*χ*AS, and Sb*χ*AS. In these mTTS-like enzymes, Tyr at position 259/260 was positioned close to the conserved Phe at position 412/413, resembling the Y260–F413 spatial relationship observed in OeOSC14^N260Y^. Conversely, the Y259/260N substitutions increased this separation and shifted the modeled dammaradienol pose toward an OeOSC14-like orientation (Fig. 5D, Supplementary Figs. 26-27). This structural shift is consistent with the experimentally observed redirection toward dammaradienol production in Sb*χ*AS^Y259N^, Pg*χ*AS^Y259N^, and OeOSC12^Y260N^. Together, these results support a model in which the spatial relationship between residue 259/260 and the conserved Phe 412/413 serves as a structural indicator of the active-pocket packing state. The N260-containing configuration is associated with an OeOSC14-like ligand-binding mode and dammaradienol formation, whereas the Y260-containing configuration is associated with a pocket state that allows continued cyclization toward multifunctional or pentacyclic triterpene products.

To clarify the distinct catalytic outcomes of OeOSC14 and the N260Y variant, we carried out quantum-chemical calculations starting from the shared dammarenyl cation intermediate. In the wild-type enzyme, the reaction is predominantly terminated at the dammarenyl cation stage by deprotonation-mediated double bond formation to generate dammaradienol, consistent with an important role of N260 in promoting this quenching step. Energetically, the computed electronic energy of the baccharenyl cation (stage B) is higher than that of the dammarenyl cation (stage A), indicating that progression from A to B is intrinsically uphill. Moreover, stage B can undergo an isomerization to an alternative carbocation (stage B2), which further reduces flux toward pentacyclic scaffold formation. By contrast, OeOSC14^N260Y^ yields abundant pentacyclic triterpenes (e.g., *β*- and *α*-amyrins), consistent with the mutation enhancing the feasibility of advancing beyond stage A and/or reducing diversion through the B → B2 isomerization route, thereby favoring continued cyclization and subsequent *β*-amyrin formation (Supplementary Fig. 28). Collectively, our data identify N260 as the critical determinant underlying the evolution of OeOSC14 specificity and as a core molecular switch enabling interconversion between mTTS- and DDES-like catalytic outcomes.

### **Loss of C12 hydroxylase underlies the absence of RK-type ginsenosides in*O. elatus***

**Fig. 6:**
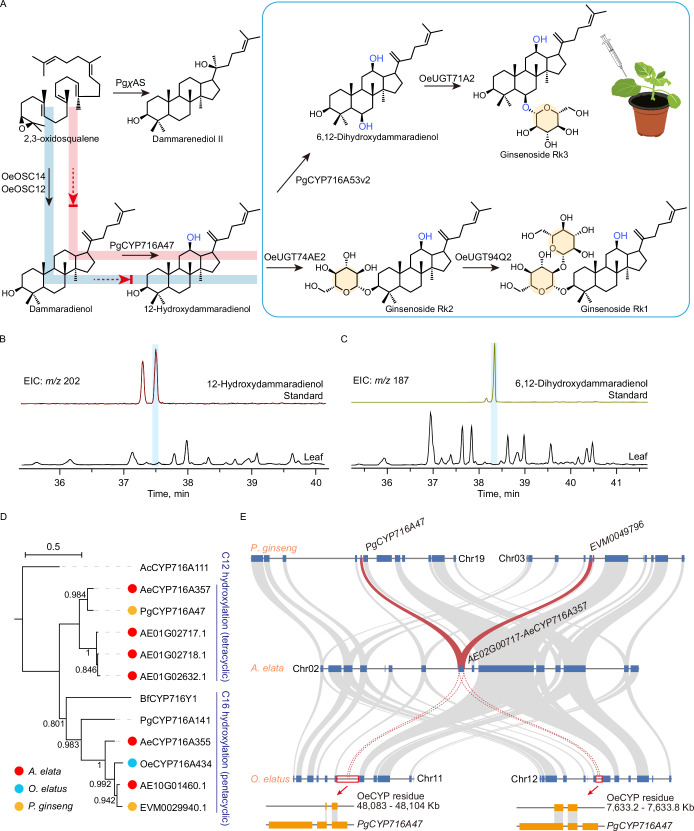
Genomic loss of the C12-hydroxylase in *O. elatus.* **A** Proposed biosynthetic route toward RK-type ginsenosides in *O. elatus* and *P. ginseng*. Red and blue lines indicate the pathways in *P. ginseng* and *O. elatus*, respectively. CYP, cytochrome P450; UGT, UDP-glycosyltransferase. **B** GC–MS extracted-ion chromatograms (*m*/*z* 202) of *O. elatus* leaf extracts. Retention-time windows matching authentic standards are highlighted in blue. **C** GC–MS extracted-ion chromatograms (*m*/*z* 187) of *O. elatus* leaf extracts. Retention-time windows matching authentic standards are highlighted in blue. **D** Phylogeny of the CYP clade containing enzymes responsible for C-12 hydroxylation in dammarane-type saponin biosynthesis and C-16 hydroxylation in oleanane-type saponin biosynthesis, rooted with AcCYP716A111. The full tree is shown in Supplementary Fig. 30. **E** Cross-species synteny of C12-hydroxylase CYP loci among *P. ginseng*, *A. elata*, and *O. elatus*. Red lines indicate syntenic relationships among *CYP* genes, and red dashed lines denote syntenic blocks lacking a complete *CYP* gene. OeCYP residue indicates the *O. elatus* ortholog of PgCYP716A47. Gene details are provided in Supplementary Figs. 33 and 34. Source data are provided as a Source Data file.

Based on the structural features of RK-type ginsenosides and established biosynthetic routes for dammarane-type ginsenosides, we hypothesize that RK-type biosynthesis initiates from dammaradienol, proceeds through sequential cytochrome P450 (CYP)–mediated hydroxylations at the C12 and C6 positions, and terminates with glycosylation mediated by UDP-glycosyltransferases (UGTs) at the C3 and C6 positions (Fig. 6A). Although dammaradienol accumulates in *O. elatus* leaves, RK-type ginsenosides are not detected, suggesting that the pathway is blocked at an early tailoring step. To test this possibility, we performed GC–MS analysis of the putative hydroxylated intermediates 12-hydroxydammaradienol and 6,12-dihydroxydammaradienol (Fig. 6B, C; Supplementary Fig. 29; and Supplementary Method 1). Neither compound was detectable in the tissue examined, supporting the conclusion that RK-type biosynthesis in *O. elatus* is arrested at the initial oxidation step, most likely C12 hydroxylation of dammaradienol.

In *P. ginseng*, the cytochrome P450 monooxygenases PgCYP716A47 (protopanaxadiol synthase, PgPPDS) and PgCYP716A53v2 (protopanaxatriol synthase, PgPPTS) catalyze C12 and C6 hydroxylation of dammarenediol II, respectively, thereby generating the dammarane sapogenins protopanaxadiol and protopanaxatriol, which are subsequently glycosylated to yield diverse ginsenosides^43,44^. Given the close phylogenetic relationship between *P. ginseng* and *O. elatus*, we reasoned that orthologous CYP716A enzymes might similarly mediate early oxidative tailoring of dammaradienol in *O. elatus*. Accordingly, we conducted a comprehensive phylogenetic analysis of *CYP* genes in the *O. elatus* genome (Supplementary Fig. 30); however, no unambiguous ortholog of *PgCYP716A47* was identified (Fig. 6D; and Supplementary Fig. 31). In addition, functional characterization showed that OeCYP716A434, which falls in a clade adjacent to PgCYP716A47 in the phylogenetic tree, catalyzes C16 hydroxylation of the pentacyclic triterpene lupeol and does not exhibit activity toward the dammarane scaffold, further indicating that *O. elatus* lacks a functional CYP716A enzyme capable of catalyzing the initial C12 hydroxylation step in RK-type biosynthesis (Supplementary Fig. 32).

Previous study showed that AeCYP716A357, the PgCYP716A47 ortholog in the closely related species *A. elata*, catalyzes the conversion of dammarenediol II to protopanaxadiol when heterologously expressed in yeast, indicating conservation of C12 hydroxylation capacity within Araliaceae^31^. Consistent with this, synteny analysis revealed that *PgCYP716A47* and *AeCYP716A357* reside in conserved genomic regions. By contrast, the two corresponding syntenic regions in *O. elatus*, representing the two subgenomes, both lacked an intact *CYP716A47*-like gene and instead retained only short truncated *CYP*-like remnants (Fig. 6E; and Supplementary Figs. 33, 34). One region preserved only an ~350 bp 3′ fragment, and the other retained an ~130 bp 3′ fragment, both far shorter than the ~1.5 kb full-length coding sequence of functional *CYP716A47* homologs. These remnants do not contain a complete open reading frame and are therefore unlikely to encode functional proteins. Thus, both *PgCYP716A47*-corresponding loci in *O. elatus* appear to have undergone pseudogenization or partial deletion.

In agreement with this genomic disruption, C12-hydroxylation–dependent dammarane-type ginsenosides (e.g., Rg3 and Rh2) and downstream derivatives (e.g., Rh1) were not detected in *O. elatus* by LC–MS, further supporting the loss of C12 hydroxylase function (Supplementary Fig. 35). Collectively, these results suggest that both *CYP716A47*-like loci have been inactivated in *O. elatus*, resulting in loss of C12 hydroxylation capacity and, consequently, the absence of RK-type ginsenoside biosynthesis.

### **Engineering RK-type ginsenoside biosynthesis in*N. benthamiana***

**Fig. 7:**
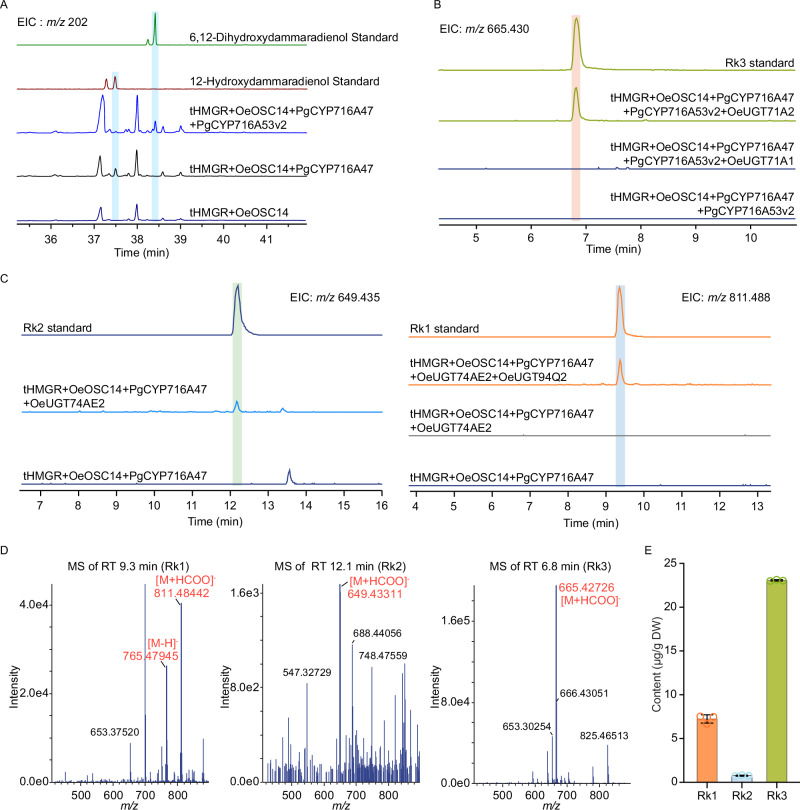
Reconstruction of the RK-type ginsenoside biosynthetic pathway in *N. benthamiana.* **A** GC–MS extracted-ion chromatograms (*m*/*z* 202) of leaf extracts from *N. benthamiana* transiently co-expressing *tHMGR*, *OeOSC14*, *PgCYP716A47*, and *PgCYP716A53v2*. Product peaks are highlighted in blue. **B** LC–MS extracted-ion chromatograms (*m*/*z* 665.430) of leaf extracts from *N. benthamiana* transiently co-expressing *tHMGR*, *OeOSC14*, *CYP*s, and *UGT*s. Product peaks of ginsenoside Rk3 are highlighted in orange. **C** LC–MS extracted-ion chromatograms (*m*/*z* 649.435 and 811.488) of leaf extracts from *N. benthamiana* transiently co-expressing *tHMGR*, *OeOSC14*, *CYP*s, and *UGT*s. Product peaks of ginsenoside Rk2 and Rk1 are highlighted in green and blue, respectively. **D**, MS fragmentation spectra of products detected in panels (**B** and **C**). **E** Yields of ginsenosides Rk1, Rk2, and Rk3 produced via heterologous biosynthesis in *N. benthamiana*. Error bars indicate the mean and standard error of three biological replicates. Source data are provided as a Source Data file.

Tobacco provides a robust heterologous chassis for triterpenoid saponin biosynthesis. Previous studies have shown that reconstituting triterpene pathways in *N. benthamiana* leaves via transient co-expression enables the production of ginsenoside Ro from *P. ginseng* and astragalosides from *Astragalus membranaceus*^45,46^. Building on this platform, we transiently co-expressed the *O. elatus OeOSC14* to supply dammaradienol, together with *PgCYP716A47* and *PgCYP716A53v2* in *N. benthamiana*. GC–MS analysis detected the expected hydroxylated intermediates, 12-hydroxydammaradienol and 6,12-dihydroxydammaradienol (Fig. 7A; and Supplementary Fig. 36).

Ginsenosides Rk1, Rk2, and Rk3 are primarily glycosylated at the C3 and C6 positions of these dammaradienol-derived sapogenins. To identify UDP-glycosyltransferases (UGTs) potentially responsible for these glycosylation steps, we used previously characterized *P. ginseng UGTs* involved in dammarane-type ginsenoside biosynthesis, including *UGTPg45*, *PgUGT94Q2*, and *UGTPg101*, as reference genes for genome-wide candidate screening^47,48^. Combining UGT phylogenetic analysis with interspecific synteny between *O. elatus* and *P. ginseng*, we identified five candidate *O. elatus UGT* genes potentially involved in RK-type ginsenoside biosynthesis (Supplementary Figs. 37-38). Transcriptome-based expression profiling further revealed that *OeUGT94Q2*, *OeUGT74AE2*, and *OeUGT71A2* share a similar expression pattern with predominant expression in roots; *OeUGT71A1* was highly expressed across all tissues, whereas *OeUGT74AE1* was barely detectable in any tissue tested (Supplementary Fig. 38). We therefore cloned the four expressed *OeUGTs* (*OeUGT94Q2*, *OeUGT74AE2*, *OeUGT71A2*, and *OeUGT71A1*) and performed transient expression in *N. benthamiana* for functional characterization.

Next, we co-expressed *AstHMGR*, *OeOSC14*, and *PgCYP716A47* with selected *OeUGTs*, with or without *PgCYP716A53v2*, to generate three enzyme sets: G-Rk3 (plus *PgCYP716A53v2* and *OeUGT71A1* or *OeUGT71A2*), G-Rk2 (plus *OeUGT74AE2*), and G-Rk1 (plus *OeUGT74AE2* and *OeUGT94Q2*). LC–MS analyses identified new peaks at 6.8 min (G-Rk3), 12.2 min (G-Rk2), and 9.4 min (G-Rk1), which matched authentic standards of ginsenosides Rk3, Rk2, and Rk1, respectively (Fig. 7B–D). Using this system, de novo biosynthesis of ginsenosides Rk3, Rk2, and Rk1 in *N. benthamiana* leaves was achieved, yielding 23.07, 0.76, and 7.23 μg·g^-1^, respectively (Fig. 7E).

Taken together, these findings suggest that the high accumulation of dammaradienol in *O. elatus* does not necessarily imply flux into canonical RK-type ginsenosides. Instead, dammaradienol may represent a branch-point intermediate that can either accumulate directly or undergo alternative modifications, potentially including storage in less polar forms such as acetylated derivatives, as reported for a related *Oplopanax* species^29^. Meanwhile, the absence of RK-type ginsenosides in *O. elatus* is likely to reflect not only the genomic loss of the C12 hydroxylase step, but also spatiotemporal mismatch between upstream and downstream pathway genes, because *OeOSC14* is predominantly expressed in leaves, whereas several candidate downstream *UGTs* are mainly expressed in roots (Fig. 1 and Supplementary Fig. 38).

The strong leaf accumulation of dammaradienol further raises the possibility that this scaffold, or its yet-unidentified derivatives, may itself have ecological or physiological functions, for example, in defense or stress adaptation^2^. From a synthetic biology perspective, the reconstruction of RK-type ginsenoside biosynthesis in *N. benthamiana* demonstrates the feasibility of producing Rk1, Rk2, and Rk3 de novo in a plant chassis. However, the markedly lower yields of the final glycosylated products compared with the upstream dammaradienol scaffold indicate that downstream pathway steps remain major bottlenecks. In particular, the decrease from ~3.63 mg g^-1^ DW dammaradienol to 23.07 μg g^-1^ DW Rk3, with even lower yields of Rk1 and Rk2, suggests that CYP-mediated hydroxylation and/or UGT-mediated glycosylation may operate with limited efficiency under the transient expression conditions tested here. These results indicate that the current pathway reconstruction is more suitable as a proof-of-concept platform than as an optimized production system. Future improvements should focus on identifying more efficient hydroxylases and glycosyltransferases, engineering the catalytic properties of existing CYPs and UGTs, balancing the expression levels of pathway enzymes, and enhancing metabolic flux from dammaradienol toward hydroxylated and glycosylated intermediates. In this context, the introduction of *OeOSC14* into *Panax*, which possesses endogenous downstream tailoring capacity but lacks a native dammaradienol synthase step, may provide an alternative route to enable RK-type ginsenoside formation in a more compatible metabolic background.

This study uncovered a chromosome-level genome of *O. elatus*, a close relative of *Panax* species, and revealed the evolutionary and genetic basis underlying the divergence of dammaradienol-derived RK-type ginsenoside biosynthesis within Araliaceae. We show that *O. elatus* evolved a specialized dammaradienol synthase (OeOSC14) from an ancestral mTTS through gene duplication, neofunctionalization, and subsequent functional specialization, while the downstream pathway is interrupted by loss of the conserved C12-hydroxylase loci, preventing oxidative tailoring and thereby driving dammaradienol accumulation. Guided by these genome-enabled insights, we further reconstructed the complete pathway in *N. benthamiana* and achieved de novo biosynthesis of ginsenosides Rk1, Rk2, and Rk3. Together, the elucidation of key enzymatic innovations and missing tailoring steps in *O. elatus* provides important insights into triterpene synthase evolution and establishes a tractable plant chassis for sustainable production of pharmaceutically valuable RK-type ginsenosides. More broadly, the high-quality *O. elatus* genome generated here provides an important foundation for future identification of the *CYPs* and *UGTs* responsible for the biosynthesis of other native triterpenoid saponins, including cirenshenosides S, T, U, and V.

## Methods

### Plant materials, DNA extraction and sequencing

A healthy individual of *O. elatus* (3 years old) used in this study was collected from a cultivated population in Harbin, Heilongjiang Province, China. The population was cultivated in a shaded area outdoors, protected from direct sunlight. Fresh and healthy plants were selected, carefully excavated from the soil with the root system intact, rapidly cleaned, and immediately frozen in liquid nitrogen, and then preserved at −80 °C for subsequent experiments. The specific appearance of each tissue type is shown in Fig. 1B. High-quality DNA was isolated from fresh young leaves using the cetyltrimethylammonium bromide (CTAB) method. For the generation of PacBio HiFi long-read data, a total of 15 µg of high-molecular-weight (HMW) DNA was sheared using gTUBEs, following which a standard PacBio SMRTbell library was constructed using the SMRTbell Express Template Prep Kit 2.0. The resulting library was size-selected using a BluePippin system with a cutoff of 15 kb to remove short DNA fragments. HiFi consensus sequences were subsequently generated on the PacBio Sequel II platform. Following the standard Illumina library construction, the Hi-C library was quantified and sequenced on the MGIseq-2000 sequencing platform.

### **Genome** de novo **assembly and quality control**

The *O. elatus* genome was assembled using PacBio HiFi reads combined with Hi-C data. First, the raw Hi-C data were filtered using SOAPnuke v.1.5.3^49^ to obtain high-quality clean reads (parameters: filter -n 0.01 -l 20 -q 0.4 -d -M 3 -A 0.3 -q 2 -i -G -seqType 1). The HiFi CCS reads were assembled into haplotype-phased contigs using hifiasm v.0.16.1^50^, with Hi-C data incorporated to assist in the assembly process. To anchor the contigs to chromosomes, Hi-C data were utilized through Juicer v.1.6^51^ and 3D-DNA v.180922^52^ (parameters: -m haploid --early-exit -q20), which performed filtering, ordering, and orientation of the contigs. Finally, the assembly was manually curated using Juicebox v.1.11.08^51^ to correct misjoins and further refine the contig order and orientation, resulting in a high-quality chromosome-level genome assembly.

Genome completeness was assessed using BUSCO v.6.0.0^53^.

### Transposable elements annotation

To identify repetitive sequences in the *O. elatus* genome, we employed a combination of de novo and homology-based approaches. First, a non-redundant repeat sequence database was constructed using LTR_Finder v.1.07^54^, LTRdigest^55^, and RepeatModeler v.2.0.7 (http://www.repeatmasker.org/RepeatModeler/). RepeatMasker was then applied with the de novo-derived repeat database to detect repetitive elements. For homology-based identification, RepeatMasker and RepeatProteinMask were employed against the Repbase database to annotate known repeat elements. Additionally, tandem repeats within the genome were identified and annotated using TRF v.4.09.1^56^. The annotation results of repetitive sequences for assembled genome, including total repeat content and detailed family composition, are provided in Supplementary Tables 8 and 9.

### Genome annotation

Gene prediction was performed by integrating three complementary approaches: homology-based prediction, de novo prediction, and transcriptome-based prediction using RNA-seq data. For homology-based prediction, protein sequences from closely related species were aligned to the assembled genome using TBLASTN v.2.2.31 + ^57^ (E-value < 1e-5). The best alignment results and corresponding gene structures were then identified using Exonerate v.2.4.0 (https://github.com/nathanweeks/exonerate) with the protein2genome model (--model protein2genome). In the de novo prediction approach, repetitive regions in the genome were first masked using RepeatMasker. An HMM model was then trained using the gene structures predicted by the homology-based method. Subsequently, Augustus v.3.5.0^58^ was employed to predict gene structures based on the trained HMM model. For transcriptome-based prediction, RNA-seq data were first aligned to the genome using HISAT2 v.2.2.1^59^. The aligned reads were then assembled into transcripts using StringTie v.2.2.3^60^. Splice sites were validated and transcript models were further refined using PASA_lite v.0.1.0 (https://github.com/PASApipeline/PASA_Lite). Finally, the gene models predicted by the three methods were integrated using MAKER2^61^ to generate a high-quality gene set. For functional annotation, protein sequences of predicted genes were compared against the SwissProt^62^, KEGG^63^, KOG^64^, and NR^65^ databases using BLASTP (E-value < 1e-10). Functional annotations were assigned based on the best BLASTP hits.

### Genome evolution

Comparative genome analysis was conducted using *O. elatus* and 18 additional plant species: *V. vinifera*, *Arabidopsis thaliana*, *Populus trichocarpa*, *A. elata*, *P. stipuleanatus*, *P. notoginseng*, *P. quinquefolium*, *P. ginseng*, *P. japonicus*, *E. senticosus*, *Daucus carota*, *Apium graveolens*, *Coriandrum sativum*, *Helianthus annuus*, *Coffea canephora*, *Solanum lycopersicum*, *Brachypodium distachyon*, *Oryza sativa*. Gene families were clustered using OrthoFinder v.2.5.5^66^ with default parameters. To infer phylogenetic relationships, phase 1 or fourfold degenerate (4D) sites were extracted based on single-copy gene family identified. A maximum likelihood (ML) phylogenetic tree was then reconstructed using RAxML v.8.2.12^67^ under the GTRGAMMA model with 1000 bootstrap replicates (-m GTRGAMMA -f a -k -#1000). The fourfold degenerate sites from single-copy orthologs were further used to estimate species divergence times using the MCMCTree program in the PAML v4.9j package^68^. Divergence times were inferred under a Bayesian framework with a Markov chain Monte Carlo (MCMC) algorithm. The following parameters were used in MCMCTree: nucleotide substitution model – REV; molecular clock model – correlated rates; burn-in iterations – 20,000; sample frequency – 5000; number of samples – 20,000. Calibration points were based on divergence times obtained from the TimeTree database (https://timetree.org/home). Gene family expansions and contractions were analyzed using CAFE v.4.2.1^69^ (-p 0.05 -t 20 -filter).

For intraspecific paralogous gene pairs, syntenic blocks were first identified using MCScanX v.1.0.0^70^ (-k 50 -s 5 -e 1e-05 -m 25), and *K*s values between gene pairs within each syntenic block were calculated using codeml in PAML under the F3X4 model. Interspecific orthologous gene pairs were identified by BLASTP (E-value < 1e-5), and best-hit gene pairs were selected based on bit-score and E-value rankings to calculate *K*s values using PAML.

### Identification of candidate genes

Gene family prediction was conducted using HMMER v.3.1b2^71^ based on Pfam models PF13243 and PF13249 (for OSC), PF00067 (for CYP), and PTHR48047 (for UGT) in *O. elatus*, with an E-value threshold of <0.01. The predicted protein sequences were further filtered using SeqKit v.2.6.1^72^ software according to their lengths: 500–900 amino acids for OSCs, and 400–600 amino acids for CYPs and UGTs. The filtered gene family members were subsequently validated using the online tool Conserved Domain Database (https://www.ncbi.nlm.nih.gov/Structure/cdd/wrpsb.cgi).

### Phylogenetic analysis

To infer the phylogenetic history of gene families including OSC, CYP, and UGT, and to determine their subfamily classifications, we performed phylogenetic analysis using gene sequences with reported functions in plants, together with predicted family members from *O. elatus*. All sequences used in the analyses are listed in Supplementary Data 3–6. Multiple sequence alignments were performed using MAFFT v.7.518^73^ with default parameters. A maximum-likelihood (ML) phylogenetic tree was reconstructed using FastTree v.2.2^74^ software under default settings. The resulting tree in Newick format was visualized and annotated using the Interactive Tree Of Life (iTOL) online platform (https://itol.embl.de/).

### **Construction of plant gene expression vectors**

Total RNA was extracted using an EasyPure® Plant RNA Kit (TransGen, Beijing, China). The candidate genes were amplified from the cDNA library from total RNA and cloned into pEAQ-HT plasmid using homologous recombination method (ClonExpress II One Step Cloning Kit-C112, Vazyme, Nanjing, China). A list of plasmids is provided in Supplementary Data 7. Plasmid PCR using point mutations primers was employed to construct the vector carrying the coding gene bearing a site-directed mutation. The primers of gene cloning and point mutations were listed in Supplementary Data 8. Sequence-verified vectors were transformed into *Agrobacterium tumefaciens* GV3101 Chemically Competent Cells (Weidi, Shanghai, China).

### Transcriptome analysis

Total RNA was extracted from 12 samples representing different tissue types of *O. elatus* using the TransZol Up Plus RNA Kit (TransGen, China). The qualified libraries were subjected to 150-bp paired-end sequencing based on the DNBSEQ-T7 platform (BGI, Shenzhen, China). After filtering and trimming the raw reads using Trimmomatic v.0.39^75^ and FastQC v.0.11.9 (www.bioinformatics.babraham.ac.uk), the clean RNA-seq data were aligned to the reference genome of *O. elatus* using HISAT2 v.2.2.1 with the following parameters: --dta-cufflinks. The resulting BAM files were sorted and indexed by SAMtools v1.16.1^76^ with default parameters. Gene expression was quantified using FeatureCounts v1.6.4^77^ with default parameters, and the expression matrix was constructed based on fragments per kilobase of transcript per million mapped reads (FPKM).

### **Transient expression in*N. benthamiana***

Positive single colonies of *A. tumefaciens* GV3101 carrying pEAQ expression vector containing candidate genes and *tHMGR* gene, a feedback-insensitive form of HMG-CoA reductase, were cultured in 10 mL LB liquid medium with 20 μg/mL rifampicin, and 50 μg/mL kanamycin at 28 °C with shaking at 180 rpm until the optical density 600 (OD₆₀₀) reached 0.8. Bacterial pellets were resuspended in infiltration buffer (10 mM MES, 10 mM MgCl₂, 150 μM acetosyringone, pH 5.6) to a final OD₆₀₀ of 0.2. The mixed Agrobacterium culture was incubated at room temperature for 3 hours before infiltration. Healthy, young leaves of tobacco at the six-leaf stage were infiltrated with the bacterial suspension on the abaxial side of the leaves using a 1 mL syringe without a needle. After six days of co-cultivation under a 14 h light/10 h dark photoperiod at 26 °C, the infiltrated tobacco leaves were harvested and freeze-dried for subsequent extraction and analysis.

### Extraction and quantification of triterpenes

Plant tissue samples stored at −80 °C were lyophilized to complete dryness using a vacuum freeze dryer. A total of 0.1 g of *O. elatus* tissue (0.02 g for leaf tissue of *N. benthamiana*) was transferred into a 2 mL microcentrifuge tube, followed by the addition of 1 mL saponification reagent (1 g KOH dissolved in 10 mL anhydrous ethanol). The mixture was vortexed vigorously and incubated in a metal bath at 75 °C for 1 h. After evaporation of the ethanol, 500 μL of ethyl acetate was added to extract the triterpenoids. The mixture was vortexed for 30 min, followed by the addition of 500 μL water and further vortexing. The sample was then centrifuged at 12,000 × g for 1 min, and 100 μL of the upper organic phase was transferred into a MS-grade glass vial. The solvent was evaporated completely, and the residue was derivatized with 50 μL N-Methyl-N-(trimethylsilyl) trifluoroacetamide reagent (Sigma Aldrich) at 70 °C for 30 min prior to GC–MS analysis.

Quantification analysis of triterpenoids was performed using an external standard method. Standard curves were generated for each reference compound, and the absolute content of the corresponding compounds in the samples was calculated based on the peak areas.

### GC–MS analysis

GC–MS analysis was performed using a Nexis GC-2030 gas chromatography system coupled with a TQ8050 NX mass spectrometer (Shimadzu, Japan). An Rtx-5MS capillary column (30 m × 0.25 mm × 0.25 μm, Shimadzu) was used for the separation of triterpenes. Injections (1 μL) were performed in split mode with the port temperature set to 250 °C. The GC oven temperature program was programmed as follows: the initial temperature was set at 50 °C and held for 2 min with subsequent increase to 290 °C at a rate of 4 °C/min and held at 290 °C for 4 min, and finally rose to 330 °C at a rate of 20 °C/min and maintained it for 4 min. The total run time of the GC–MS program was 42 min. The mass spectrometer parameters were as follows: ion-source temperature was 230 °C and mass scan range was 60–800 *m*/*z* with a scan range time of 0.3 s. Data analysis was carried out using GCMSsolution 4.54 (Shimadzu).

### **Purification of leaf extracts from*O. elatus***

Triterpenes from the leaves of *O. elatus* were extracted using the saponification reagent (1 g KOH dissolved in 10 mL anhydrous ethanol). The extraction was scaled up at a ratio of 1 mL saponification reagent per 50 mg of dried sample. After evaporation, the residue was dissolved in an equal volume of ethyl acetate, followed by washing with an equal volume of saturated sodium chloride solution to remove pigments and other impurities. The ethyl acetate layer was then evaporated to dryness. The crude extract was further purified using a preparative high-performance liquid chromatography (HPLC) system equipped with a Welch XB-C18 column (10 × 250 mm, 5 μm). Chromatographic separation of dammaradienol was carried out using a mobile phase composed of acetonitrile (B) and water (A) at a flow rate of 5 mL/min. The gradient program was as follows: 30% B from 0 to 10 min; 30%–70% B from 10 to 30 min; 70%–90% B from 30 to 55 min; 90%–100% B from 55 to 70 min; 100% B from 70 to 90 min; 100%–90% B from 90 to 100 min; 90%–70% B from 100 to 105 min; and 70%–30% B from 105 to 120 min. Fractions were collected every 16 mL, resulting in a total of 38 fractions. These were analyzed by GC–MS to identify the fraction containing dammaradienol and assess its purity. Fraction 28 was identified as containing the target compound and was subjected to repeated collection and lyophilization to obtain a highly purified dammaradienol fraction.

### Nuclear magnetic resonance (NMR) spectroscopy

The structure of dammaradienol was elucidated using 1D and 2D NMR spectroscopy. The lyophilized samples (15 mg) were dissolved in CDCl₃, and NMR spectra (^1^H and ^13^C NMR, ^1^H-^1^H COSY, NOESY, HMBC and HSQC) were recorded at 25 °C on a Bruker AVANCE 600 NMR spectrometer operating at 600 MHz for ^1^H and 150 MHz for ^13^C NMR. All chemical shifts (*δ*) are given in ppm units with reference to tetramethylsilane (TMS) as internal standard. NMR data were processed using Bruker Topspin v.4.1.4 and analyzed with MestReNova v.15.0.1 software.

### LC–MS analysis

For metabolic extraction from plant tissues, 100 mg of the lyophilized samples were homogenized using 3-mm tungsten carbide beads (Qiagen, Hilden, Germany) in a TissueLyser (25 Hz, 1 min), followed by the addition of 1 mL of 80% (v/v) LC–MS grade methanol. The mixture was sonicated for 1 h at 37 °C and then shaken at 37 °C and 600 rpm for 6 h. After centrifugation at 16,700 × g for 2 min, 500 μL of the supernatant was transferred to a new microcentrifuge tube and lyophilized. The dried extracts were reconstituted in 200 μL of 80% (v/v) methanol, filtered through a 0.22 μm membrane filter, and transferred into glass inserts within 2 mL glass vials for LC–MS analysis.

LC–MS analysis was performed using an LC–MS 9030 quadrupole time-of-flight liquid chromatograph mass spectrometer (Shimadzu, Japan). Chromatographic separation was performed using a Welch C18 column (2.1 × 100 mm, 1.8 μm), with a mobile phase consisting of acetonitrile (B) and 0.1% formic acid in water (A) at a flow rate of 0.2 mL/min. The linear gradients of solvent B as follows: 35% from 0 to 1 min; 35%-95% from 1 min to 12 min; 95% from 12 min to 17 min; 95%-35% from 17 min to 21 min. The column oven temperature was set at 40 °C. The total time of program was 21 min. Mass spectrometry analysis was carried out in data-dependent acquisition (DDA) mode using negative ionization mode of electrospray ionization (ESI), monitoring full spectra from *m*/*z* 100–1500 (100 ms per spectrum) and the data-dependent MS/MS spectra (60 ms per spectrum) with 35% collision energy (CE). Data analysis was carried out using Labsolutions insight explore (Shimadzu). A list of standards is provided in Supplementary Data 9.

### Intergenic microcollinearity analysis

To identify syntenic blocks and orthologous gene pairs between different species, comparative genomic analysis was conducted using the Python-based JCVI toolkit v.1.3.3^78^, based on GFF annotation files and FASTA-formatted CDS sequences. Microsynteny around the target genes was then visualized using the jcvi.graphics.synteny subcommand.

### Structure modeling, molecular docking, and site-directed mutagenesis

Structural models of OeOSC14 and its mutants were predicted using AlphaFold 3 (https://deepmind.google/science/alphafold/). AutoDockTools v.1.5.7^79^ was employed for protein and ligand preparation, including the removal of water molecules and the addition of hydrogen atoms. Subsequently, the ligands were docked into the active pocket using AutoDock3 with default docking parameters. The protein-ligand complexes were analyzed and visualized using PyMOL v.1.8 (https://www.pymol.org/). Key active-site residues were identified by integrating the amino acids located within 5 Å of the ligand and multiple sequence alignments across the OSC subfamily members, followed by site-directed mutagenesis. Complementary mutagenic primers were designed for whole-plasmid PCR amplification. The resulting PCR products were treated with *Dpn I* endonuclease at 37 °C for 30 minutes to remove the wild-type plasmid template, yielding vectors carrying the OSC mutants.

### Quantum-chemical calculations

Structural optimizations, frequency calculations, and the single point energy calculations for the dammarenyl cations in water were performed through M06-2X/6-311 G** method with Gaussian16 program suite. The implicit solvent model SMD was used for all the calculations. It should be noted that simultaneous isomerization was observed for dammarenyl cation II conformer, and thus the structure for such a conformer was selected as the one with the lowest energy just before isomerization.

### Statistics and reproducibility

Quantitative analyses including transcriptomics, metabolic profiling by GC–MS and LC–MS were performed with three independent biological replicates and presented as mean ± standard error (s.e.m.). No statistical methods were used to predetermine sample size. All data were included in the analysis without exclusion. The investigators were not blinded to group assignments or outcome assessment throughout the experiments.

### Reporting summary

Further information on research design is available in the Nature Portfolio Reporting Summary linked to this article.

## Supplementary information

Supplementary Information

Peer Review file

Description of Additional Supplementary Files

Supplementary Data 1

Supplementary Data 2

Supplementary Data 3

Supplementary Data 4

Supplementary Data 5

Supplementary Data 6

Supplementary Data 7

Supplementary Data 8

Supplementary Data 9

Reporting Summary

## Source data

Source Data

## Data Availability

The raw sequence data for the PacBio HiFi reads, RNA-seq reads, genome assembly, and annotation of *O. elatus* generated in this study have been deposited in the National Genomics Data Center, Beijing Institute of Genomics, Chinese Academy of Sciences/China National Center for Bioinformation under BioProject number PRJCA054934. Source data are provided with this paper.
