# Supplementary material for: The Oplopanax elatus genome reveals dammaradienol synthase evolution enabling reconstruction of RK type ginsenosides biosynthesis
Source: Nat Commun. 2026 Jun 24;17:7907. doi: 10.1038/s41467-026-74686-6 (PMC13444123; doi:10.1038/s41467-026-74686-6)
Supplement: Supplementary file 1 [file 41467_2026_74686_MOESM13_ESM.pdf]

nature portfolio

Corresponding author(s): Yu Wang

Last updated by author(s): Dec 31, 2025

Reporting Summary

Nature Portfolio wishes to improve the reproducibility of the work that we publish. This form provides structure for consistency and transparency in reporting. For further information on Nature Portfolio policies, see our Editorial Policies and the Editorial Policy Checklist.

Statistics

For all statistical analyses, confirm that the following items are present in the figure legend, table legend, main text, or Methods section.

n/a	Confirmed	
☐	☒	The exact sample size (*n*) for each experimental group/condition, given as a discrete number and unit of measurement
☐	☒	A statement on whether measurements were taken from distinct samples or whether the same sample was measured repeatedly
☒	☐	The statistical test(s) used AND whether they are one- or two-sided<br>*Only common tests should be described solely by name; describe more complex techniques in the Methods section.*
☒	☐	A description of all covariates tested
☒	☐	A description of any assumptions or corrections, such as tests of normality and adjustment for multiple comparisons
☐	☒	A full description of the statistical parameters including central tendency (e.g. means) or other basic estimates (e.g. regression coefficient) AND variation (e.g. standard deviation) or associated estimates of uncertainty (e.g. confidence intervals)
☒	☐	For null hypothesis testing, the test statistic (e.g. *F*, *t*, *r*) with confidence intervals, effect sizes, degrees of freedom and *P* value noted<br>*Give P values as exact values whenever suitable.*
☒	☐	For Bayesian analysis, information on the choice of priors and Markov chain Monte Carlo settings
☒	☐	For hierarchical and complex designs, identification of the appropriate level for tests and full reporting of outcomes
☒	☐	Estimates of effect sizes (e.g. Cohen's *d*, Pearson's *r*), indicating how they were calculated

Our web collection on statistics for biologists contains articles on many of the points above.

Software and code

Policy information about availability of computer code

Data collection	HPLC/MS: LC-MS 9030 system (Shimadzu), Labsolutions insight explore (Shimadzu), GC/MS: Nexis GC-2030 gas chromatography system/ TQ8050 NX mass spectrometer (Shimadzu, Japan), NMR: AscendTM 600 MHz spectrometer (Bruker)
Data analysis	K-mer analysis: SOAPnuke(v1.5.3), JELLYFISH(v.2.2.6), GenomeScope;<br>Genome assembly: Hifiasm (v0.16.1), SOAPnuke (v1.5.3), Juicer (V1.6), 3d-DNA (v180922), Juicerbox (v1.11.08), BUSCOv6 (embrophyta_odb10);<br>Genome annotation: AUGUSTUS (v3.5.0), HISAT2 (V2.2.1), StringTie(v2.2.2), PASA_lite (v0.1.0), MAKER(v3.31.8), InterProscan (v5,16-55.0), RepeatMasker (v4.), RepeatProteinMasker (v4.0.7), RepeatModeler (v2.0.7), Tandem Repeat Finder (v4.09.1),HMMER3, BLAST(V2.2.31), LTR_Finder (v1.07), LTRdigest, Exonerate (v2.4.0);<br>Gene function annotation databases: KEGG, SwissProt, NR, KOG, GO, Repbase v21.12 library;<br>Gene evolution analysis software: OrthoFinder (v2.5.5), CAFE (v4.2.1), MUSCLE (v3.8.31), RAxML (v8.2.12), PAML v4.9j package, MCScanX (v1.0.0);<br>Transcriptome analysis: Trimmomatic(vO.39), FastQC(vO.11.9), HISAT2 (v2.2.1), SAMtools (v1.16.1), FeatureCounts (v1.6.4);<br>Phylogenetic analysis: MAFFT (v7.518), FastTree (v2.2), iTOL(https://itol.embl.de/upload.cgi);<br>Quantification data analysis to make figures: Microsoft Excel;<br>Intergenic microcollinearity analysis: Python-based JCVI toolkit (v.1.3.3);<br>Molecular docking: AlphaFold 3 (https://deepmind.google/science/alphafold/), AutoDockTools (v.1.5.7), PyMOL (v.1.8);<br>Editing of figures: Adobe llustrator (v2020), ChemDraw (v14.0).

For manuscripts utilizing custom algorithms or software that are central to the research but not yet described in published literature, software must be made available to editors and reviewers. We strongly encourage code deposition in a community repository (e.g. GitHub). See the Nature Portfolio guidelines for submitting code & software for further information.

Data

Policy information about availability of data

All manuscripts must include a data availability statement. This statement should provide the following information, where applicable:

- Accession codes, unique identifiers, or web links for publicly available datasets
- A description of any restrictions on data availability
- For clinical datasets or third party data, please ensure that the statement adheres to our policy

The raw sequence data for the PacBio HiFi reads, RNA-Seq reads, genome assembly, and annotation of Oplopanax elatus generated in this study have been deposited at the National Genomics Data Center, Beijing Institute of Genomics, Chinese Academy of Sciences/China National Center for Bioinformation under BioProject number PRJCA054934.

Research involving human participants, their data, or biological material

Policy information about studies with human participants or human data. See also policy information about sex, gender (identity/presentation), and sexual orientation and race, ethnicity and racism.

Reporting on sex and gender	This study does not involve human participants, human data, or human biological materials. Therefore, considerations related to sex or gender are not applicable.
Reporting on race, ethnicity, or other socially relevant groupings	This study does not involve human participants or socially constructed groupings such as race or ethnicity. As the research focuses exclusively on plant genomics, this item is not applicable.
Population characteristics	No human population characteristics were collected or analyzed in this study, as the research is based entirely on plant genomic, transcriptomic, and functional data.
Recruitment	This study did not involve the recruitment of human participants. Recruitment procedures and related biases are therefore not applicable.
Ethics oversight	Ethical approval for human or animal research was not required, as this study involves only plant materials and publicly accepted experimental systems.

Note that full information on the approval of the study protocol must also be provided in the manuscript.

Field-specific reporting

Please select the one below that is the best fit for your research. If you are not sure, read the appropriate sections before making your selection.

☒ Life sciences ☐ Behavioural & social sciences ☐ Ecological, evolutionary & environmental sciences

For a reference copy of the document with all sections, see nature.com/documents/nr-reporting-summary-flat.pdf

Life sciences study design

All studies must disclose on these points even when the disclosure is negative.

Sample size	One sample of Oplopanax elatus was used for genome sequencing; For RNA-seq three biological samples were used; For experiments determing metabolic contents three biological independent samples were used. All these samples were wild-type and were collected from the planting areas of O. elatus.
Data exclusions	For genome assembly, sequences with low-quality were excluded; We excluded the amino acids sequences that were lower than 300 aa for constructing the phylogenetic trees of OSCs, P450s, and UGTs.
Replication	For RNA-seq, three biological replicates were used; For metabolic analysis, three biological replicates were used.
Randomization	No method of randomization was used. No random sampling is required for genome sequencing, because the genome differences are very small within the wild population, thus any wild plant is allowed for genome sequencing; Biological replicates of RNA-seq were performed in separate experiments at different times to control for co-variates.
Blinding	Blinding is not applicable in our study because it does not involve subjects which receive different treatments. All experiments were done by analyzing data derived from different biological replicates directly.

Reporting for specific materials, systems and methods

We require information from authors about some types of materials, experimental systems and methods used in many studies. Here, indicate whether each material, system or method listed is relevant to your study. If you are not sure if a list item applies to your research, read the appropriate section before selecting a response.

Materials & experimental systems

n/a	Involved in the study
☒	☐ Antibodies
☒	☐ Eukaryotic cell lines
☒	☐ Palaeontology and archaeology
☒	☐ Animals and other organisms
☒	☐ Clinical data
☒	☐ Dual use research of concern
☐	☒ Plants

Methods

n/a	Involved in the study
☒	☐ ChIP-seq
☒	☐ Flow cytometry
☒	☐ MRI-based neuroimaging

Dual use research of concern

Policy information about dual use research of concern

Hazards

Could the accidental, deliberate or reckless misuse of agents or technologies generated in the work, or the application of information presented in the manuscript, pose a threat to:

No	Yes
☒	☐ Public health
☒	☐ National security
☒	☐ Crops and/or livestock
☒	☐ Ecosystems
☒	☐ Any other significant area

Experiments of concern

Does the work involve any of these experiments of concern:

No	Yes
☒	☐ Demonstrate how to render a vaccine ineffective
☒	☐ Confer resistance to therapeutically useful antibiotics or antiviral agents
☒	☐ Enhance the virulence of a pathogen or render a nonpathogen virulent
☒	☐ Increase transmissibility of a pathogen
☒	☐ Alter the host range of a pathogen
☒	☐ Enable evasion of diagnostic/detection modalities
☒	☐ Enable the weaponization of a biological agent or toxin
☒	☐ Any other potentially harmful combination of experiments and agents

Plants

Seed stocks	The Oplopanax elatus plants were collected from Changbai Mountain, Jilin Province, China, with sampling conducted on June 30, 2022.
Novel plant genotypes	No novel plant genotypes were generated in this study.
Authentication	No seed stock was used in this study; No mutation plant generated in this study.
