# Supplementary material for: The Oplopanax elatus genome reveals dammaradienol synthase evolution enabling reconstruction of RK type ginsenosides biosynthesis
Source: Nat Commun. 2026 Jun 24;17:7907. doi: 10.1038/s41467-026-74686-6 (PMC13444123; doi:10.1038/s41467-026-74686-6)
Supplement: Supplementary file 2 [file 41467_2026_74686_MOESM1_ESM.pdf]

The *Oplopanax elatus* genome reveals dammaradienol synthase evolution enabling reconstruction of RK type ginsenosides biosynthesis

Wang *et al.*

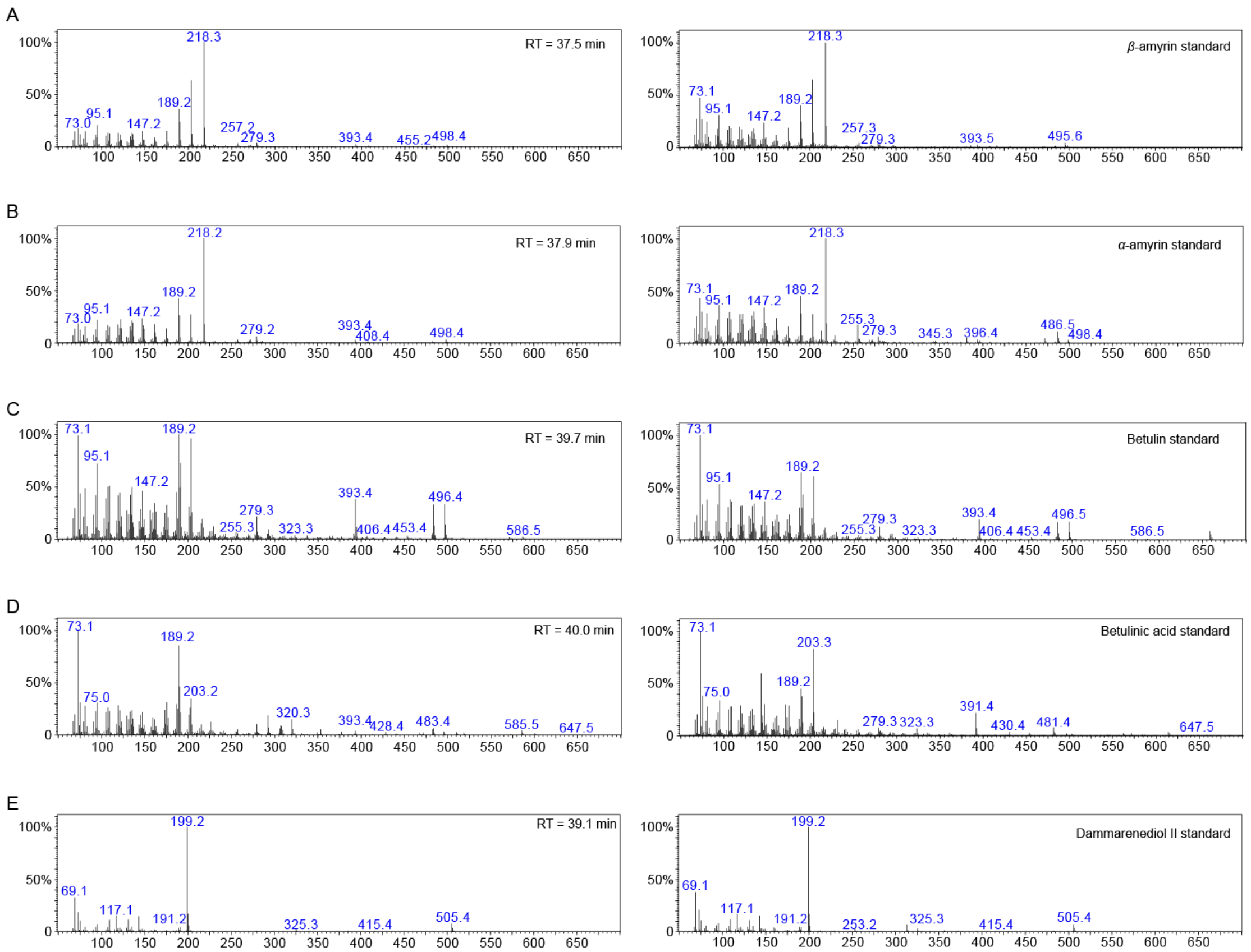

Supplementary Figure 1. Mass spectra for triterpene identified in leaves of *O. elatus* by GC-MS. (A) Mass spectra of *β*-amyrin identified in *O. elatus* and their corresponding reference standards by GC-MS. (B) Mass spectra of α-amyrin identified in *O. elatus* and their corresponding reference standards by GC-MS. (C) Mass spectra of betulin identified in *O. elatus* and their corresponding reference standards by GC-MS. (D) Mass spectra of betulinic acid identified in *O. elatus* and their corresponding reference standards by GC-MS. (E) Mass spectra of dammarenediol II identified in *O. elatus* and their corresponding reference standards by GC-MS. Mass spectra comparing the TMS derivatized triterpenoids identified in *O. elatus* (left panel) with their corresponding authentic standards (right panel).

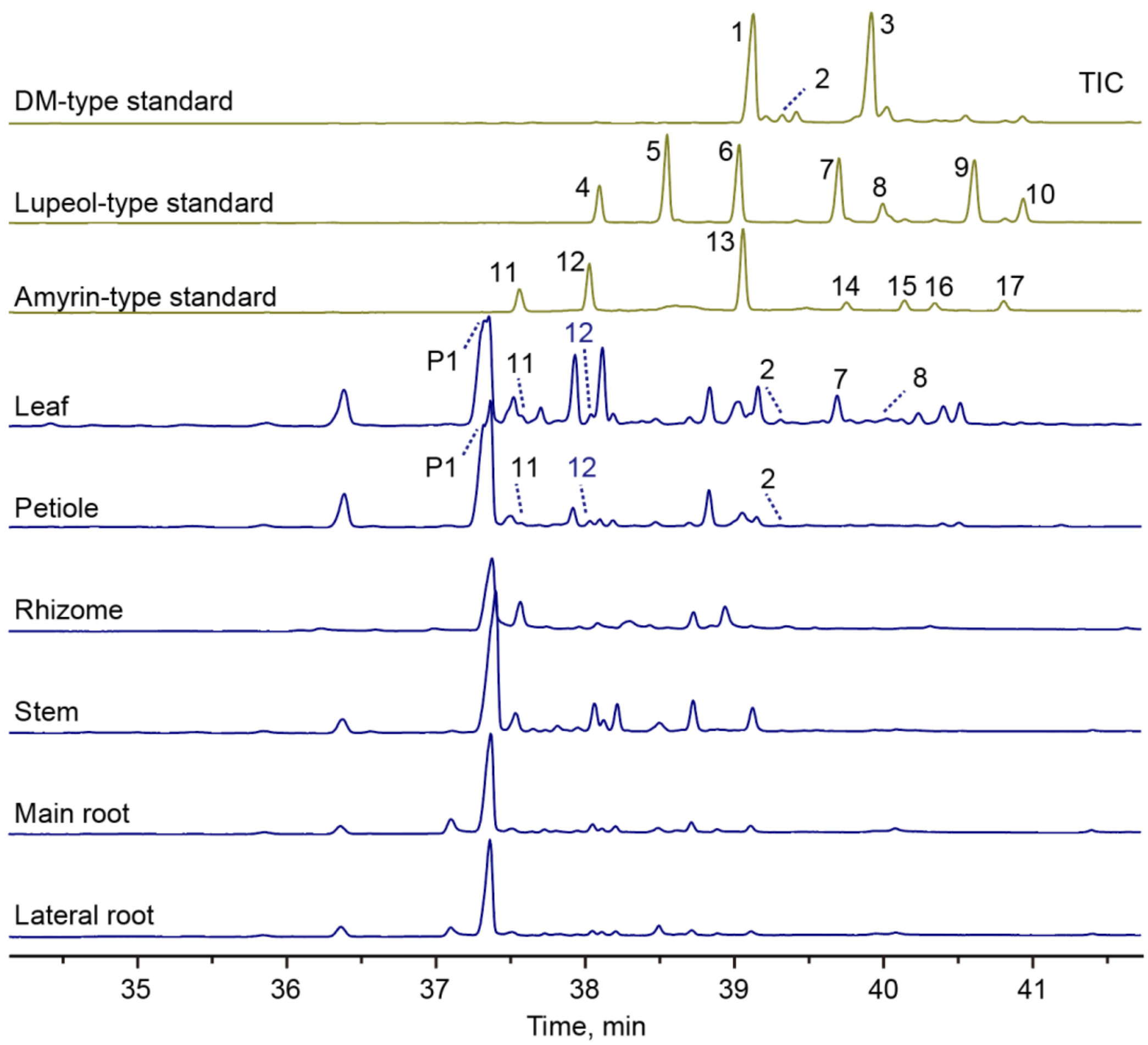

Supplementary Figure 2. Metabolite analysis of *O. elatus* by GC-MS. Total ion chromatograms (TIC) from GC-MS analysis of extracts from different tissues of *O. elatus,* including leaf, petiole, rhizome, stem, main root, and lateral root. DM-type standard: 1, protopanaxadiol; 2, dammarenediol II; 3, protopanaxatriol. Lupeol-type standard: 4, lupeol; 5, α-23-hydroxylupeol; 6, β-23-hydroxylupeol; 7, betulin; 8, betulinic acid; 9, 23-hydroxybetulin; 10, 23-hydroxybetulinic acid. Amyrin-type standard: 11, β-amyrin; 12, α-amyrin; 13, erythrodiol; 14, oleanolic acid; 15, echinocystic acid; 16, ursolic acid; 17, hederagenin.

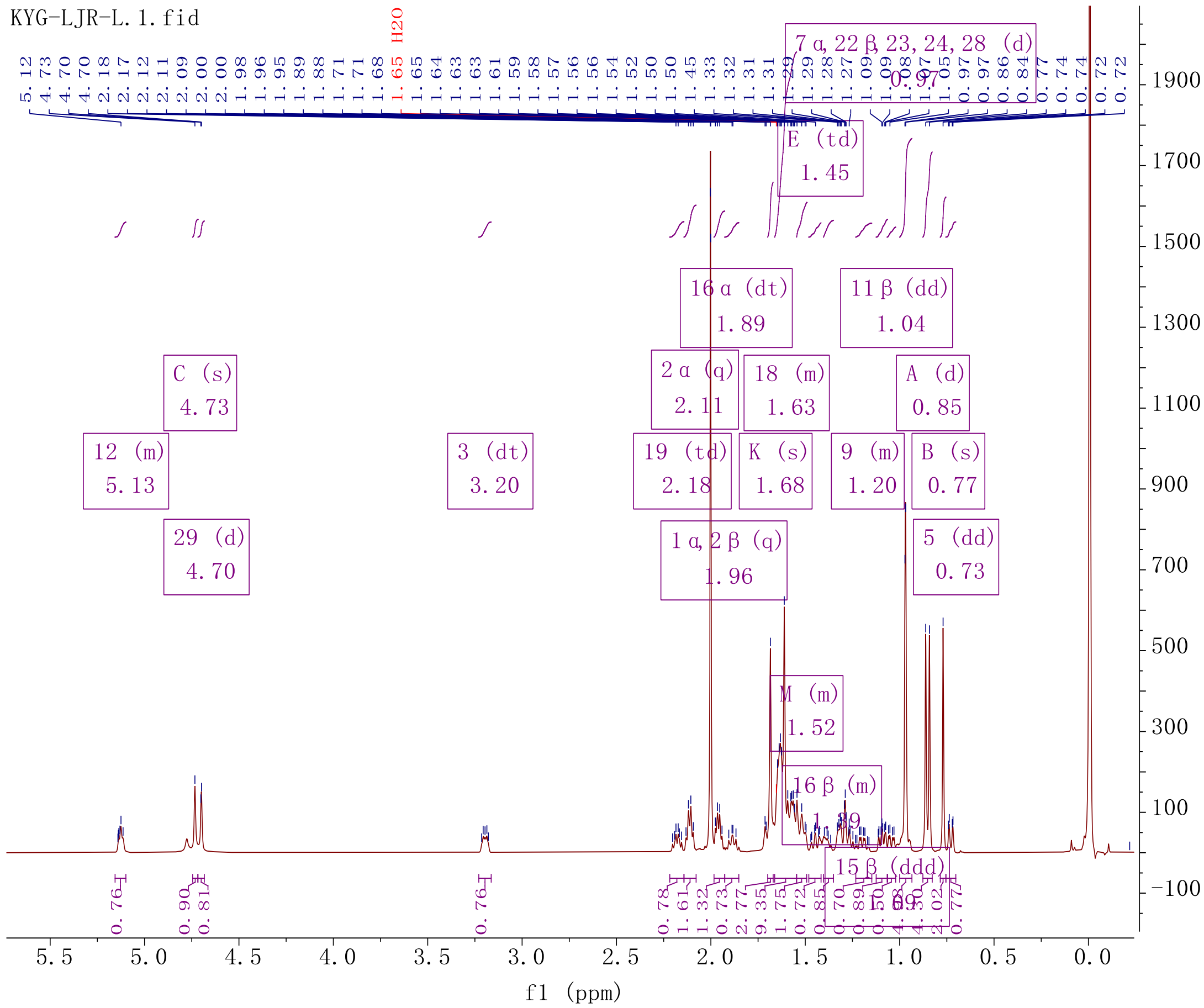

Supplementary Figure 3. ^{1}H-NMR spectrum of dammaradienol in $CDCl_3$ (600 MHz).

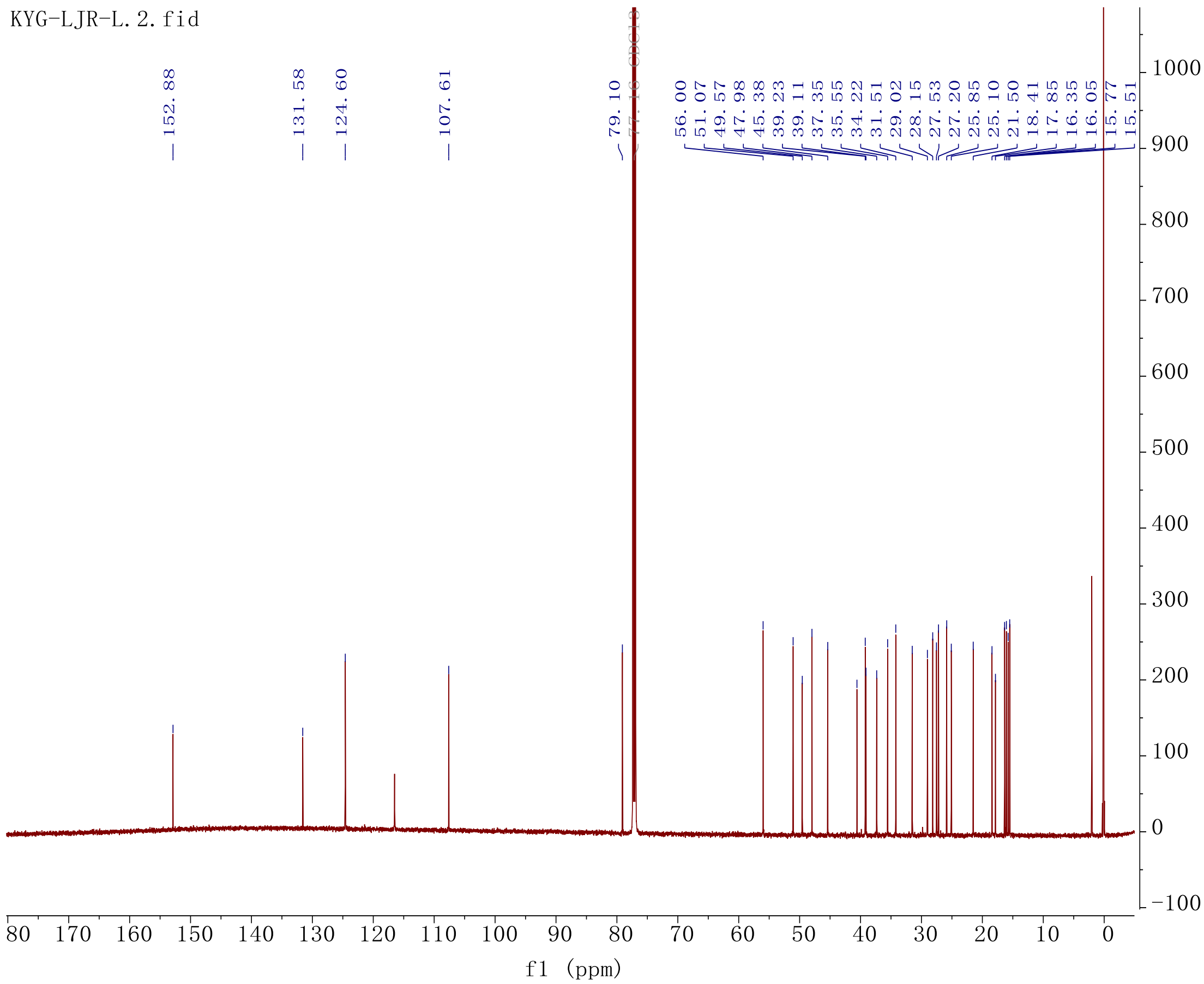

Supplementary Figure 4. ^{13}C-NMR spectrum of dammaradienol in $CDCl_3$ (150 MHz).

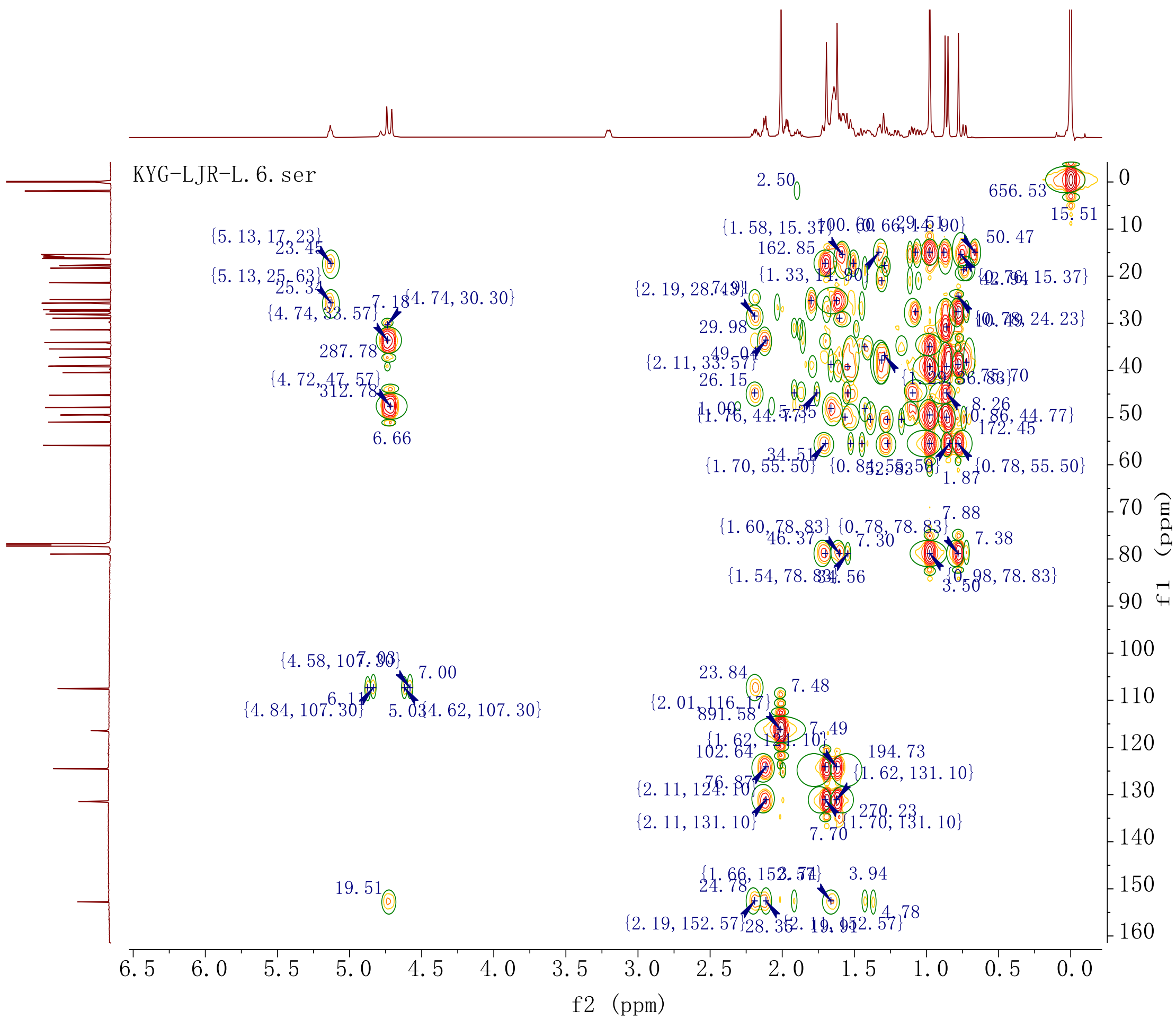

Supplementary Figure 5. HMBC spectrum of dammaradienol in $CDCl_3$.

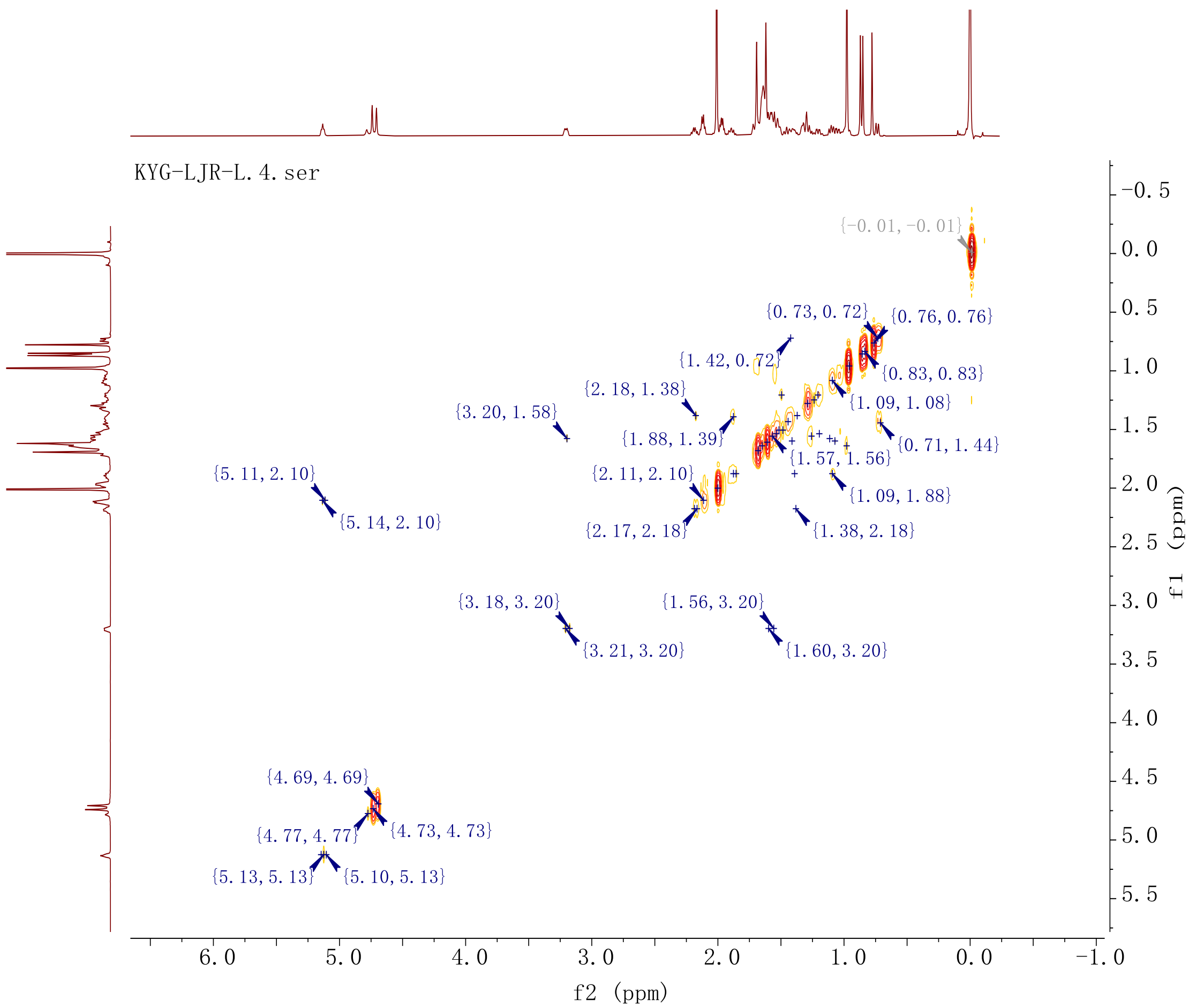

Supplementary Figure 6. ^{1}H-^{1}H COSY spectrum of dammaradienol in $CDCl_3$.

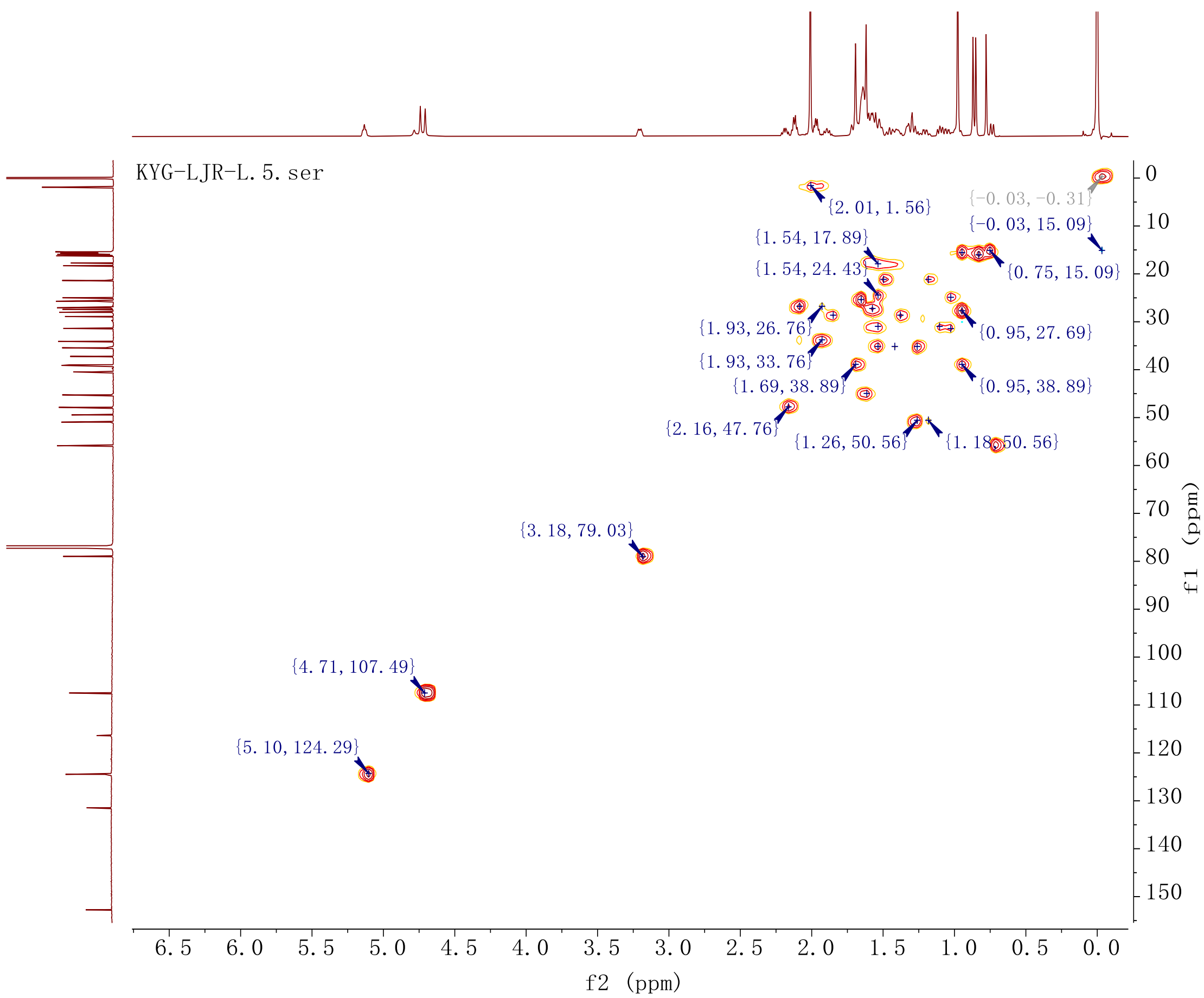

Supplementary Figure 7. HSQC spectrum of dammaradienol in $CDCl_3$.

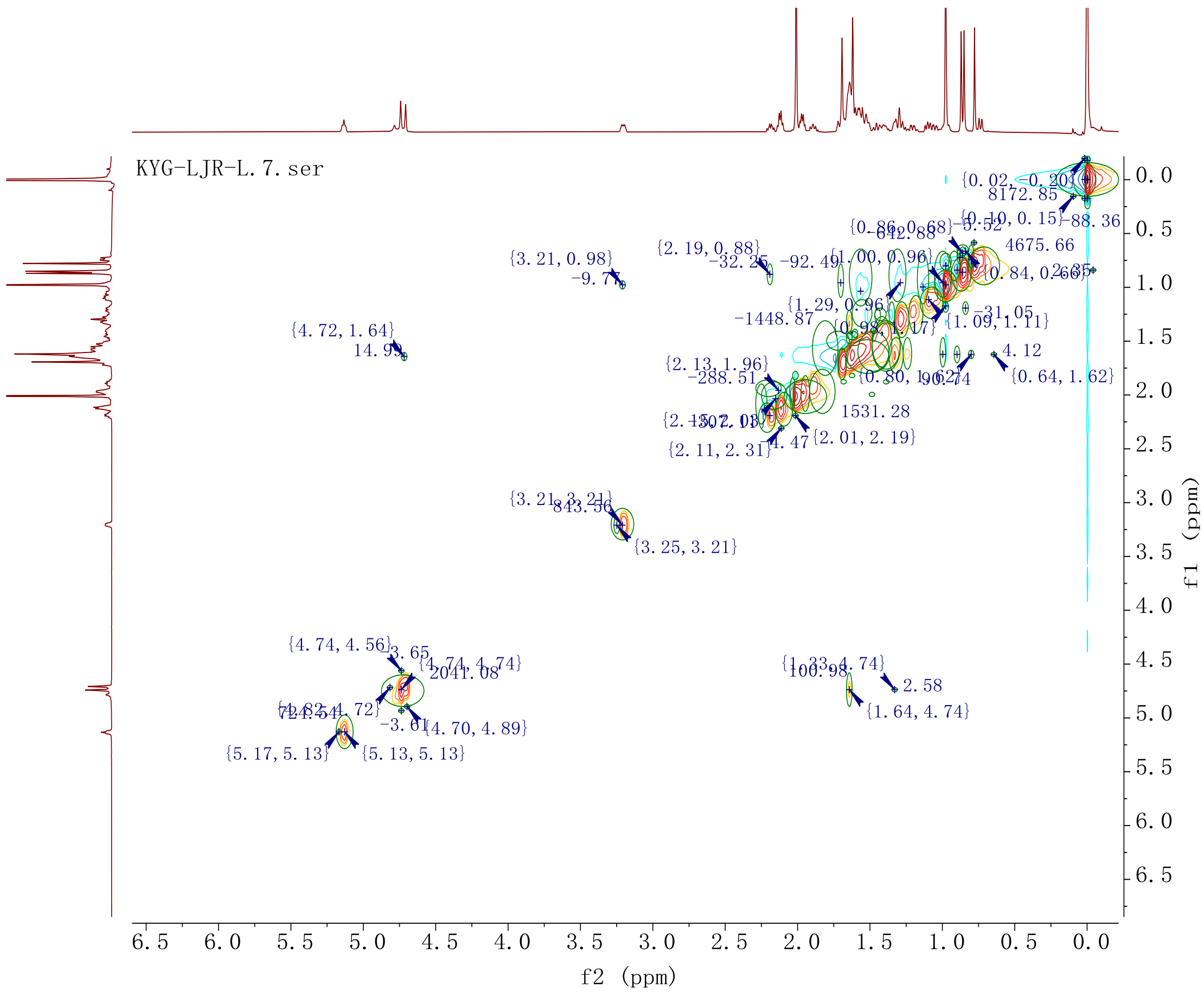

Supplementary Figure 8. NOESY spectrum of dammaradienol in $CDCl_3$.

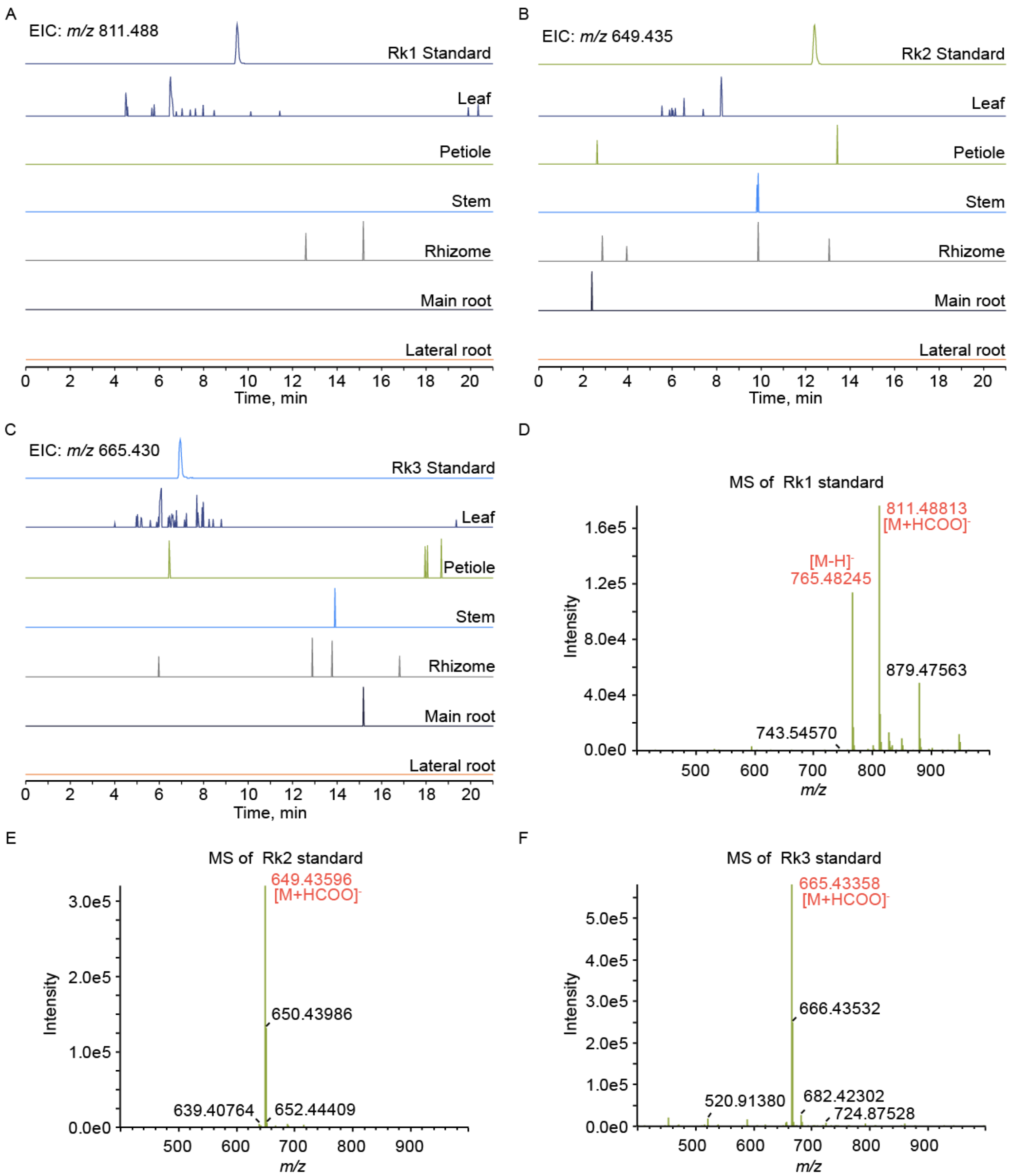

Supplementary Figure 9. Metabolite analysis of *O. elatus* by LC-MS. (A) Extracted ion chromatograms (EICs) of ginsenoside Rk1 (*m/z* 811.488) from LC-MS analysis of Rk1 standard and various *O. elatus* tissues (leaf, petiole, stem, rhizome, main root, and lateral root). The stacked chromatograms were normalized to a maximum relative intensity of 100%; (B) Extracted ion chromatograms (EICs) of ginsenoside Rk2 (*m/z* 649.435) from LC-MS analysis of Rk2 standard and six *O. elatus* tissues. The stacked chromatograms were normalized to a maximum relative intensity of 100%; (C) Extracted ion chromatograms (EICs) of ginsenoside Rk3 (*m/z* 665.430) from LC-MS analysis of Rk3 standard and six *O. elatus* tissues. The stacked chromatograms were normalized to a maximum relative intensity of 100%; (D) Mass spectra of ginsenoside Rk1 standards obtained via LC-MS analysis; (E) Mass spectra of ginsenoside Rk2 standards obtained via LC-MS analysis ; (F) Mass spectra of ginsenoside Rk3 standards obtained via LC-MS analysis.

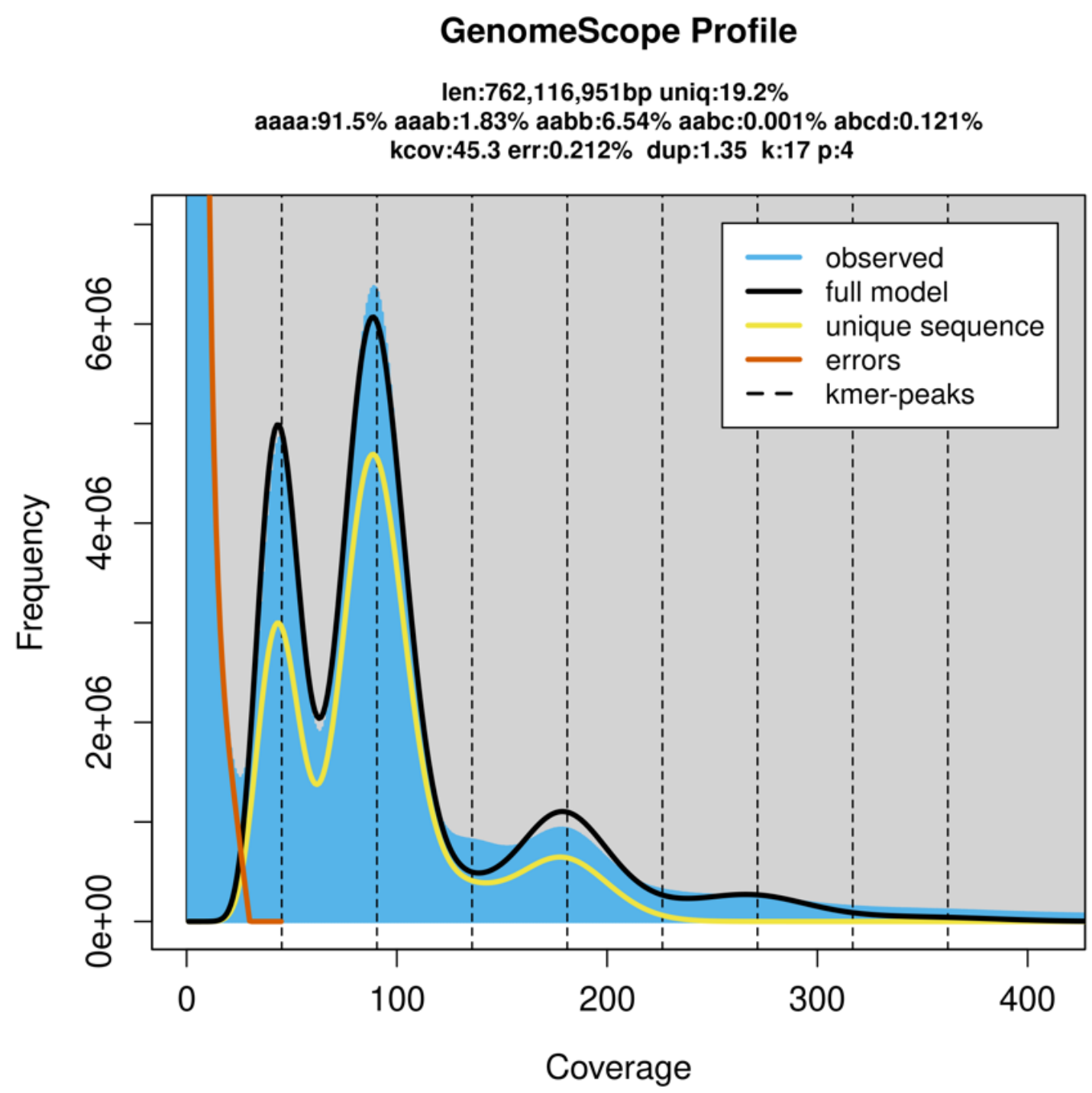

Supplementary Figure 10. *K*-mer coverage and frequency distribution in *O. elatus* based on 17-mer. The x-axis represents coverage, and the y-axis represents frequency. The blue shaded area indicates the observed coverage distribution. The black curve represents the fitted full model, the yellow curve denotes the distribution of unique sequences, and the orange curve corresponds to erroneous *k*-mers.

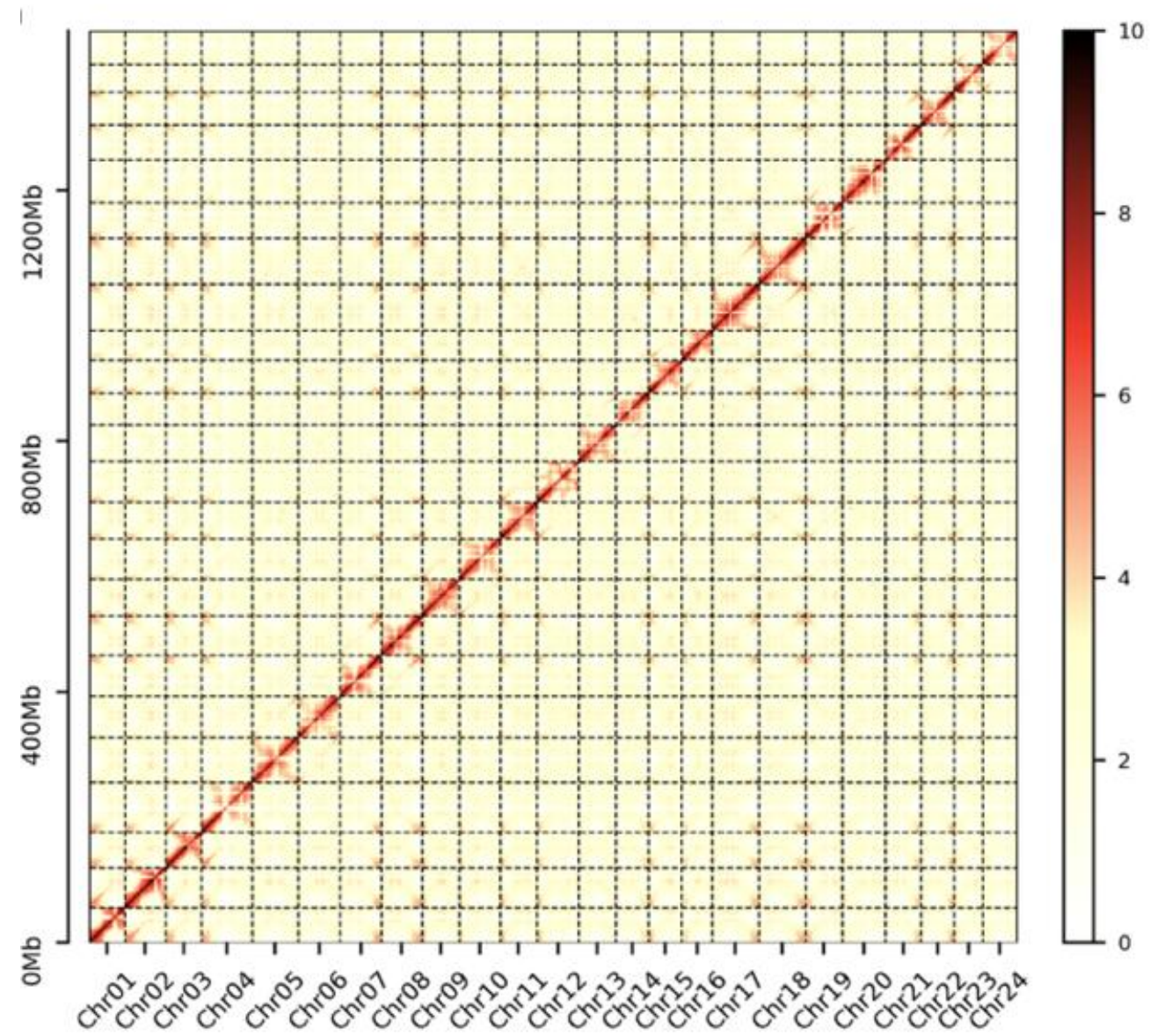

Supplementary Figure 11. Hi-C interaction signal intensity in *O. elatus*. The color gradient from yellow to red represents increasing interaction intensity, with red indicating stronger chromatin interactions.

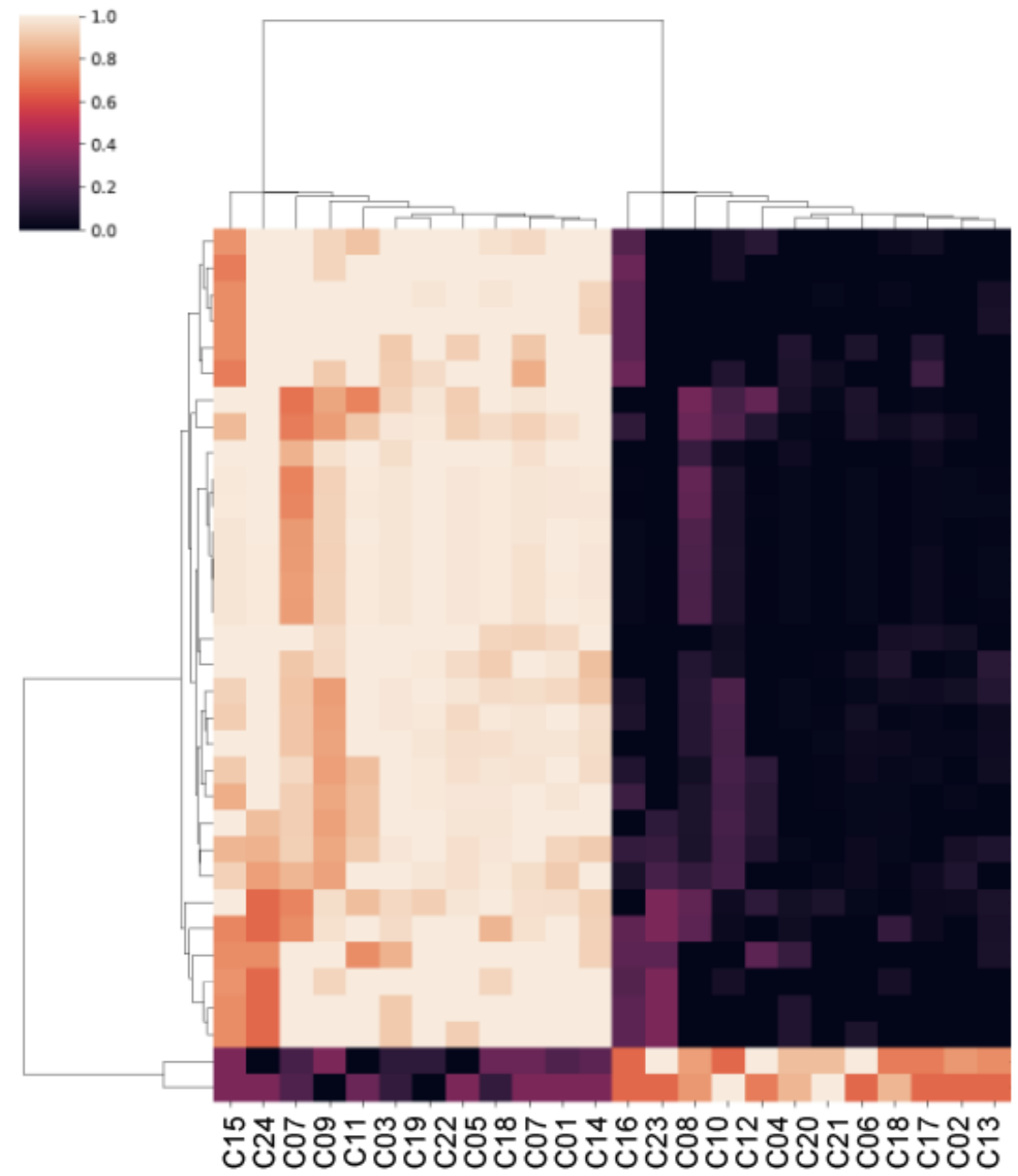

Supplementary Figure 12. Subgenome typing of the allopolyploid *O. elatus* genome. Distribution heatmap of 33 specific *K*-mers across the 24 chromosomes of *O. elatus*.

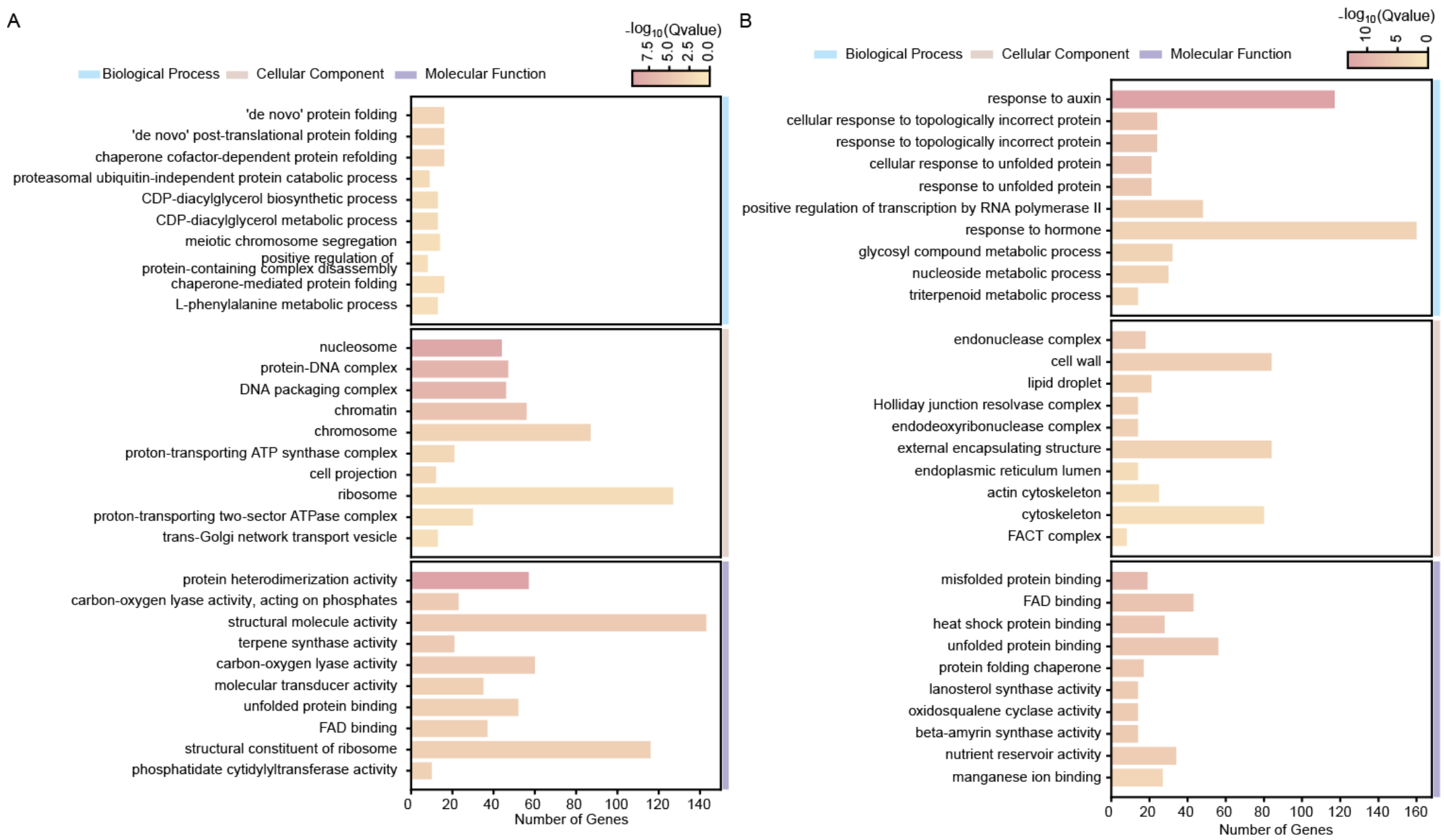

Supplementary Figure 13. GO enrichment analysis of expanded gene families in *O. elatus*. (A) GO enrichment analysis of expanded gene families in subgenome A of *O. elatus*. (B) GO enrichment analysis of expanded gene families in subgenome B of *O. elatus*. Source data are provided as a Source Data file.

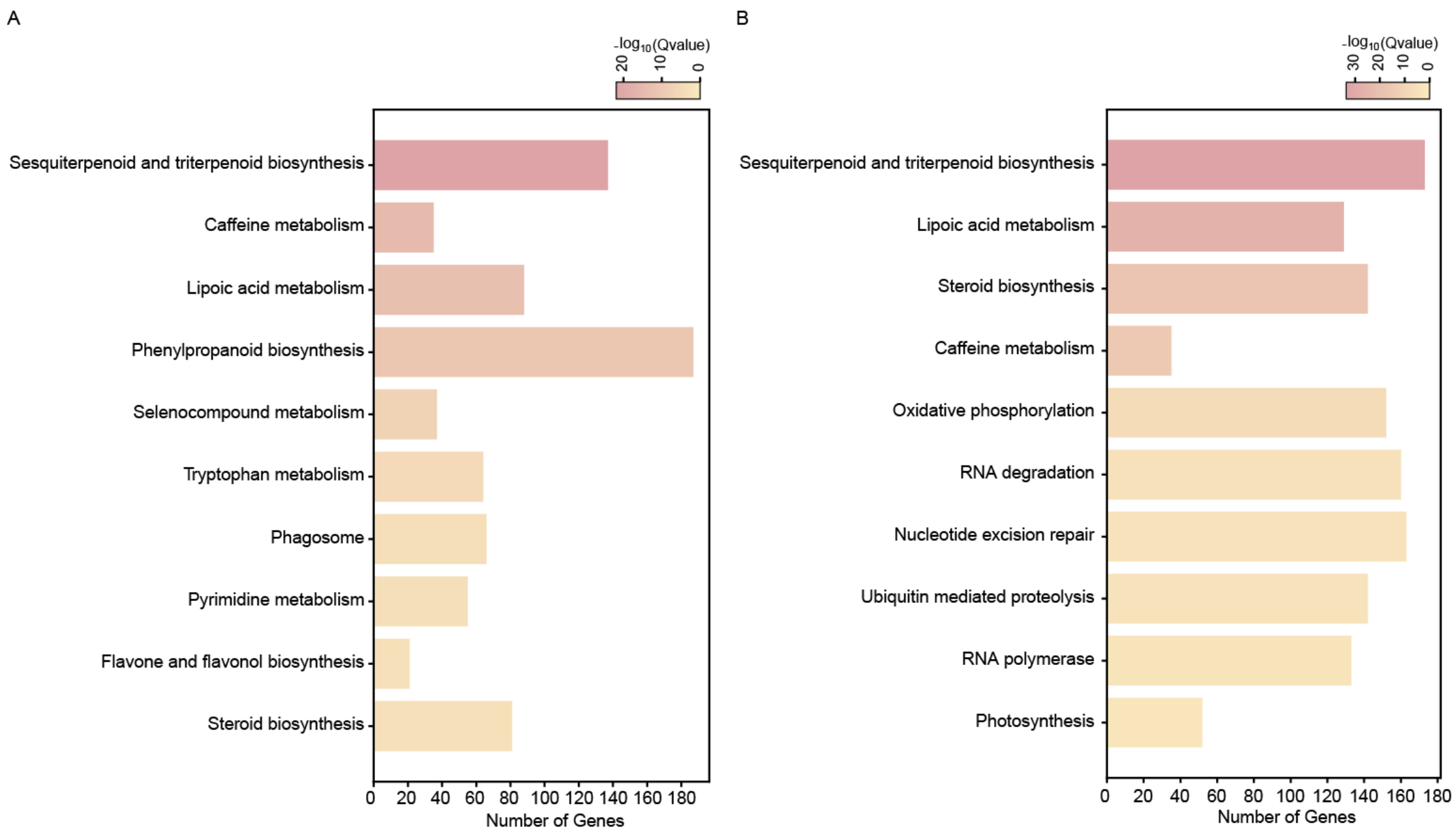

Supplementary Figure 14. KEGG enrichment analysis for expanded gene families in *O. elatus.* (A) KEGG enrichment analysis of expanded gene families in subgenome A of *O. elatus*. (B) KEGG enrichment analysis of expanded gene families in subgenome B of *O. elatus*. Source data are provided as a Source Data file.

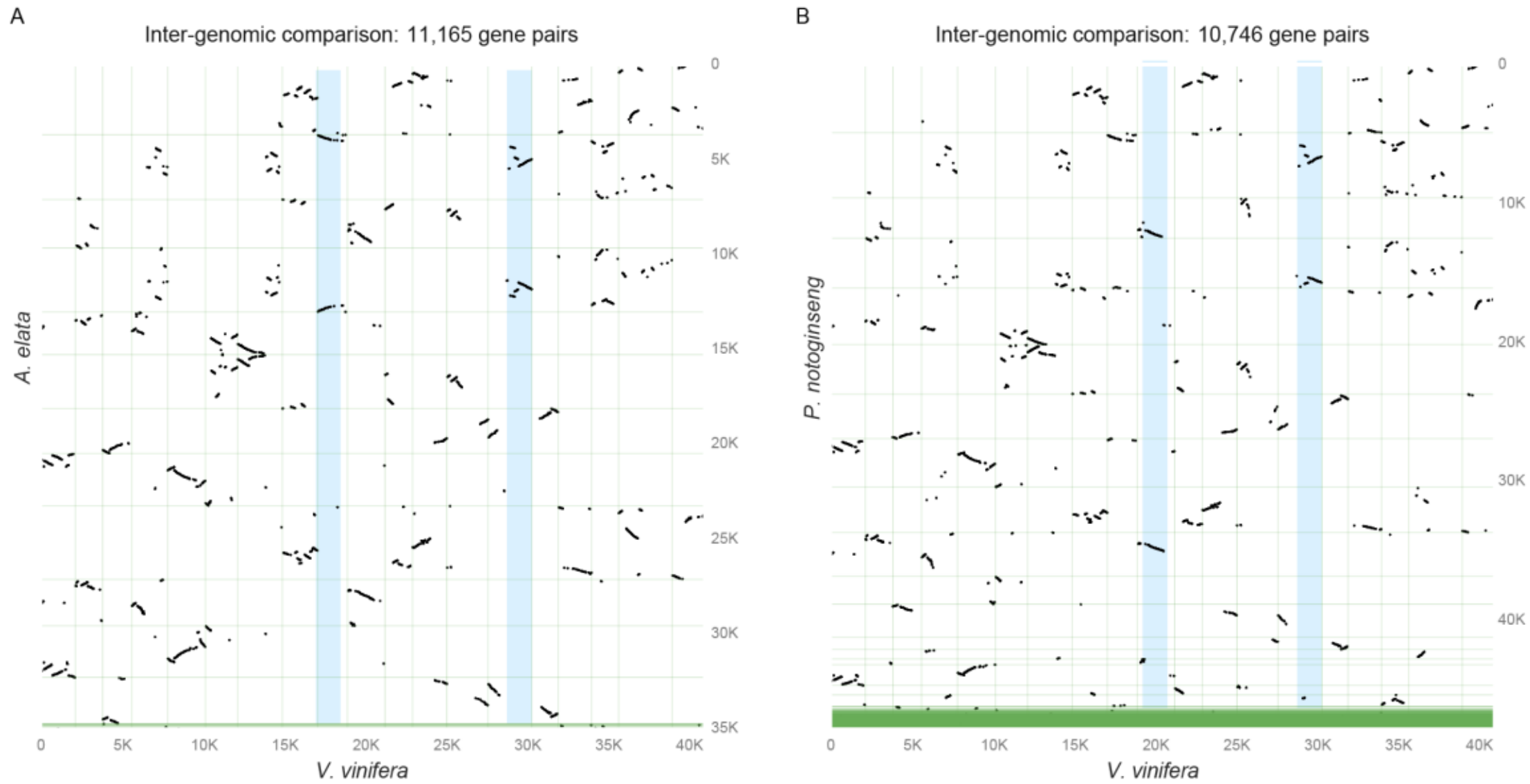

Supplementary Figure 15. Gene synteny dot plots between *V. vinifera* and diploid species of the Araliaceae, including *A. elata* and *P. notoginseng*. (A) Gene synteny dot plots between *V. vinifera* and *A. elata*; (B) Gene synteny dot plots between *V. vinifera* and *P. notoginseng*.

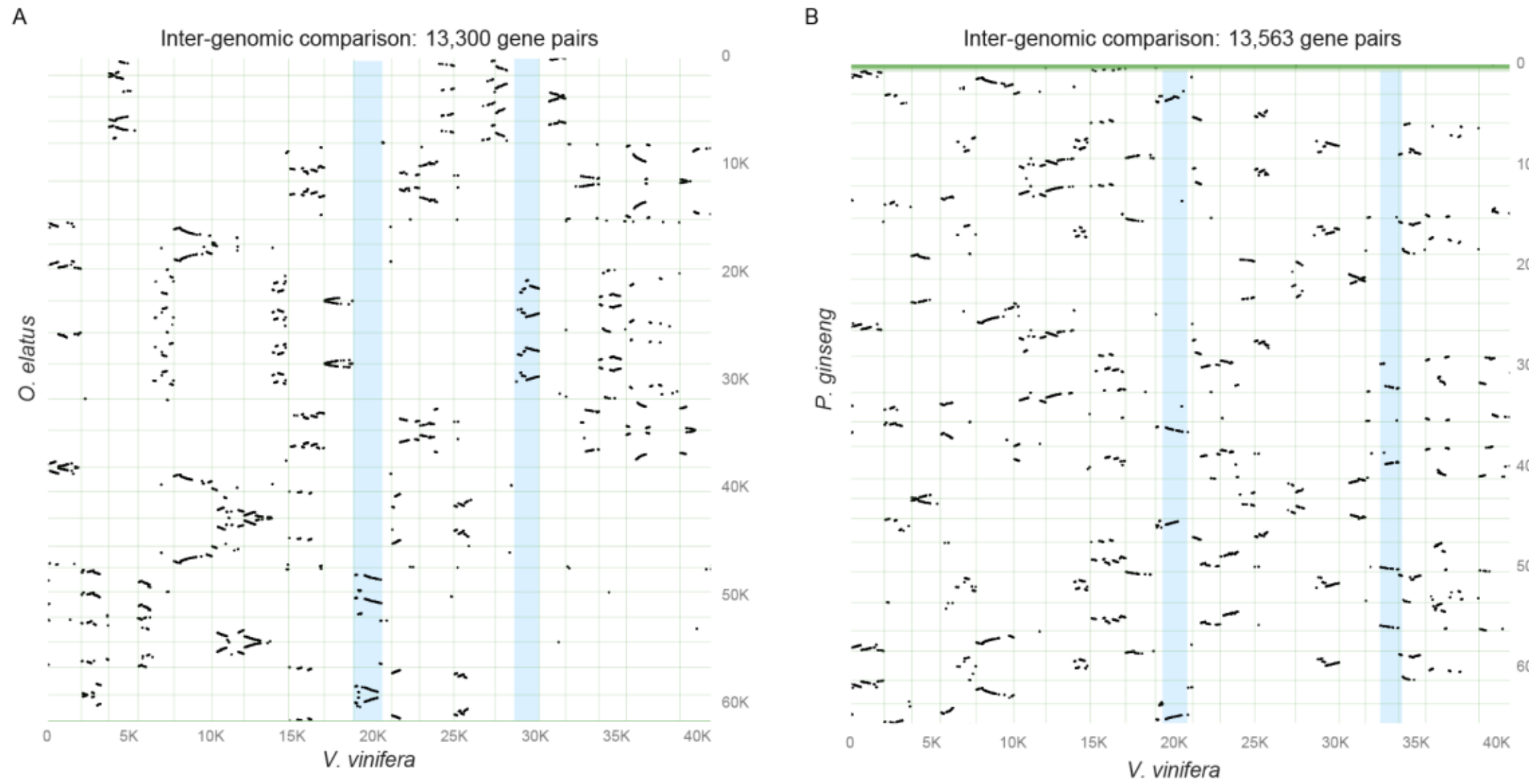

Supplementary Figure 16. Gene synteny dot plots between *V. vinifera* and tetraploid species of the Araliaceae, including *O. elatus* and *P. ginseng*. (A) Gene synteny dot plots between *V. vinifera* and *O. elatus*; (B) Gene synteny dot plots between *V. vinifera* and *P. ginseng*.

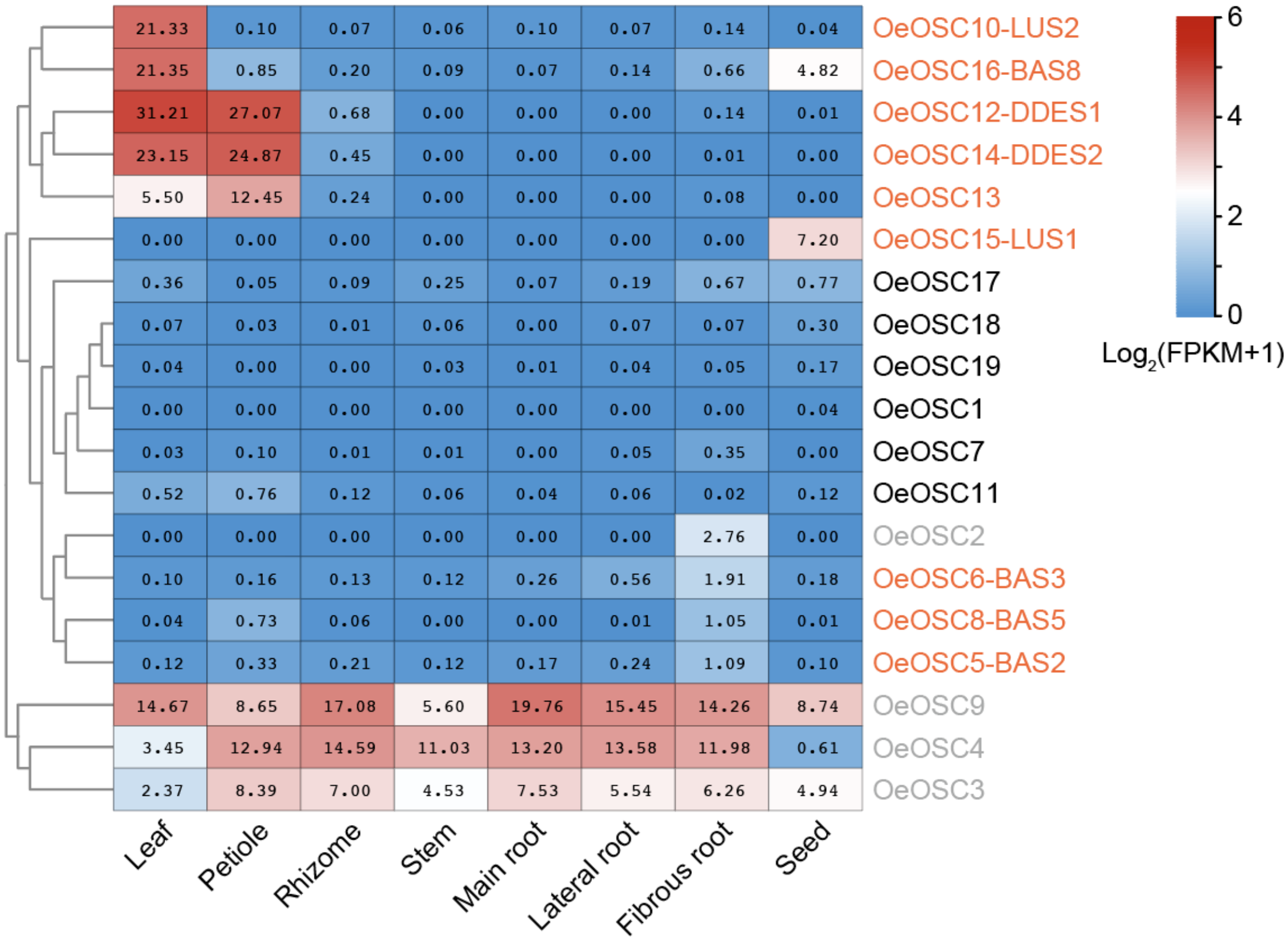

Supplementary Figure 17. Expression profiles of *OeOSC* genes of *O. elatus* in different tissues. The expression values are shown as FPKM (fragments per kilobase of transcript per million mapped reads) and represent the mean of three biological replicates. Genes encoding cycloartenol synthase (CAS) and lanosterol synthase (LAS), involved in sterol biosynthesis, are colored in grey letter. Candidate genes with FPKM > 1 that were selected for functional validation are highlighted in orange color.

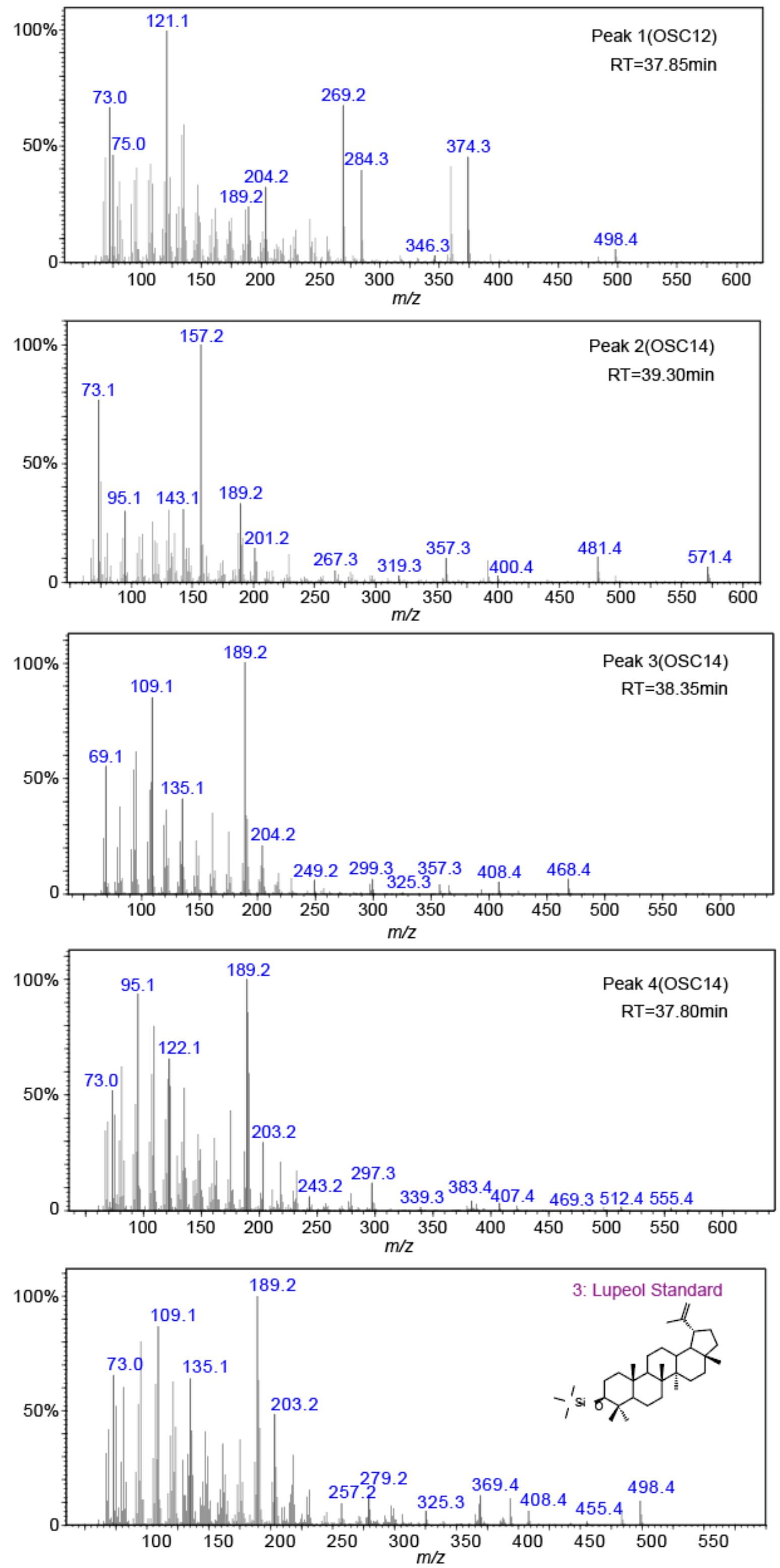

Supplementary Figure 18. Mass spectrometry fragmentation profiles of OeOSC-derived unknown products and lupeol standard.

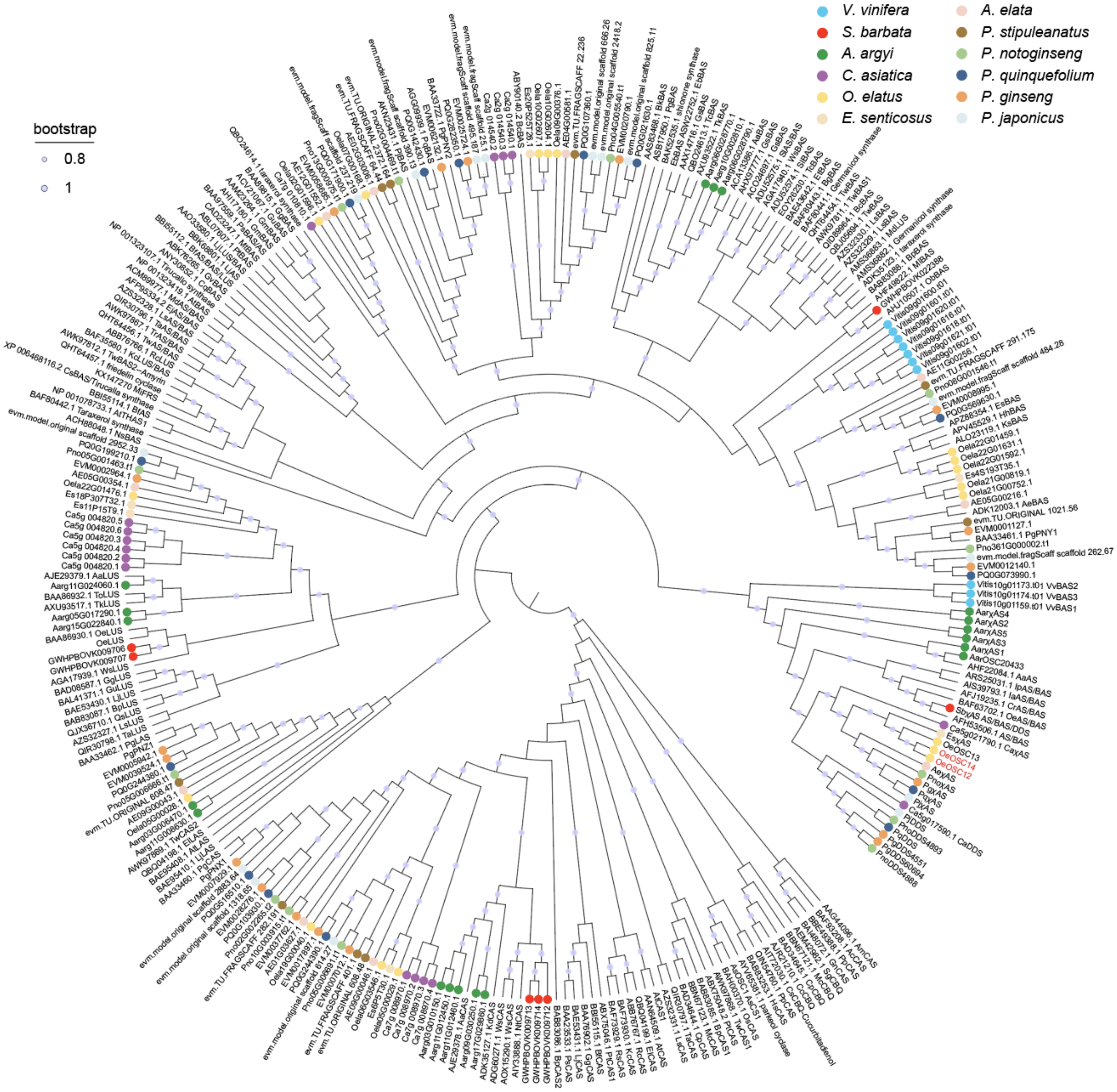

Supplementary Figure 19. Maximum-likelihood tree of plant oxidosqualene cyclases (OSCs). Filled purple circles indicate bootstrap support values for nodes greater than 0.8. Circles of different colors represent distinct species: *Vitis vinifera*, *Scutellaria barbata*, *Artemisia argyi*, *Centella asiatica* and eight species from Araliaceae, namely *Oplopanax elatus*, *Eleutherococcus senticosus*, *Aralia elata*, *Panax stipuleanatus*, *Panax notoginseng*, *Panax quinquefolium*, *Panax ginseng*, and *Panax japonicus*. Source data are provided as a Source Data file.

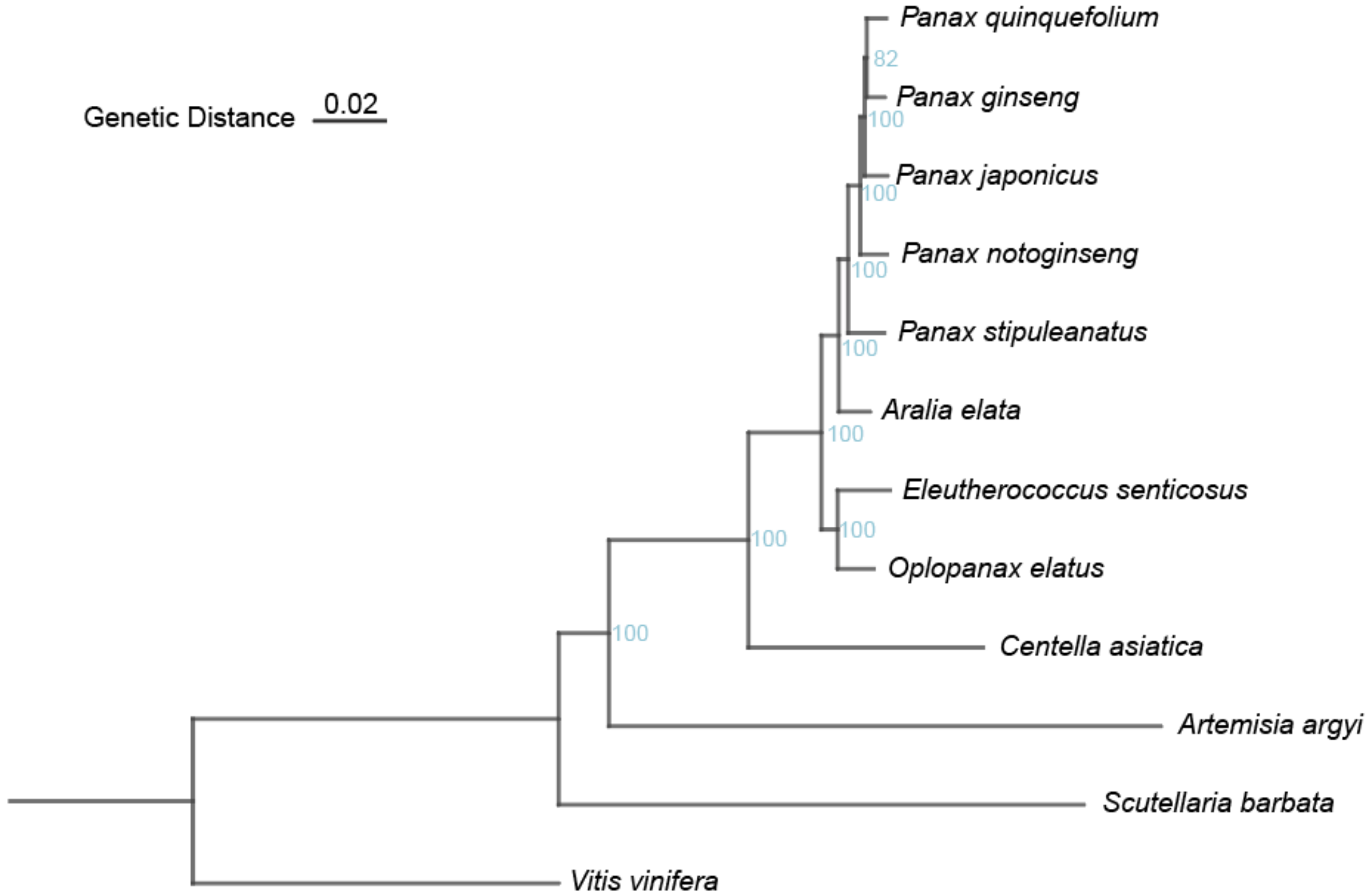

Supplementary Figure 20. The phylogenetic analysis based on single-copy orthologous genes among 12 selected species. The analysis includes *Panax quinquefolium*, *Panax ginseng*, *Panax japonicus*, *Panax notoginseng*, *Eleutherococcus senticosus*, *Oplopanax elatus*, *Artemisia argyi*, *Centella asiatica*, *Scutellaria barbata*, *Vitis vinifera*, *Aralia elata and Panax stipuleanatus*. Bootstrap values are shown on the internal nodes.

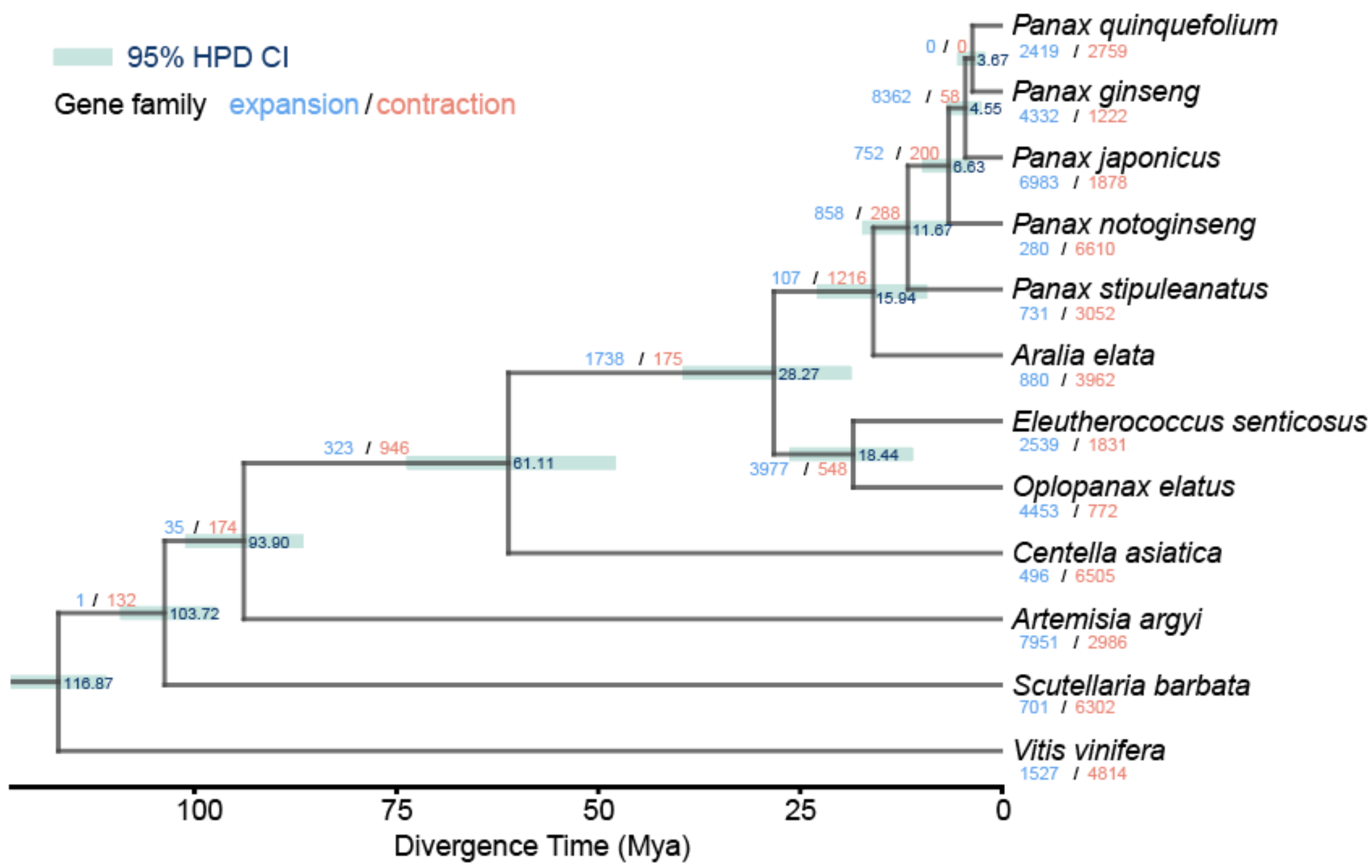

Supplementary Figure 21. The evolutionary analysis of 12 species. The analysis inlcudes *Panax quinquefolium*, *Panax ginseng*, *Panax japonicus*, *Panax notoginseng*, *Panax stipuleanatus*, *Aralia elata*, *Eleutherococcus senticosus*, *Oplopanax elatus*, *Artemisia argyi*, *Centella asiatica*, *Scutellaria barbata* and *Vitis vinifera*. Divergence times were estimated by MCMCTree, and the 95% confidence intervals of divergence times are shown on the internal nodes. The numbers of gene family expansions are indicated in blue, and gene family contractions are indicated in orange.

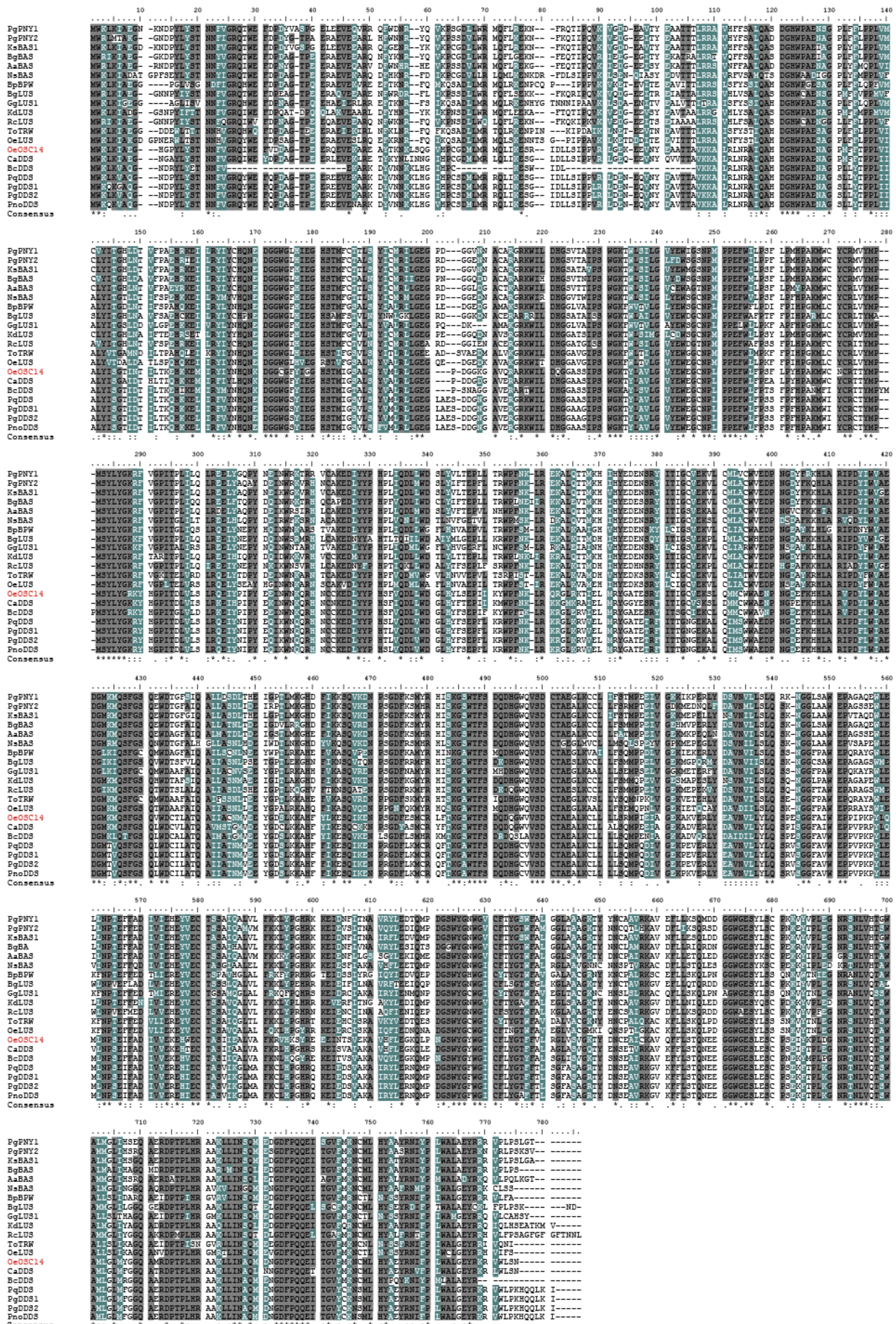

Supplementary Figure 22. Multiple amino acid sequence alignment of oxidosqualene cyclases (OSCs). A multiple sequence alignment was performed among OeOSC14 and other OSC subfamily genes from various species.

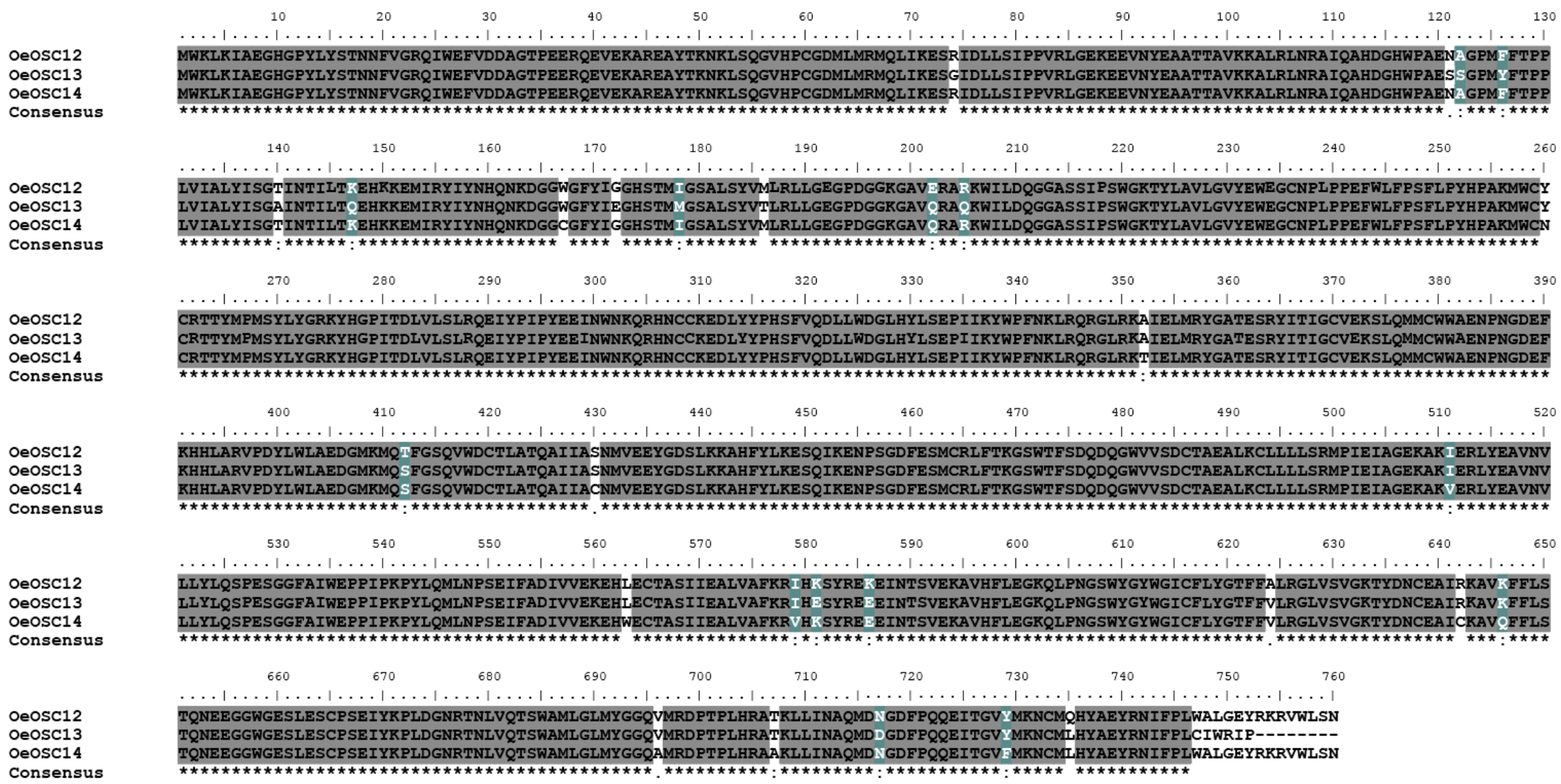

Supplementary Figure 23. A pairwise sequence alignment was conducted between OeOSC12, OeOSC13, and OeOSC14 utilizing the Needleman-Wunsch algorithm.

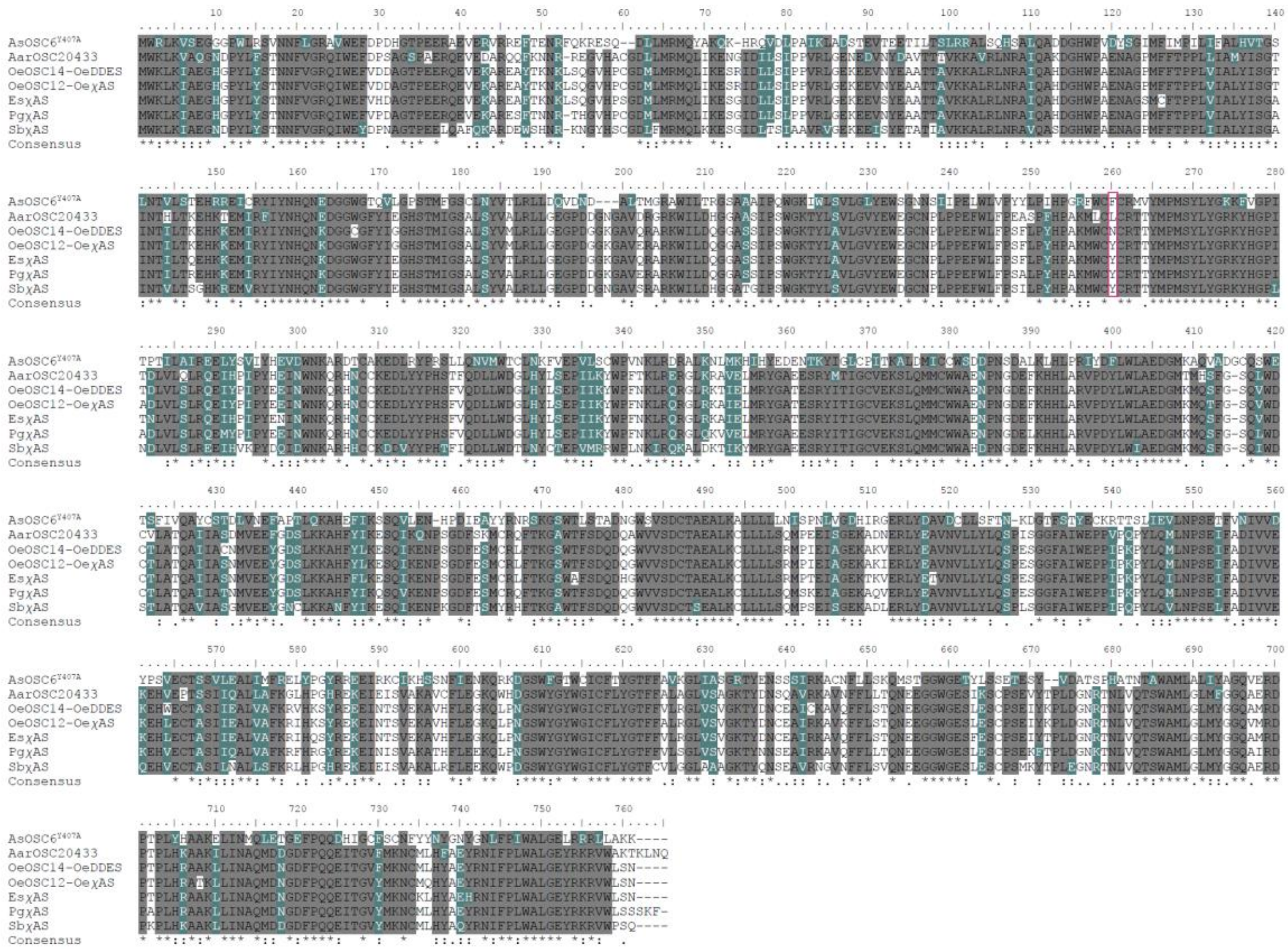

Supplementary Figure 24. A multiple amino acid sequence alignment was conducted among mTTSs utilizing the Needleman-Wunsch algorithm.

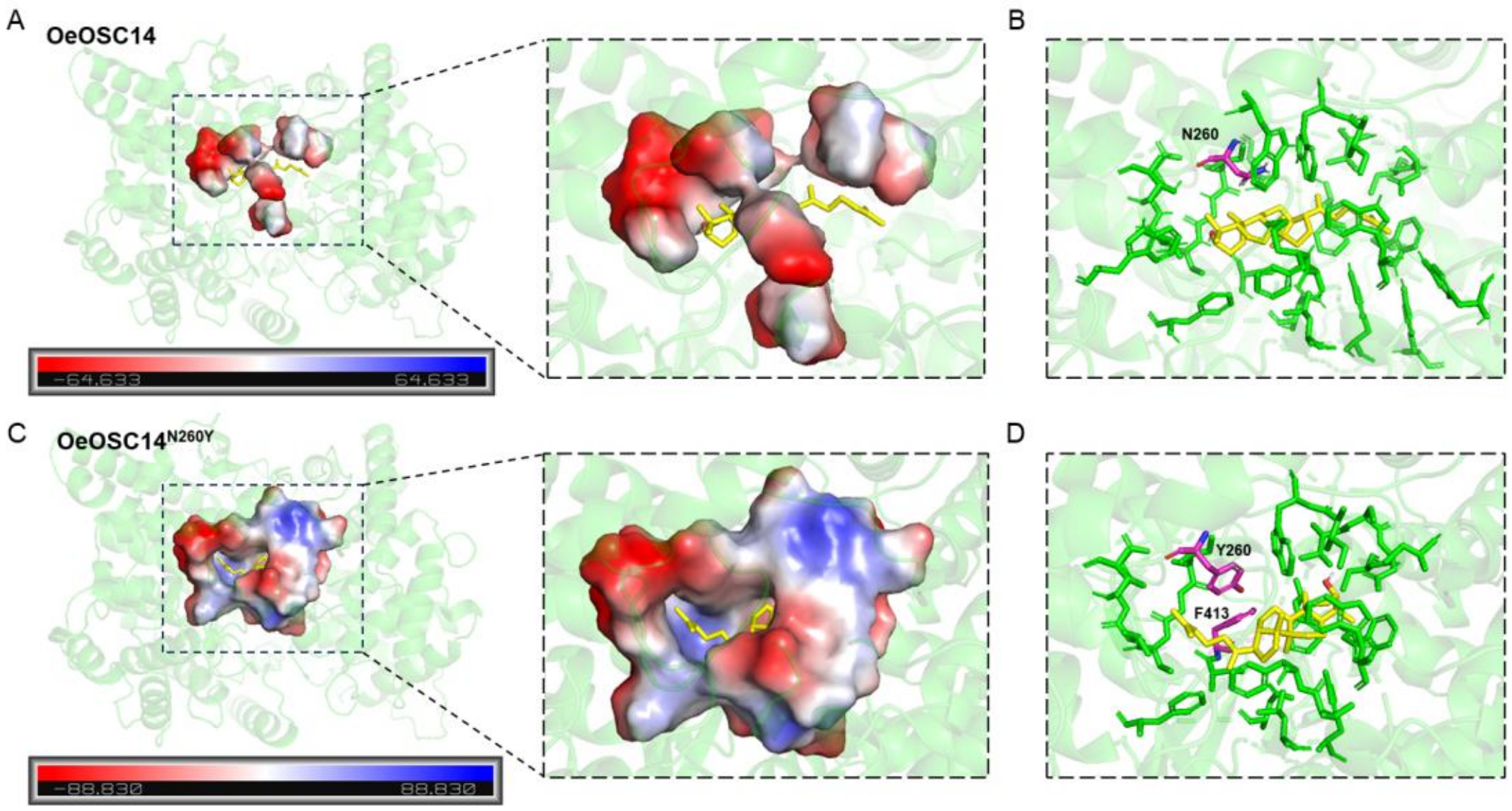

Supplementary Figure 25. Electrostatic surface potential and local active-pocket residue configuration of OeOSC14 and OeOSC14^{N260Y} in complex with dammaradienol. Surface electrostatic potentials were calculated using APBS and mapped onto the molecular surface, with red, blue, and white indicating negative, positive, and neutral potentials, respectively. (A) Electrostatic surface potential of OeOSC14 -dammaradienol complex, with dammaradienol shown as yellow sticks; the right panel shows a magnified view of the binding pocket region; (B) Amino acid residues within 5 Å of dammaradienol in OeOSC14, with dammaradienol shown as yellow sticks and N260 shown as purple sticks; (C) Electrostatic surface potential of the OeOSC14^{N260Y}-dammaradienol complex; the right panel shows a magnified view of the binding pocket region; (D) Amino acid residues within 5 Å of dammaradienol in OeOSC14^{N260Y}, with dammaradienol shown as yellow sticks, and Y260 together with F413 shown as purple sticks.

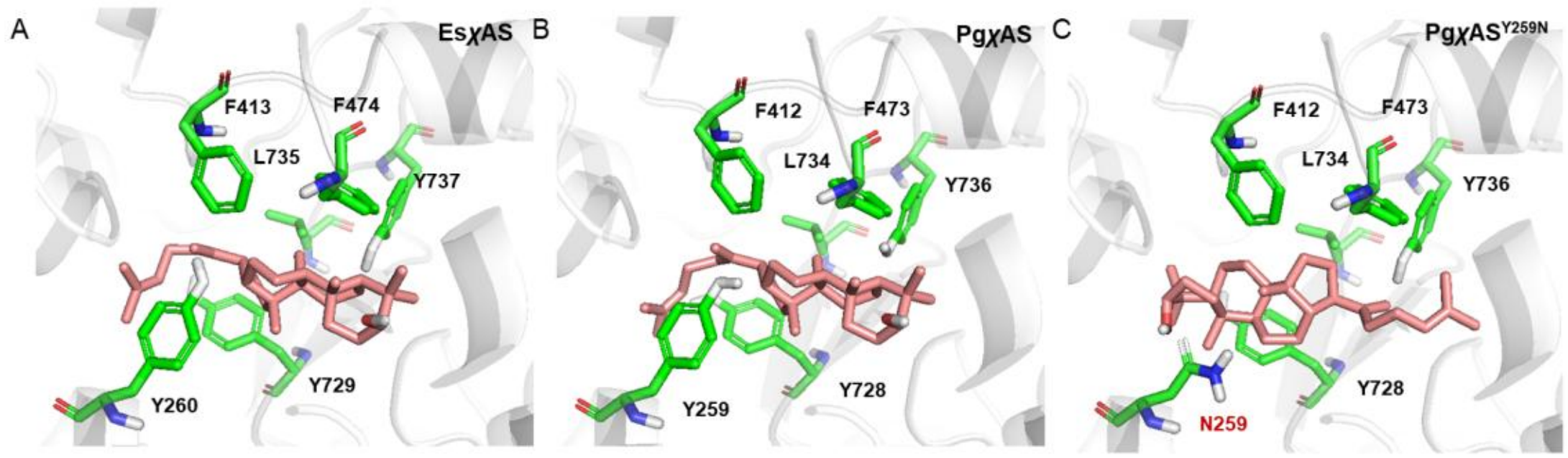

Supplementary Figure 26. Molecular docking of mTTS and its variants with dammaradienol. Molecular docking models of the mTTS – dammaradienol complexes, illustrating the spatial interactions between dammaradienol and key residues within the mTTS active pocket. Dammaradienol is depicted as pink sticks. The labeled amino acid residues are located within 5 Å of the ligand. (A) Molecular docking models of the EsχAS – dammaradienol complexes; (B) Molecular docking models of the PgχAS – dammaradienol complexes; (C) Molecular docking models of the PgχAS^{Y259N}– dammaradienol complexes.

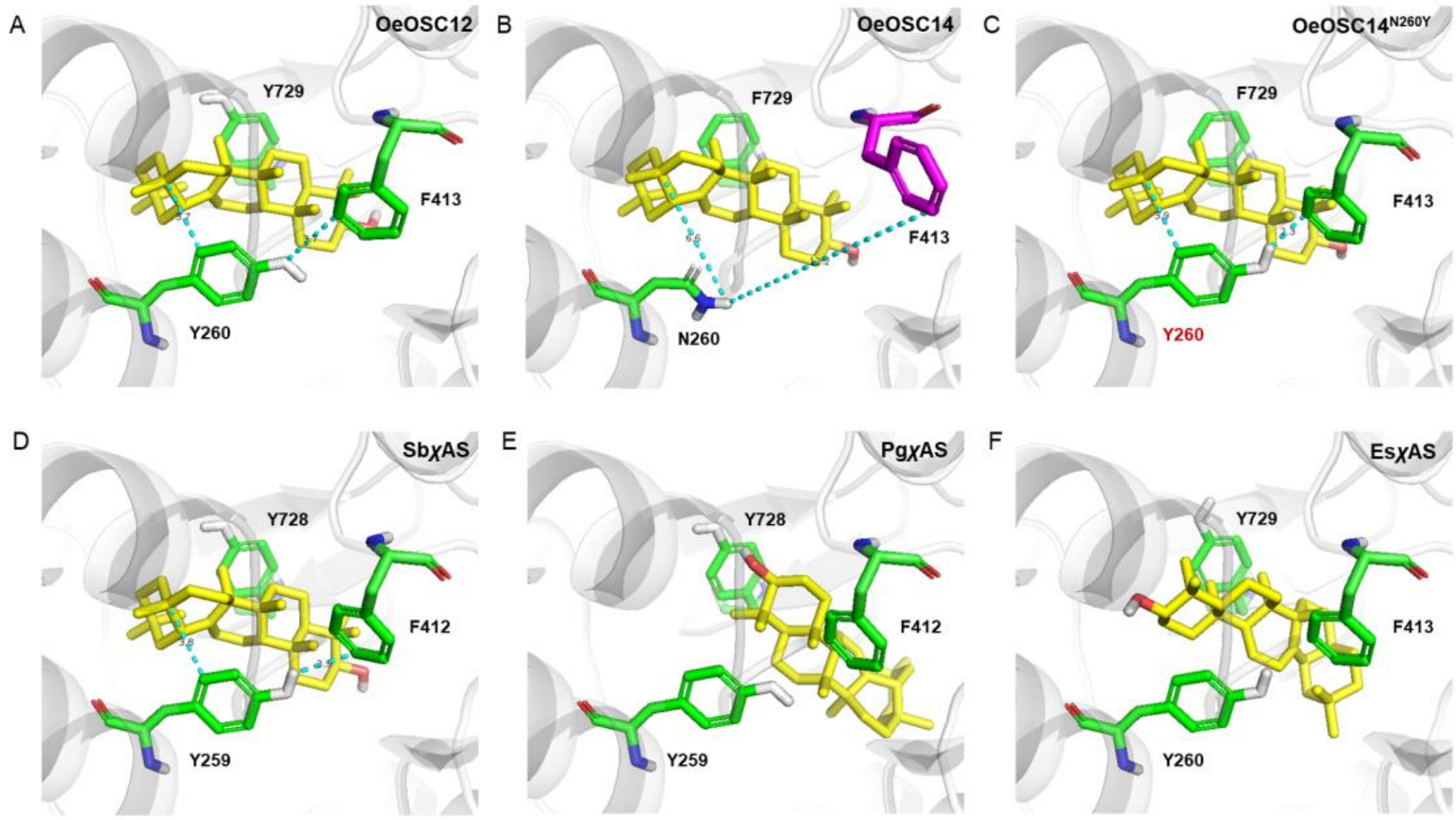

Supplementary Figure 27. Molecular docking of mTTS with β-amyrin. Molecular docking models of mTTS – β-amyrin complexes, illustrating the spatial interactions between β-amyrin and key residues within the mTTS active pocket. *β*-amyrin is depicted as yellow sticks. Green and magenta labels indicate amino acid residues located within and beyond 5 Å of the ligand, respectively. Distances are indicated by cyan dashed lines and labeled with numbers. (A) Molecular docking models of the OeOSC12 – β-amyrin complexes; (B) Molecular docking models of the OeOSC14 – β-amyrin complexes; (C) Molecular docking models of the OeOSC14^{N260Y} – β-amyrin complexes; (D) Molecular docking models of the SbχAS – β-amyrin complexes; (E) Molecular docking models of the PgχAS – β-amyrin complexes; (F) Molecular docking models of the EsχAS – β-amyrin complexes;

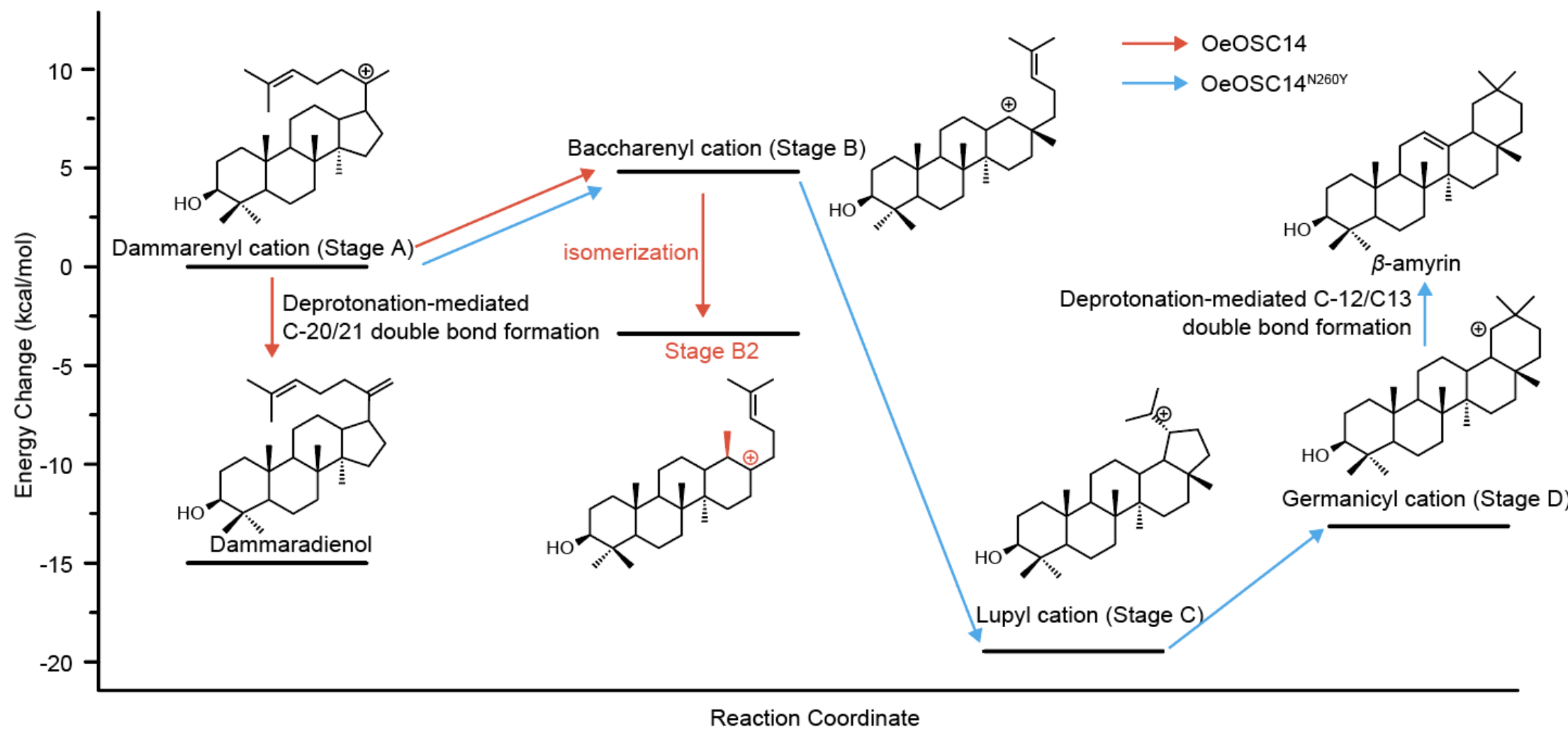

Supplementary Figure 28. Quantum-chemical calculations for triterpene products generated by the catalytic action of OeOSC14 and OeOSC14^{N260Y}. Proposed reaction pathways from the dammarenyl cation are shown with relative Gibbs free energies (ΔG, kcal/mol) for OeOSC14 (red) and OeOSC14^{N260Y} (blue).

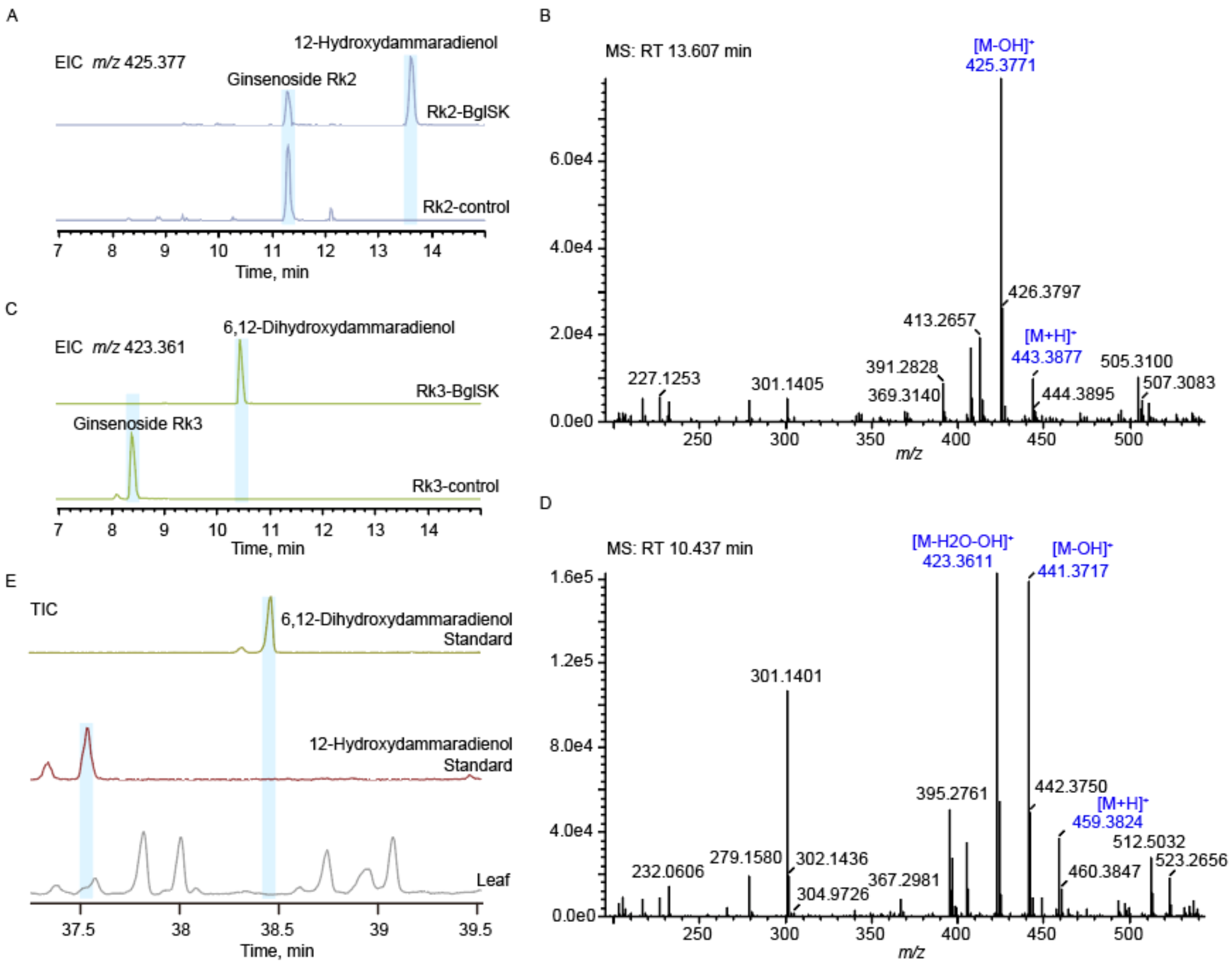

Supplementary Figure 29. Preparation of 12-hydroxydammaradienol and 6,12-dihydroxydammaradienol reference standards by glycosidase-catalyzed hydrolysis for the analysis of *O. elatus* leaf extracts. (A) Extracted ion chromatograms (EICs) from LC–MS analysis of 12-hydroxydammaradienol produced by BglSK-catalyzed hydrolysis of ginsenoside Rk2. The extracted ion was set at *m/z* 425.377 for both ginsenoside Rk2 and 12-hydroxydammaradienol. (B) MS spectrum of 12-Hydroxydammaradienol obtained by LC–MS. (C) Extracted ion chromatograms (EICs) from LC–MS analysis of 6,12-dihydroxydammaradienol produced by BglSK-catalyzed hydrolysis of ginsenoside Rk3. The extracted ion was set at *m/z* 423.361 for both ginsenoside Rk3 and 6,12-dihydroxydammaradienol. (D) MS spectrum of 6,12-Dihydroxydammaradienol obtained by LC–MS. (E) Total ion chromatograms by GC-MS analysis of leaf extracts of *O. elatus* and the reference standards 12-hydroxydammaradienol and 6,12-dihydroxydammaradienol.

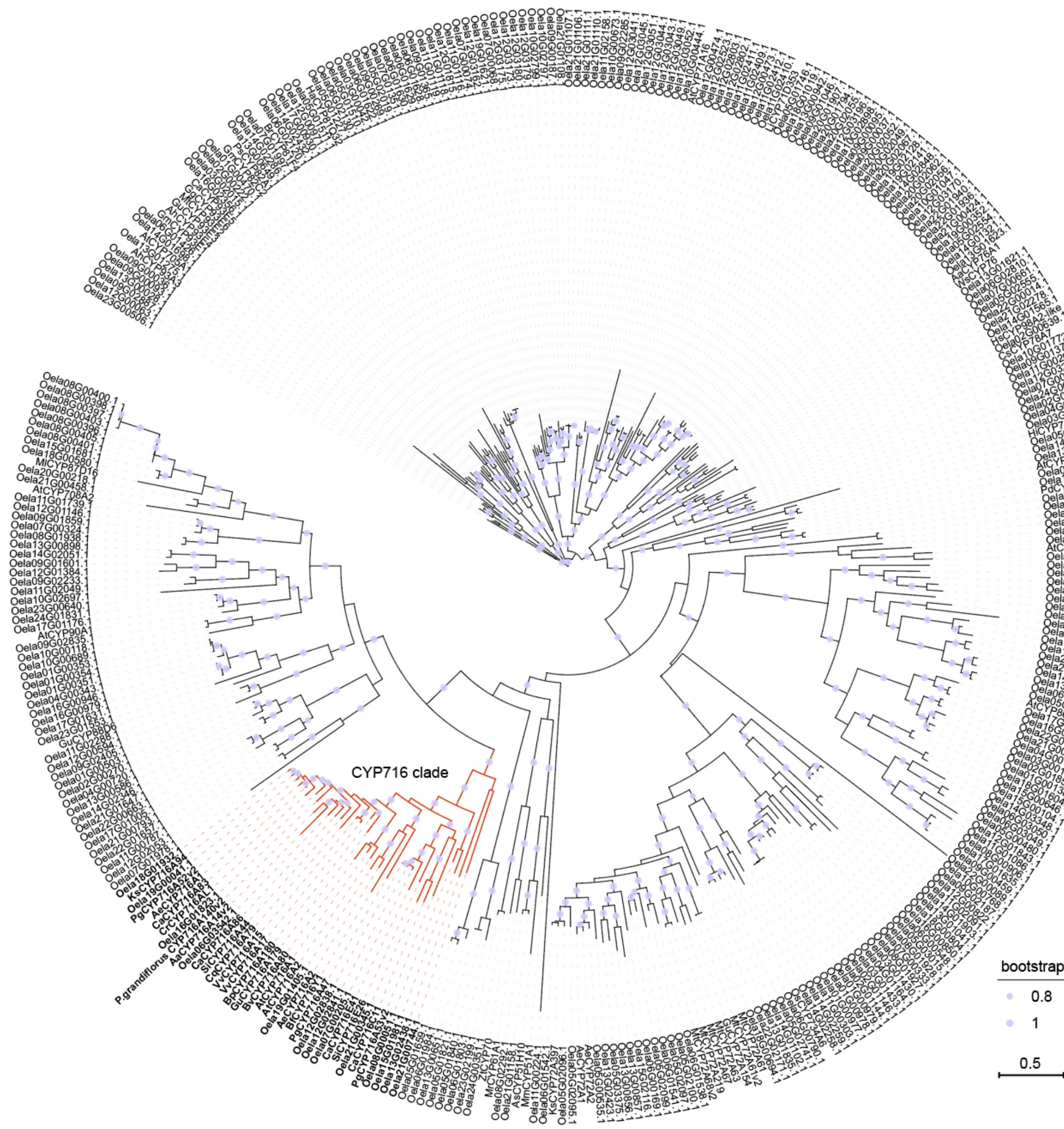

Supplementary Figure 30. Maximum-likelihood tree of cytochrome P450s (CYPs) in *O. elatus.* Filled purple circles indicate bootstrap support values for nodes greater than 0.8. Genes belonging to the CYP716 subfamilies, involved in triterpenoid biosynthesis, are highlighted in red. Source data are provided as a Source Data file.

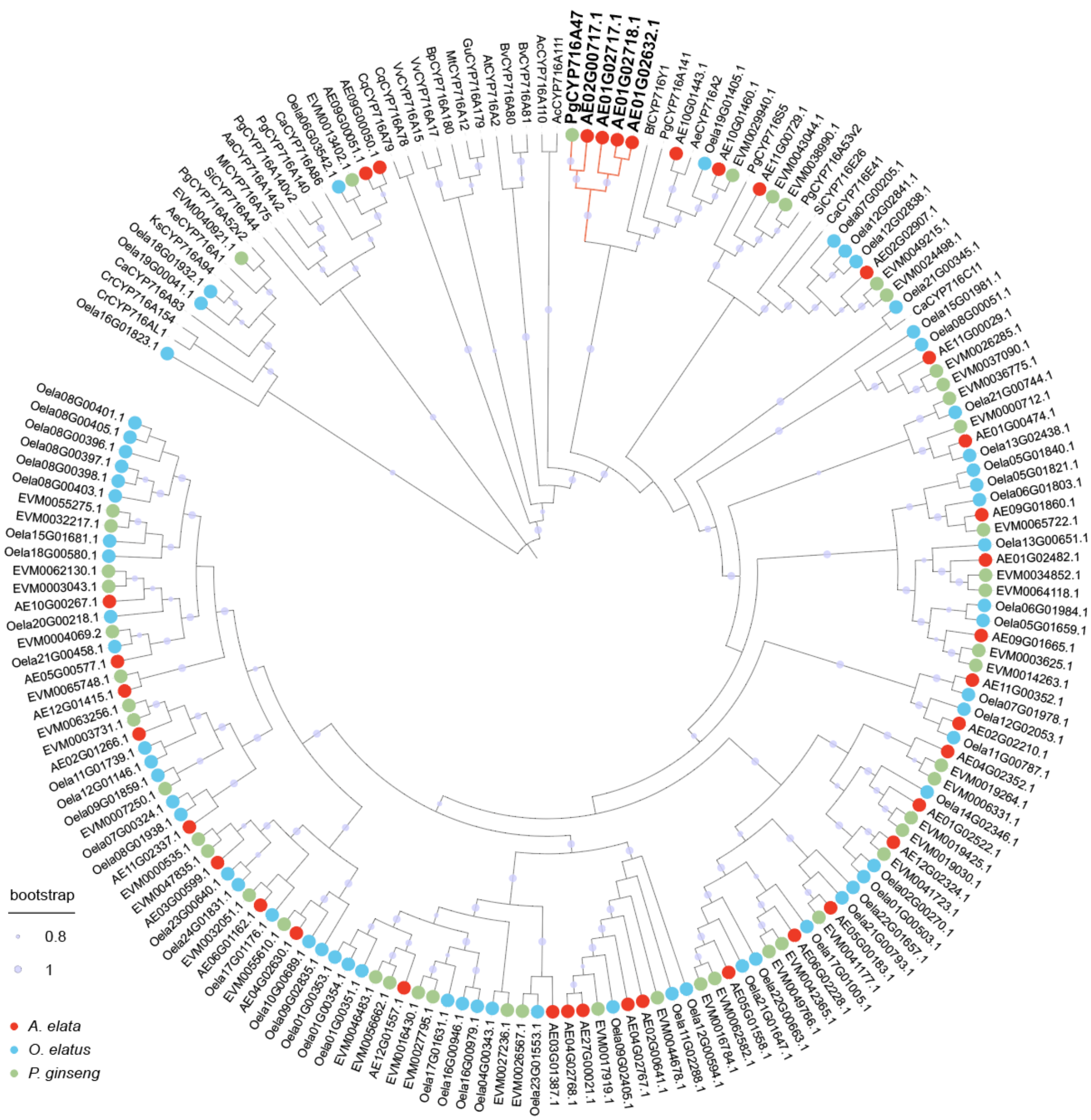

Supplementary Figure 31. Maximum-likelihood tree of CYP716 subfamily genes among *A. elata*, *O. elatus*, and *P. ginseng*. Filled purple circles indicate bootstrap support values for nodes greater than 0.8. Circles of different colors represent different species, including *A. elata*, *O. elatus*, and *P. ginseng*. Source data are provided as a Source Data file.

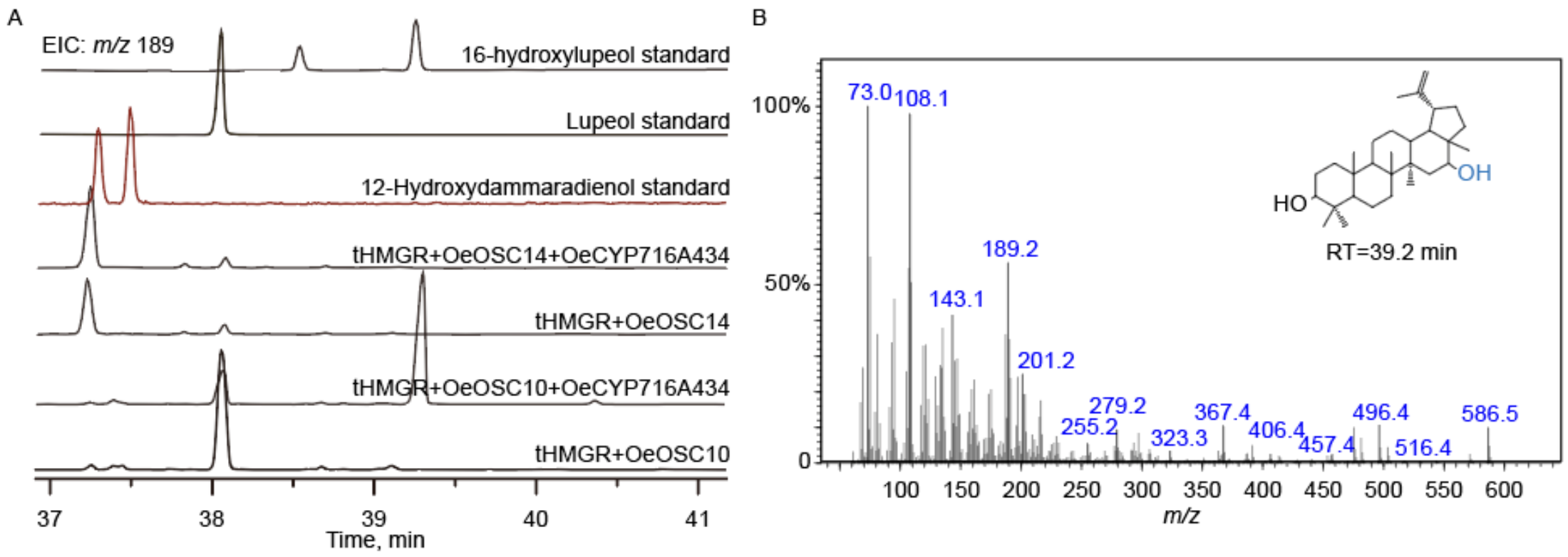

Supplementary Figure 32. Gene function validation of *OeCYP716A434* in *O. elatus.* (A) GC–MS extracted-ion chromatograms (*m*/*z* 189) of leaf extracts from *Nicotiana benthamiana* transiently co-expressing *tHMGR,* OeOSC10/OeOSC14 and *OeCYP716A434* genes. (B) Mass spectrometry fragmentation profiles of OeCYP-derived triterpene products.

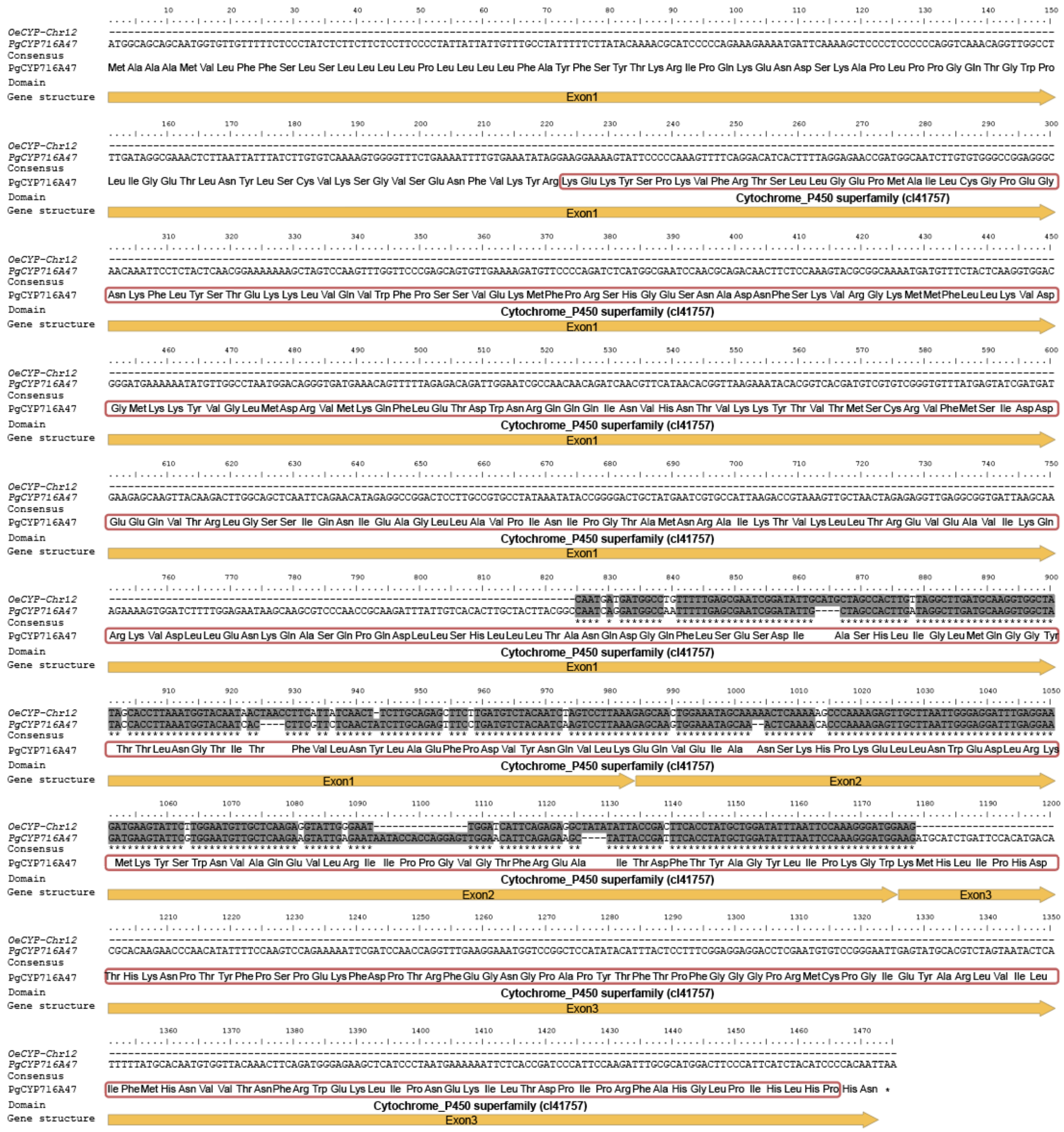

Supplementary Figure 33. Sequence alignment of *PgCYP716A47* from *P. ginseng* and its ortholog *OeCYP716* gene residue from chromosome 12 of *O. elatus.* Asterisk (*) indicates amino acids identical to consensus amino acid sequence and dashes indicate gaps included to obtain the best alignment. Shaded regions indicate matched areas in the sequence alignment.

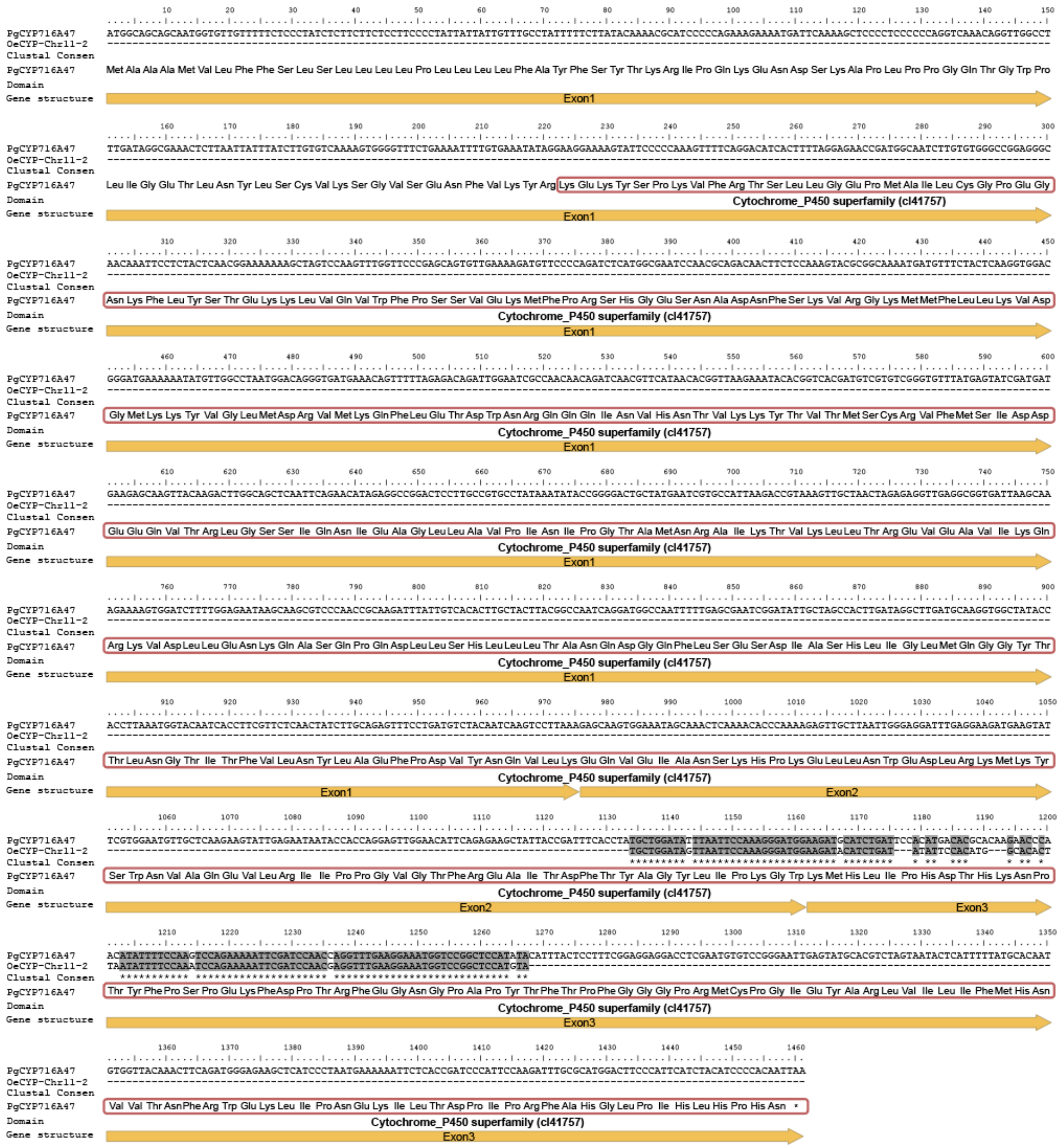

Supplementary Figure 34. Sequence alignment of *PgCYP716A47* from *P. ginseng* and its ortholog *OeCYP716* gene residue from chromosome 11 of *O. elatus.* Asterisk (*) indicates amino acids identical to consensus amino acid sequence and dashes indicate gaps included to obtain the best alignment. Shaded regions indicate matched areas in the sequence alignment.

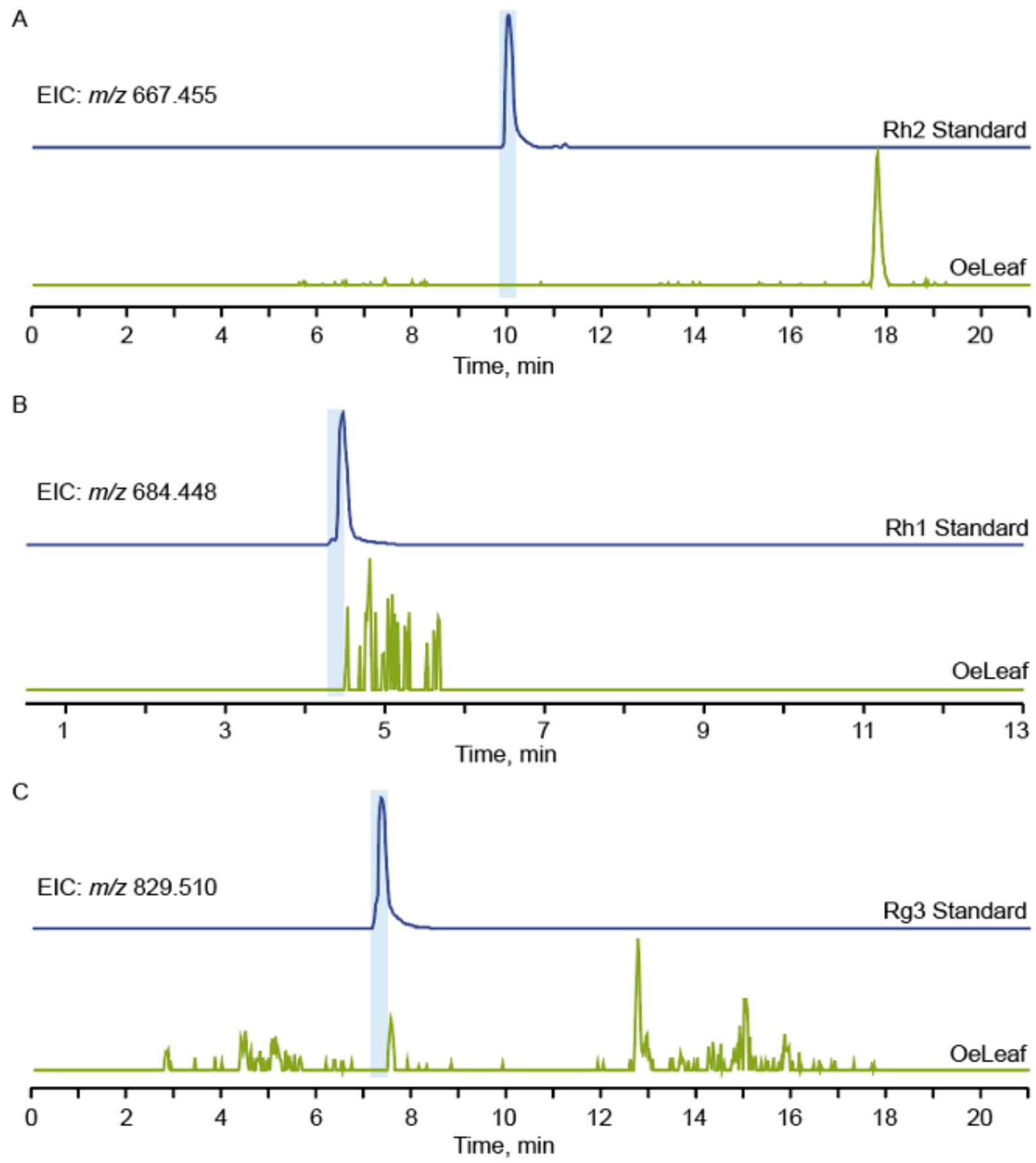

Supplementary Figure 35. LC-MS analysis of dammarane-type ginsenosides in *O. elatus*. (A) Extracted ion chromatograms of dammarane-type ginsenoside Rh2 in *O. elatus* leaves. The chromatograms were extracted at *m*/*z* 667.455 ion for ginsenoside Rh2. Stacked chromatograms are shown with each trace normalized to 100% relative intensity on the y-axis; (B) Extracted ion chromatograms of dammarane-type ginsenoside Rh1 in *O. elatus* leaves. The chromatograms were extracted at *m*/*z* 684.448 ion for ginsenoside Rh1. Stacked chromatograms are shown with each trace normalized to 100% relative intensity on the y-axis; (C) Extracted ion chromatograms of dammarane-type ginsenoside Rg3 in *O. elatus* leaves. The chromatograms were extracted at *m*/*z* 829.510 ion for ginsenoside Rg3. Stacked chromatograms are shown with each trace normalized to 100% relative intensity on the y-axis.

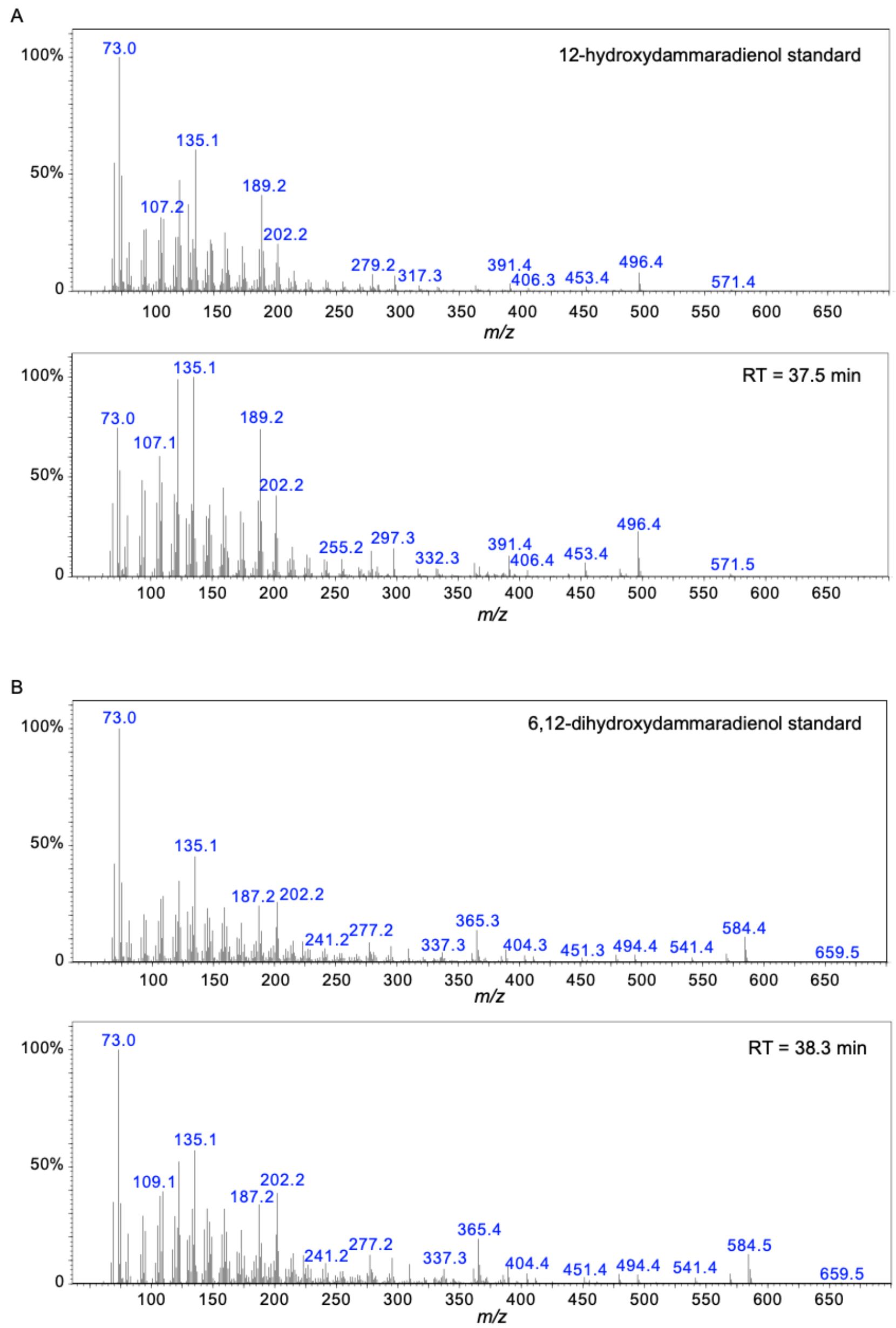

Supplementary Figure 36. Mass spectra of standards 12-hydroxydammaradienol and 6,12-dihydroxydammaradienol, alongside the products generated by the transient expression of dammaradienol synthase with PgCYP716A47 and PgCYP716A53v2 in *Nicotiana benthamiana* leaves. (A) Mass spectra of standards 12-hydroxydammaradienol alongside the products generated by the transient expression of dammaradienol synthase with PgCYP716A47 in *Nicotiana benthamiana* leaves. The retention times for the compounds are 37.5 minutes. (B) Mass spectra of standards 6,12-dihydroxydammaradienol alongside the products generated by the transient expression of dammaradienol synthase with PgCYP716A47 and PgCYP716A53v2 in *Nicotiana benthamiana* leaves. The retention times for the compounds are 38.3 minutes.

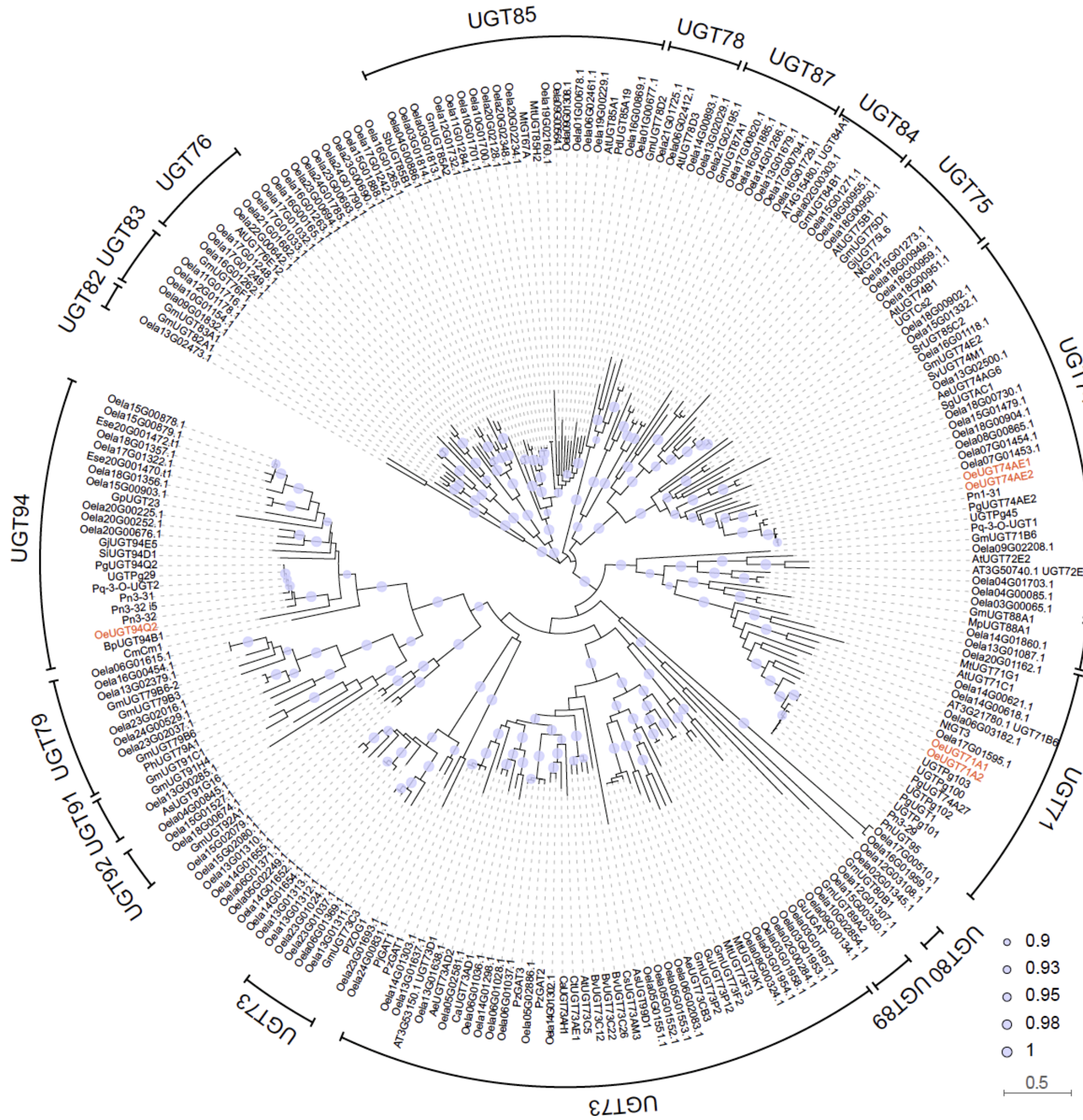

Supplementary Figure 37. Maximum-likelihood tree of glycosyltransferases (UGTs) in *O. elatus.* Filled purple circles indicate bootstrap support values for nodes greater than 0.9. Source data are provided as a Source Data file.

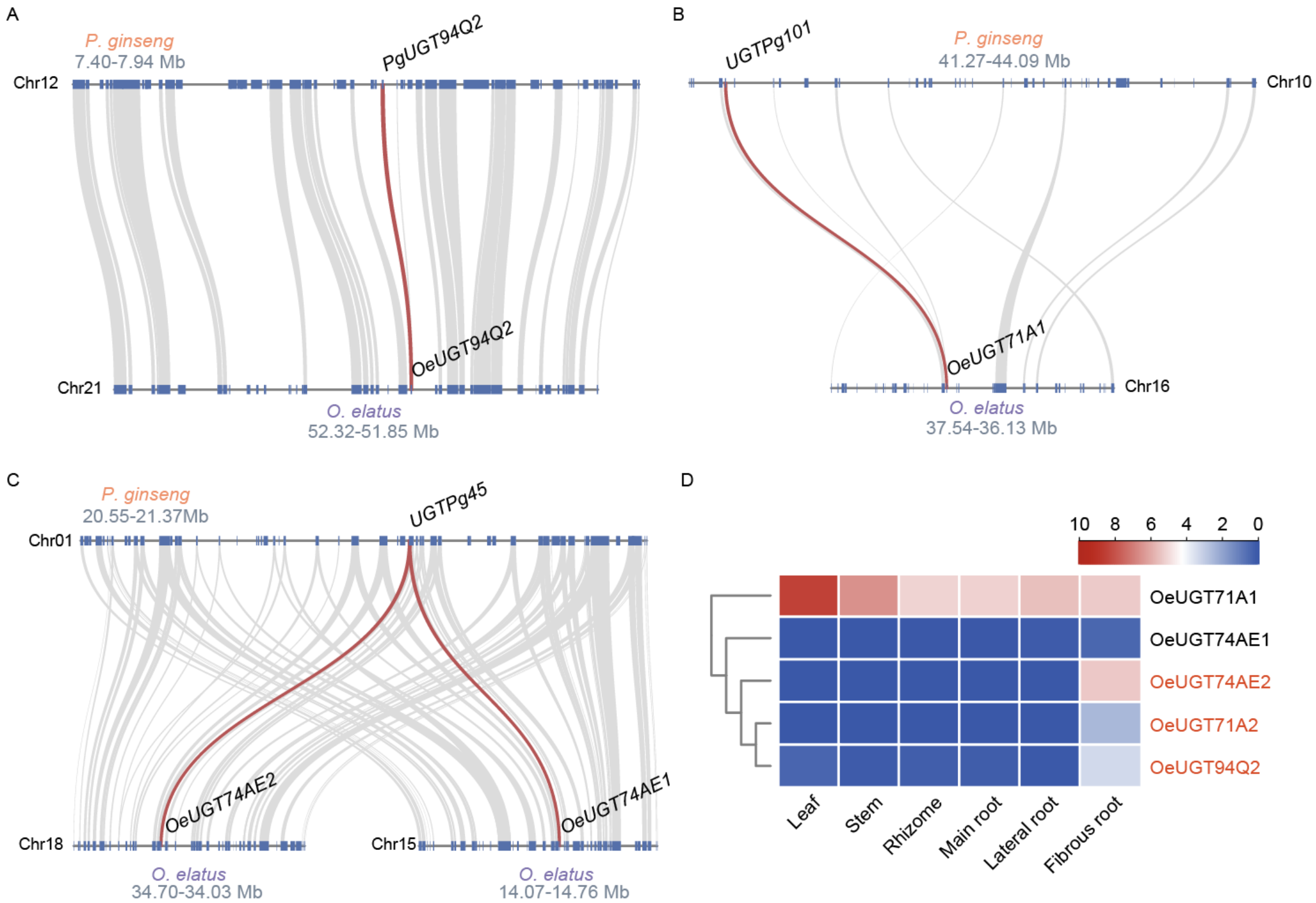

Supplementary Figure 38. Synteny analysis and expression profile of the *UGT* genes. (A) Synteny analysis of the *PgUGT94Q2* gene between *P. ginseng* and *O. elatus.* (B) Synteny analysis of the *UGTPg101* gene between *P. ginseng* and *O. elatus.* (C) Synteny analysis of the *UGTPg45* gene between *P. ginseng* and *O. elatus.* (D) Expression of *UGT* genes in six different tissues of *O. elatus*, represented by a heatmap with values computed as log2(FPKM+1). The *UGT* genes involved in the synthesis of RK-type ginsenosides are highlighted in orange. Source data are provided as a Source Data file.

Supplementary Table 1. Statistics of PacBio sequencing data.

Cell	Raw reads	Raw base (bp)	CCS reads	CCS base (bp)	N50 (bp)
Cell1	22,744,488	332,291,378,787	1,526,834	25,238,314,220	16,559
Cell2	6,295,035	77,667,809,405	356,364	4,741,901,285	13,336
Cell3	23,663,844	280,822,905,255	1,264,591	15,792,293,643	12,595

Supplementary Table 2. Statistics of Hi-C data.

Lane	Read length (bp)	Raw base (Gb)	Clean base (Gb)
V300086348_L01	150,160	142.4	126.91
total	150,160	142.4	126.91

Supplementary Table 3. Summary of statistics on *O. elatus* genome assembly.

Statistic	Size (bp)	Number
N90	1,153,742	241
N80	2,846,961	162
N70	4,214,317	119
N60	5,611,428	88
N50	7,528,359	65
Longest	26,422,303	-
Total Length	1,488,796,907	-
Total Number	-	964
GC Rate	0.361	

Supplementary Table 4. BUSCO analysis of *O. elatus* chromosome-level genome assembly.

Type	Number	Percentage (%)
Complete BUSCOs (C)	1,604	99.38
Complete and single-copy BUSCOs (S)	985	61.03
Complete and duplicated BUSCOs (D)	619	38.35
Fragmented BUSCOs (F)	3	0.19
Missing BUSCOs (M)	7	0.43
Total BUSCO groups searched	1,614	-

Supplementary Table 5. Chromosome statistics of *O. elatus* subgenome.

Subgenome A				Subgenome B			
**ID**	**Name**	**Contig**	**Length**	**ID**	**Name**	**Contig**	**Length**
Chr01	A1	14	44247738	Chr02	B1	22	53395218
Chr03	A2	17	58463223	Chr04	B2	58	50731514
Chr05	A3	23	73400397	Chr06	B3	22	73127541
Chr07	A4	51	55891963	Chr08	B4	22	51950835
Chr09	A5	28	65346238	Chr10	B5	25	63323015
Chr11	A6	17	55899542	Chr12	B6	64	79434117
Chr14	A7	15	63972106	Chr13	B7	46	55991762
Chr15	A8	20	52385989	Chr16	B8	17	57557307
Chr18	A9	21	47481614	Chr17	B9	13	65314341
Chr19	A10	78	56987935	Chr20	B10	12	68267878
Chr22	A11	52	64685381	Chr21	B11	19	58231253
Chr24	A12	16	65402051	Chr23	B12	55	72305026

Supplementary Table 6. Statistics of gene annotation for *O. elatus.*

Annotation method	Gene set	Number	Average gene length(bp)	Average CDS length(bp)	Average exon per gene	Average exon length(bp)	Average intron length(bp)
De novo	**Augustus**	293,290	2532.06	1139.23	2.94	387.8	718.81
De novo	**SNAP**	439,283	2244.99	728.5	2.83	257.79	830.5
**homolog**	***A. graveolens***	37,338	4610.24	1297.19	5.5	235.86	736.27
**homolog**	***A. thaliana***	38,190	4634.4	1287.99	5.63	228.82	722.95
**homolog**	***A. elata***	50,960	4627.46	1236.5	5.28	234.36	793.0
**homolog**	***C. sativum***	39,124	4216.01	1237.82	5.15	240.37	717.7
**homolog**	***D. carota***	40,685	4553.23	1252.67	5.36	233.72	757.05
**homolog**	***E. senticosus***	48,987	4184.47	1335.84	4.91	271.81	727.7
**homolog**	***P. ginseng***	60,339	3840.03	1121.86	4.61	243.34	752.9
**homolog**	***P. notoginseng***	53,120	3373.21	1330.11	3.81	349.41	727.94
**RNA-seq**	**transcript**	96,804	6527.05	1019.78	4.64	219.97	1514.7
**Final**	-	61,842	5165.21	1211.96	4.84	250.17	1028.28

Supplementary Table 7. Statistics of functional annotation for coding gene in *O. elatus*.

Type	Number	Percentage
**Total**	61,842	-
**Nr**	56,711	91.70%
**Swissprot**	43,053	69.62%
**KEGG**	45,003	72.77%
**KOG**	43,527	70.38%
**TrEMBL**	56,480	91.33%
**Interpro**	52,797	85.37%
**GO**	40,681	65.78%
**Overall**	56,900	92.01%

Supplementary Table 8. Summary of repeat elements in *O. elatus* genome.

Type	Repeat length(bp)	% of genome
**Trf**	170,130,158	11.427358
**Repeatmasker**	192,694,532	12.942970
**Proteinmask**	187,036,363	12.562920
De novo	902,055,468	60.589558
**Total**	947,996,282	63.675326

Supplementary Table 9. Repeat elements annotation of *O. elatus*.

Type		Length (bp)	% of genome
**Retro**	**LTR/Copia**	244,863,911	16.4471
**Retro**	**LTR/Gypsy**	600,650,499	40.34469
**Retro**	**LTR/Other**	29,416,151	1.97583
**Retro**	**SINE**	383,886	0.02578
**Retro**	**LINE**	23,931,089	1.60741
**Retro**	**Other**	0	0.0
**DNA**	**EnSpm**	13,860,140	0.93096
**DNA**	**Harbinger**	2,020,865	0.13574
**DNA**	**hAT**	12,781,493	0.85851
**DNA**	**Helitron**	4,121,493	0.27683
**DNA**	**Mariner**	6,530	0.00044
**DNA**	**MuDR**	4,494,889	0.30191
**DNA**	**P**	1,091,038	0.07328
**DNA**	**Other**	7,478,804	0.50234
**Other**	**-**	5,890,520	0.39566
**Unknown**	**-**	49,377,009	3.31657
**Total**	**-**	925,141,582	62.14021

Supplementary Method 1. Preparation of 12-hydroxydammaradienol and 6,12-dihydroxydammaradienol reference standards by glycosidase-catalyzed hydrolysis

The gene encoding the glycosidase *BglSK* was synthesized and cloned into the pET-14b expression vector which was described in previous study[1]. The recombinant BglSK protein was expressed and purified by affinity chromatography using its N-terminal His tag. For ginsenoside hydrolysis, reactions were carried out in a total volume of 100 μL, containing 50 mM Tris–HCl buffer, pH 8.0, 20 μg of substrate (ginsenoside Rk2 or ginsenoside Rk3), and 2 μM purified BglSK. The reaction without enzyme was as negative control. All the reaction mixtures were incubated at 37 °C for 24 h. After incubation, each reaction was extracted with an equal volume (100 μL) of *n*-butanol. The organic phase was collected, evaporated to dryness, and reconstituted in methanol prior to LC–MS analysis.

The LC-MS analysis was performed using a LC-MS 9030 quadrupole time-of-flight liquid chromatograph mass spectrometer (Shimadzu, Japan). Chromatographic separation was performed using a Welch C18 column (2.1 × 100 mm, 1.8 μm), with a mobile phase consisting of acetonitrile (B) and 0.1% formic acid in water (A) at a flow rate of 0.2 mL/min. The linear gradients of solvent B as follows: 0 to 2 min, 30% B; 2 to 7 min, 30 to 60% B; 7 to 8 min, 60 to 90% B; 8 to 10 min, 90 to 100% B; 10 to 13 min, 100% B, 13 to 14 min, 100% to 30% B and 14 to 15 min 30% B. The column oven temperature was set at 40 °C. The total time of program was 15 min. Mass spectrometry analysis was carried out in data-dependent acquisition (DDA) mode using positive ionization mode of electrospray ionization (ESI), monitoring full spectra from *m*/*z* 100–1500 (100 ms per spectrum) and the data-dependent MS/MS spectra (60 ms per spectrum) with 35% collision energy (CE).

Supplementary reference

1. Cao, L. et al. Highly efficient production of diverse rare ginsenosides using combinatorial biotechnology. *Biotechnology and Bioengineering.* **117**, 1615-1627 (2020).
