# Supplementary material for: The Oplopanax elatus genome reveals dammaradienol synthase evolution enabling reconstruction of RK type ginsenosides biosynthesis
Source: Nat Commun. 2026 Jun 24;17:7907. doi: 10.1038/s41467-026-74686-6 (PMC13444123; doi:10.1038/s41467-026-74686-6)
Supplement: Supplementary file 3 [file 41467_2026_74686_MOESM2_ESM.pdf]

nature portfolio

Peer Review File

The *Oplopanax elatus* genome reveals dammaradienol synthase evolution enabling reconstruction of RK type ginsenosides biosynthesis

Corresponding Author: Professor Yu Wang

This file contains all reviewer reports in order by version, followed by all author rebuttals in order by version.

Version 0:

Reviewer comments:

Reviewer #1

(Remarks to the Author)
In this study, Wang and co-authors present an interesting related to the evolution of a dammaradienol synthase in the family Araliaceae. They found that neofunctionalization of the duplicated copy (OeOSC14) results in the evolution of a new ginsenoside in P. elatus. I listed my concerns below for the authors' reference.
Section Abstract,
1. I would suggest the authors rewrite this section, the current version is not clear, it is really hard to get useful information. It should be better if the some details are included, such as the amino acid mutation of OeOSC;
Section Introduction,
2. line 65, should cite references at the end of this sentence;
Section Introduction,
3. line 73, I do not think it is a good choice to cite a figure in this section, In particular, this figure does not include too much useful information. Please combine it with figure 2 or remove it;
4. in Figure 3A, the 24 chromosomes are named as A1-12 and B1-12, however, the authors use "Chr1-24" in the other figures and main text. Please make changes to ensure all are the same;
5. line 259, where is the Figure 4E?
6. Lines 278-288, also the Figure 5, I do not agree with the authors to define the "Clade III" is an evolutionarily clade. Firstly, only two species are defined as outgroup. I would suggest to include some Apiaceae species (such as carrot) to infer the genome collinearity; Secondly, I am not sure if any Araliaceae species are included in the Clade I. From what I see in Figure 5C, for example, three copies are identified in Panax ginseng, the chromosome pair PgChr17 and PgChr2 are duplicated by Pg-a. However, PgChr13 was duplicated by Pg-beta. It indicates that Clade II and Clade III are derived by Pg-beta. The DDS copy was lost in O. elatus (the same as what the authors observed in A. elata). Under this hypothesis, the three OSC genes (OeOSC12/13/14) are duplicated by small scale duplication mode (not tandem duplication in line 312).
7. Line 319, why OeOSC12/13 are not included? It seems that OeOSC12 is also a candidate. In the case if N260Y is important, how about the amino acid at the same position in the two genes?
8. The Figure 7 and 8 are very similar, I would suggest combine the two figures or move to supplementary file. Five figures are enough in main text.
Section Methods and Materials,
9. Line 493, why highlight with red?
Overall, I think this is an interesting study that provides important insights on how the ginsenosides have evolved and diversified in Araliaceae.

Reviewer #2

(Remarks to the Author)
This study explored the reason for the absence of RK-type ginsenosides in Oplopanax elatus by genome assembly, comparative analysis, and pathway elucidation. One amino acid mutation (N260) in OSC shifts the product specificity from α/β-amyrin to dammarodienol. However, pseudogenization of the oxidative gene blocks the pathway to further PK-type ginsenosides. The authors also confirmed this hypothesis by reconstructing the biosynthetic pathway of PK-type

ginsenosides in N. bentheminana leaf through compensating with CYP450 genes from Panax ginseng. This study exemplifies the evolution of specialized metabolic pathways, with identified mechanisms valuable for synthetic biology. However, I have some concerns regarding the OSC's functional evolution and the loss-of-function of cytochrome P450 oxidase.

Major

Please provide the docking results for OeOSC14N260Y, SbxAS, and SbxAS Y260N, with dammaradienol or -amyrin, and confirm if the distance between substrate and N/Y residue exceeds 5 Å.

The authors provide a clear evolutionary inference that OeDDES and Panax DDS originated from a common BAS-like ancestor, and that OeOSC12, OeOSC13, and OeOSC14 arose from tandem duplication events within O. elatus. OeOSC12 catalyzes the formation of dammaradienol as well as four additional products, whereas OeOSC14 appears to be highly specific for dammaradienol production. In contrast, the ancestral homologs (EsXAS, PgXAS, and SbXAS) do not produce dammaradienol, suggesting that OeOSC12 represents a key neofunctionalized enzyme in the evolution of OeDDES activity. The authors may need to include OeOSC12, EsXAS, and PgXAS in the alignment in Figure 6A, as they are closer to OeOSC14. Depending on the similarity, those three enzymes may also require function verification in Figure 6B or docking analysis.

Please clarify the extent of loss in the CYP716A47 homolog (OeCYP716: 7633.2 kb - 7633.8 kb) in O. elatus and confirm if the remnant sequence encodes a functional protein. If so, the authors may need to test its activity.

The authors should consider whether there is another CYP716A47 homolog in the second subgenome of O. elatus. If so, clarify whether it also has undergone pseudogenization.

Although there is no direct evidence, the authors may discuss why O. elatus lost PK ginsenosides. Possible reasons include metabolic balance shunts or lack of essentiality for this species. The physiological role of PK ginsenosides in Araliaceae should also be addressed.

Although PgXAS shares high sequence similarity with OeOSC12, it cannot catalyze dammaradienol formation. The authors may need to explain why P. ginseng cannot synthesize RK ginsenosides endogenously.

Minor

Supplemental Figure 23 is missing.

The retention time described in lines 253-256 seems slightly different from that shown in Figure 4B.

Figure 2A: Please add the scale bars

Line 484, 579, 652, 659, legend of Figure 3B, 7, 8A: italicized species names

Line 555, 572-578, 620, 625, 684: Add space between numbers and units, and outside parenthesis

Line 671: keep capitalization of the title consistent with that of the others

Reviewer #3

(Remarks to the Author)

This manuscript addresses triterpenes and triterpene saponins, which are important medicinal compounds with high potential for pharmaceutical development. Ginseng (Panax ginseng), a member of the Araliaceae family, is well known for its unique and diverse saponins derived from the dammarenediol-II skeleton.

Although dammarenediol-II synthase–like genes have been reported in Araliaceae plant species other than ginseng, the reason why dammarenediol-II is not produced in these species remains unclear. Furthermore, the identification of triterpenes possessing various dammarene skeletons except for dammarenediol-II and functional characterization of the genes involved in their biosynthesis are still poorly understood across plant species.

In this study, the authors present the first genome sequencing of Oplopanax elatus and provide insights into the evolutionary history of dammarene-type triterpene biosynthesis through comparative analysis of triterpene synthase genes in Araliaceae plants. Importantly, through heterologous expression in yeast, the authors demonstrate for the first time in Araliaceae the existence of a gene encoding a dammarandiol synthase rather than a dammarenediol-II synthase. The functional characterization of OeOSC14 and the analysis of amino acid residue specificity underlying its product selectivity are of particular academic significance.

Oplopanax elatus is a medicinal plant native to Northeast Asia and, like ginseng, belongs to the Araliaceae family, in which saponins are known to be major bioactive constituents. Previous chemical studies have reported the production of dammarandiol acetate in Oplopanax horridus, a closely related species, suggesting the presence of a dammarandiol synthase. In this manuscript, the authors demonstrate that dammarandiol is a representative triterpene in Oplopanax elatus and identify the gene responsible for its biosynthesis. Among several genes homologous to the ginseng dammarenediol-II synthase, the authors convincingly show for the first time that OeOSC14 encodes a dammarandiol synthase.

Although dammarandiol is sometimes classified as a tetracyclic triterpene, it can also be categorized as a polyene-type compound due to the presence of multiple double bonds within its skeleton, which is associated with strong pharmacological activity. In ginseng, polyene-type ginsenosides such as Rk1, Rk2, and Rk3 exhibit potent bioactivity; however, these compounds are not naturally produced and are instead generated through thermal degradation of ginseng saponins. Based on the identification of a dammarandiol synthase from Oplopanax elatus, this study is highly valuable in that it reports the development of a synthetic biology approach enabling the production of ginsenosides Rk1, Rk2, and Rk3 for the first time. Moreover, the authors provide an explanation for why Oplopanax elatus, despite possessing a dammarandiol synthase, does not naturally produce ginsenosides Rk1, Rk2, and Rk3.

Overall, this manuscript presents findings that advance our understanding of triterpene biosynthesis and evolution in Araliaceae plants and offers implications for synthetic biology–based production of pharmacologically active ginsenosides. However, in order to be suitable for publication in this journal, several issues should be addressed and further clarification and strengthening of the manuscript are required, as outlined below.

The following issues should be addressed:

1. A previously published study reporting the presence of dammarandiol acetate in Oplopanax horridus should be properly

cited.
2. Although a high level of dammarandiol is detected in the leaves and petioles of Oplopanax elatus, no dammarandiol-derived saponins have been reported to date. Further discussion is required to explain why saponins derived from dammarandiol have not been identified in this species.
3. It would be worthwhile to consider the possibility that, similar to Oplopanax horridus, dammarandiol in Oplopanax elatus may be converted into dammarandiol acetate and stored primarily in this acetylated form rather than as saponins. Additional speculation on the potential natural roles of these compounds would strengthen the discussion.
4. A dammaradienol synthase, AarOSC20433, was identified for the first time in Artemisia argyi. This study should be cited, and a comparative analysis of the amino acid sequence of this enzyme with OeOSC14 would be informative.
5. The Introduction should include information on the major triterpenes and saponins that have been reported in Oplopanax elatus.
6. At line 77, ginsenoside Rk1 is described as having the strongest activity. Additional explanation and supporting evidence are required, particularly citing Reference 11, which reports the biological activity of ginsenoside Rk1.
7. At line 111, the phrase "dammaradienol in its leaves but not RK-type ginsenosides" should be revised to "dammaradienol in its leaves but not hydroxylated dammaradienol or glycosylated sapogenins (RK-type ginsenosides)" for greater clarity and accuracy.
8. In Figure 5, the order of the legends for panels C and D appears to be incorrect and should be reversed.
9. To date, no studies have reported genes responsible for the biosynthesis of ocotillol-type triterpenes. In Figure 5C, it is unclear whether the DDS genes belonging to clade III are proposed to produce ocotillol-type triterpenes. The authors should clarify whether this is supported by experimental evidence or is merely speculative.
10. The authors should discuss whether overexpression of OeOSC14 in ginseng plants could potentially enhance the production of RK-type ginsenosides (Rk1, Rk2, and Rk3).
11. In Figure 6D, the term "Deprotonation" alone is insufficient. The description would be clearer if revised to "Deprotonation-mediated double bond formation."
12. Oplopanax elatus is likely to possess multiple cytochrome P450 (CYP) genes involved in the conversion of triterpenes into saponins. Although this is not the primary focus of the present study, discussion of potential biosynthetic pathways and synthetic circuits for reported saponins such as cirenshenosides S, T, U, and V would add value to the manuscript.
13. The new results showing that co-expression of OeOSC14 with two ginseng-derived CYP genes responsible for protopanaxadiol and protopanaxatriol biosynthesis enables the production of 12-hydroxydammaradienol and 6,12-dihydroxydammaradienol in tobacco are particularly interesting. Furthermore, the functional characterization of Oplopanax elatus–derived UGTs (OeUGT74AE2, OeUGT94Q2, and OeUGT71A2) and the successful reconstruction of a biosynthetic pathway leading to the production of RK-type ginsenosides (Rk1, Rk2, and Rk3) represent a significant achievement. These results strongly suggest the feasibility of synthesizing RK-type ginsenosides in ginseng and other plant systems in the future.
14. Finally, the reference list requires careful revision, as the use of uppercase and lowercase letters in the titles is inconsistent and does not follow a uniform formatting rule.

Reviewer #4

(Remarks to the Author)
In this study, leveraging genome sequencing of Oplopanax elatus, the authors identified OeOSC14, an oxidosqualene cyclase responsible for the biosynthesis of dammaradienol, the precursor of RK-type ginsenosides. They then investigated the evolutionary origin of OeOSC14 through phylogenetic and comparative genomic analyses. Furthermore, multiple sequence alignment, site-directed mutagenesis, and QM/MM multiscale simulations were employed to identify a key amino acid residue, N260, that governs the dammaradienol synthase specificity of OeOSC14. Finally, by combining PgCYP716A47 and PgCYP716A53v2 from Panax ginseng with three newly identified glycosyltransferases from O. elatus, the authors successfully reconstituted the biosynthetic pathway of RK-type ginsenosides in Nicotiana benthamiana. Nevertheless, several issues and questions regarding this study remain:

1. A dammaradienol synthase, AarOSC20433, has been identified from Artemisia argyi (ACS Synth. Biol. 2024, 13, 12, 4015–4026). The authors should cite this enzyme and discuss it appropriately in the manuscript.

2. The results shown in Figure 3B do not correspond to the descriptions provided in the manuscript.

3. Lines 241–245. These descriptions are confusing. The data presented in Figure 4A and Supplementary Figure 20 do not support these statements. It is unclear how the authors conclude that 15 triterpene-related OeOSC genes are predominantly expressed in leaves based on these data. Moreover, the eight candidates selected for functional characterization do not appear to meet the stated selection criterion, namely the exclusion of lowly expressed genes (FPKM < 1).

4. Based on Figure 4B, transient expression of OeOSC12 produced five, rather than four, additional peaks compared with the control, and transient expression of OeOSC14 produced two additional peaks rather than one. What are the identities of these peaks?

5. In the left panel of Figure 5A and in Supplementary Fig. 22, OeDDES should be highlighted using a distinct color or symbol to facilitate its identification. From an evolutionary perspective, plant triterpene synthases are thought to have originated from cycloartenol synthase (CAS). This view is consistent with the phylogenetic tree shown in Figure 4A, but appears to be inconsistent with the phylogenetic trees presented in Figure 5A (left) and Supplementary Fig. 22. How should these apparently conflicting results be interpreted?

6. Supplementary Figure 23 is not shown in the Supplementary Information.

7. The data presented are not sufficient to support the conclusion that “dammaradienol synthase emerged from an ancestral β-amyrin synthase through gene duplication, neofunctionalization, and subsequent functional specialization.” Based on the phylogenetic tree shown in Figure 5A and the synteny analysis in Figure 5C (note that the panel is labeled C in the figure but D in the legend), an alternative interpretation is that OeDDES may have arisen from an mTTS via gene duplication, neofunctionalization, and subsequent functional specialization. Additional synteny analyses are necessary to substantiate a proposed evolutionary origin of mTTS from BAS.

8. The syntenic blocks containing mTTS are well conserved among the eight Araliaceae species (Figure 5C). However, although no mTTS is present in the corresponding syntenic block of E. senticosus, putative mTTSs (EsχAS) that may have arisen through translocation events were identified at other chromosomal locations. By the same logic, the absence of a full-length CYP716A gene in the corresponding syntenic region of O. elatus does not necessarily indicate the loss of a C12 hydroxylase. Have the authors considered functionally characterizing Oela19G01405.1, which clusters in the same clade as PgCYP716A47 in the phylogenetic tree?

In addition, the failure to detect 12-hydroxydammaradienol, 6,12-dihydroxydammaradienol, or RK-type ginsenosides in different tissues of O. elatus may also result from spatiotemporal mismatches between the expression of the upstream gene OeOSC14 and downstream pathway genes. For example, the newly identified glycosyltransferases OeUGT94Q2, OeUGT74AE2, and OeUGT71A2 exhibit predominant expression in roots rather than leaves.

9. There are multiple instances in the manuscript with grammatical or logical issues, for example in lines 100–103, 108–111, and 142–145. For clarity, please provide the full term when an abbreviation is used for the first time (e.g., BAS).

Version 1:

Reviewer comments:

Reviewer #1

(Remarks to the Author)
All my previous comments were fully addressed by the authors. I appreciate the authors for their efforts to revise the manuscript. This study provides important insights on how the triterpenoid saponins have diversified. I have no more comments.

Reviewer #2

(Remarks to the Author)
The authors have addressed all comments, and I have no further suggestions.

Reviewer #3

(Remarks to the Author)
Overall, all questions and revision requirements raised by me have been addressed, and the responses are considered appropriate.

However, there are still a few points to be addressed; therefore, further revisions are required as follows

Regarding 'dammarenediol-II,' the usage of the hyphen is inconsistent. I recommend unifying it to a single format throughout the manuscript.

An Rtx-5MS capillary column (30m× 0.25mm×0.25 μm, Shimadzu) was used for the isolation of triterpene.

The term 'separation' seems more suitable than 'isolation' for the process described in this sentence

I noticed that some journal names in the references are written in full, while others use abbreviations. I suggest standardizing them all to the appropriate abbreviations. Please review the references thoroughly for consistency.

In several references, full journal names are still used.

European Food Research and Technology
Journal of the American Chemical Society.
Plant Biotechnology Journal
ACS Synthetic Biology.
Chinese Herbal Medicines.
Pharmaceutical Chemistry Journal.
Nucleic Acids Research.

The references listed below are incomplete. Please provide the missing details.
Telomere-to-telomere reference genome for Panax ginseng highlights the evolution of saponin biosynthesis. (no further information)

66. Eddy, S. R. Accelerated Profile HMM Searches. PLoS Comput Biol. 7, e1002195 (2011).
Accelerated profile HMM Searches

Reviewer #4

(Remarks to the Author)
1. The manuscript would benefit from improved conciseness, as certain sections contain redundant or overly detailed descriptions. Some of this information could be more appropriately placed in the Methods section or supplementary tables, rather than being extensively described in the main text—for instance, lines 184–188.

2. The explanation of the mechanistic role of the key residue Asn (N260) in Lines 387–416, together with the results presented in Fig. 5D, is not sufficiently clear and is difficult to follow. Specifically, illustrating either the distance between the hydrogen atom on the side-chain amide of Asn (N260) and the carbon atoms of the product dammaradienol, or the distance between the phenolic hydroxyl hydrogen of Tyr (Y260/259) and the carbon atoms of the phenyl ring of Phe (F413/412), does not adequately explain how residue N260 influences product structure formation.

3. Based on the description in Lines 503–505 and the data presented in Supplementary Figs. 36–37, it is not clear how OeUGT71A2 was identified.

4. Transient expression of OeOSC14 together with tHMGR in tobacco leaves yields nearly 4 mg/g DW (Fig. 5c). However, upon introduction of downstream pathway genes, the highest reported yield of the final products RK1/2/3 reaches only 23.07 μg/g DW. This substantial decrease suggests that the downstream hydroxylases and/or glycosyltransferases may have relatively low catalytic efficiency under the tested conditions, potentially limiting their suitability for efficient production of RK-type triterpenoid saponins.

Version 2:

Reviewer comments:

Reviewer #4

(Remarks to the Author)
I have no further comments and recommend acceptance of the manuscript.

Open Access This Peer Review File is licensed under a Creative Commons Attribution 4.0 International License, which permits use, sharing, adaptation, distribution and reproduction in any medium or format, as long as you give appropriate

credit to the original author(s) and the source, provide a link to the Creative Commons license, and indicate if changes were made.
In cases where reviewers are anonymous, credit should be given to 'Anonymous Referee' and the source.
The images or other third party material in this Peer Review File are included in the article's Creative Commons license, unless indicated otherwise in a credit line to the material. If material is not included in the article's Creative Commons license and your intended use is not permitted by statutory regulation or exceeds the permitted use, you will need to obtain permission directly from the copyright holder.
To view a copy of this license, visit https://creativecommons.org/licenses/by/4.0/

REVIEWER COMMENTS

Reviewer #1 (Remarks to the Author):

In this study, Wang and co-authors present an interesting related to the evolution of a dammaradienol synthase in the family Araliaceae. They found that neofunctionalization of the duplicated copy (OeOSC14) results in the evolution of a new ginsenoside in *O. elatus*. I listed my concerns below for the authors' reference.

Section Abstract,

Comment 1: I would suggest the authors rewrite this section, the current version is not clear, it is really hard to get useful information. It should be better if the some details are included, such as the amino acid mutation of OeOSC;

Response 1: We thank the reviewer for this helpful suggestion. We agree that the original Abstract was not sufficiently clear and did not adequately present the key mechanistic findings. We have therefore substantially revised the Abstract. In particular, we now explicitly identify OeOSC14 as a specialized dammaradienol synthase, include the key amino acid substitution OeOSC14^{N260Y} that shifts product specificity to a multifunctional activity producing α-amyrin, β-amyrin, and dammarenediol II, clarify that loss of C12 hydroxylase blocks the oxidative tailoring step required for RK-type ginsenoside biosynthesis in *O. elatus*, and state that pathway reconstruction in *Nicotiana benthamiana* enables de novo production of Rk1, Rk2, and Rk3. We believe the revised Abstract is now clearer and more informative, and better reflects the main findings of the study.

Section Introduction,

Comment 2: line 65, should cite references at the end of this sentence;

Response 2: We thank the reviewer for pointing this out. We have added the relevant reference at the end of the sentence to strengthen the supporting evidence for this statement (Please see line 67).

Section Introduction,

Comment 3: line 73, I do not think it is a good choice to cite a figure in this section, In particular, this figure does not include too much useful information. Please combine it with figure 2 or remove it;

Response 3: We thank the reviewer for this helpful suggestion. In the revised manuscript, we have removed the original Fig. 1 from the Introduction and incorporated the relevant content into Fig. 2 (now Fig. 1) to improve the continuity and logical flow of the figure presentation.

Comment 4: in Figure 3A, the 24 chromosomes are named as A1-12 and B1-12, however, the authors use "Chr1-24" in the other figures and main text. Please make changes to ensure all are the same;

Response 4: We thank the reviewer for this careful observation. We have revised the chromosome nomenclature throughout the main text and all figures, and now consistently use "Chr1–24" across the manuscript.

Comment 5: line 259, where is the Figure 4E?

Response 5: We thank the reviewer for pointing this out. We have corrected the figure citation in the revised manuscript so that the relevant text now properly refers to Fig. 3E (formerly Fig. 4E). The corresponding change can be found in line 276.

Comment 6: Lines 278-288, also the Figure 5, I do not agree with the authors to define the "Clade III" is an evolutionarily clade. Firstly, only two species are defined as outgroup. I would suggest to

include some Apiaceae species (such as carrot) to infer the genome collinearity; Secondly, I am not sure if any Araliaceae species are included in the Clade I. From what I see in Figure 5C, for example, three copies are identified in *Panax ginseng*, the chromosome pair PgChr17 and PgChr2 are duplicated by Pg-a. However, PgChr13 was duplicated by Pg-beta. It indicates that Clade II and Clade III are derived by Pg-beta. The DDS copy was lost in O. elatus (the same as what the authors observed in *A. elata*). Under this hypothesis, the three OSC genes (OeOSC12/13/14) are duplicated by small scale duplication mode (not tandem duplication in line 312).

Response 6: We thank the reviewer for this important comment. We agree that our original wording overstated the evolutionary interpretation of the OSC groups shown in Fig. 5. In the revised manuscript, we no longer refer to "Clade III" as an evolutionary clade. Instead, we describe these categories as BAS-like, mTTS-like, and DDS-like groups, reflecting phylogenetic relationships and catalytic tendencies rather than a strictly linear evolutionary sequence. We also revised the collinearity analysis by including additional comparative genomes, including *Centella asiatica* (Apiaceae), *Artemisia argyi* (Asteraceae) and updated Fig. 5C accordingly. The revised synteny results support differential retention of two related OSC-associated branches after the shared Araliaceae genome duplication, with DDS-like copies retained in *Panax* but pseudogenized or lost in *A. elata* and *O. elatus*, whereas the mTTS-associated branch was retained in multiple Araliaceae lineages. In addition, we have revised the description of OeOSC12/13/14 to indicate that these genes most likely arose through small-scale duplication and local genome rearrangement, rather than tandem duplication. Overall, the revised text now presents a more cautious and data-supported evolutionary model for the origin of OeOSC14 and the diversification of mTTS- and DDS-associated OSC loci. The corresponding revisions can be found in lines 296–339 and in the revised Fig. 5.

Comment 7: Line 319, why OeOSC12/13 are not included? It seems that OeOSC12 is also a candidate. In the case if N260Y is important, how about the amino acid at the same position in the two genes?

Response 7: We thank the reviewer for this insightful comment. We agree that OeOSC12 is also an important candidate in the context of residue 260 and that our original presentation did not make this sufficiently clear.

But, OeOSC13 was not included in the mutational analysis because it showed no detectable catalytic activity in our transient expression assays, and we therefore could not meaningfully assess the functional effect of the residue at the corresponding position.

By contrast, OeOSC12 has now been included in the revised analysis. We show that OeOSC12, although highly similar to OeOSC14, carries tyrosine at position 260 and exhibits a multifunctional product profile that includes dammaradienol. We further introduced the reciprocal substitution into OeOSC12 and found that the OeOSC12^{Y260N} mutant shifted toward a simplified product profile, with dammaradienol becoming the major detectable product. These results support the importance of residue N/Y at position 260 as a key determinant of product specificity. The corresponding revisions can be found in lines 378–386.

At the same time, our results also suggest that residue 260 alone is not sufficient to fully explain dammaradienol synthase activity. In particular, although OeOSC12 contains tyrosine at position 260, it still produces a detectable, albeit low, amount of dammaradienol, suggesting that residue 260 is important but not solely responsible for directing dammaradienol formation. We have therefore noted in the revised Discussion that the structural basis of dammaradienol synthesis is likely

determined by the combined effects of residue 260 and other surrounding residues, and that this mechanism will require further investigation in future work. The corresponding revisions can be found in lines 399–416.

Comment 8: The Figure 7 and 8 are very similar, I would suggest combine the two figures or move to supplementary file. Five figures are enough in main text.

Response 8: We thank the reviewer for this constructive suggestion. We carefully considered combining the original Figs. 7 and 8; however, because they address different questions and contain substantial data, merging them into a single figure would reduce clarity and make the presentation overly crowded. At the same time, we agree that some information was redundant. We therefore simplified the figure presentation by removing the partial pathway diagram overlaid on the chromatogram in the original Fig. 8. In the revised manuscript, these figures are now presented as Figs. 6 and 7.

Section Methods and Materials,

Comment 9: Line 493, why highlight with red?

Response 9: We thank the reviewer for pointing this out. The correction has been made in the revised manuscript.

Comment 10: Overall, I think this is an interesting study that provides important insights on how the ginsenosides have evolved and diversified in Araliaceae.

Response 10: We thank the reviewer for this positive comment and for recognizing the significance of our study.

Reviewer #2 (Remarks to the Author):

This study explored the reason for the absence of RK-type ginsenosides in *Oplopanax elatus* by genome assembly, comparative analysis, and pathway elucidation. One amino acid mutation (N260) in OSC shifts the product specificity from α/β-amyrin to dammarodienol. However, pseudogenization of the oxidative gene blocks the pathway to further PK-type ginsenosides. The authors also confirmed this hypothesis by reconstructing the biosynthetic pathway of PK-type ginsenosides in *N. bentheminana* leaf through compensating with *CYP450* genes from *Panax ginseng*. This study exemplifies the evolution of specialized metabolic pathways, with identified mechanisms valuable for synthetic biology. However, I have some concerns regarding the OSC's functional evolution and the loss-of-function of cytochrome P450 oxidase.

Major

Comment 1: Please provide the docking results for OeOSC14^{N260Y}, SbχAS, and SbχAS^{Y260N}, with dammaradienol or β-amyrin, and confirm if the distance between substrate and N/Y residue exceeds 5 Å.

Response 1: We thank the reviewer for this helpful suggestion. In response, we have now included the docking results for OeOSC14^{N260Y}, SbχAS, and SbχAS^{Y259N} with dammaradienol and/or β-amyrin in the revised manuscript (Fig. 5D and Supplementary Fig. 25). In OeOSC14, residue N260 is positioned within 5 Å of the docked dammaradienol. By contrast, in OeOSC14^{N260Y} and SbχAS, the corresponding tyrosine residue at position 259/260 is oriented toward the conserved F413/F412 residue, and the distance between the docked substrate and this Tyr residue is greater than 5 Å. In the reciprocal mutant SbχAS^{Y259N}, this Tyr–Phe interaction is no longer observed, and the

substituted N259 is repositioned closer to the docked substrate, with the substrate–residue distance falling within 5 Å, similar to the docking pattern observed for OeOSC14. These results are consistent with our revised model that residue 259/260 influences the orientation of the substrate in the active pocket and thereby contributes to the shift between dammaradienol production and pentacyclic triterpene formation. We have clarified these points in the revised text and figures (lines 399–416; Fig. 5D and Supplementary Fig. 25).

Comment 2: The authors provide a clear evolutionary inference that OeDDES and *Panax* DDS originated from a common BAS-like ancestor, and that OeOSC12, OeOSC13, and OeOSC14 arose from tandem duplication events within *O. elatus*. OeOSC12 catalyzes the formation of dammaradienol as well as four additional products, whereas OeOSC14 appears to be highly specific for dammaradienol production. In contrast, the ancestral homologs (EsχAS, PgχAS, and SbχAS) do not produce dammaradienol, suggesting that OeOSC12 represents a key neofunctionalized enzyme in the evolution of OeDDES activity. The authors may need to include OeOSC12, EsχAS, and PgχAS in the alignment in Figure 6A, as they are closer to OeOSC14. Depending on the similarity, those three enzymes may also require function verification in Figure 6B or docking analysis.
Response 2: We thank the reviewer for this helpful suggestion. The primary purpose of Fig. 6A was to identify candidate residues that distinguish dammaradienol synthase from other OSC subfamilies. For this reason, we mainly selected functionally more specialized enzymes for the initial alignment, because inclusion of too many multifunctional OSCs could complicate the identification of residues associated with the shift between these catalytic types. To address the reviewer's concern more directly, we have now performed an additional multiple-sequence comparison including OeOSC12, OeOSC14, SbχAS, EsχAS, PgχAS, as well as the dammaradienol synthases from *Artemisia argyi* an oat OSC that can be converted to a dammaradienol-producing enzyme by amino acid substitution, in order to compare the conservation of the residue at position 259/260. These updated alignment results are now provided in Supplementary Fig. 26.
In addition, we have expanded the functional analyses in the revised manuscript. Specifically, we performed reciprocal Y259/260N mutagenesis in OeOSC12, PgχAS, and SbχAS, and found that these substitutions enabled or enhanced dammaradienol production. We also added corresponding docking analyses for OeOSC12, PgχAS, EsχAS, SbχAS, and their mutants in the revised manuscript (lines 399–407; Fig. 5D and Supplementary Fig. 24). Together, these new results strengthen our conclusion that residue 259/260 is a key determinant of product specificity, while also indicating that additional residues contribute to the emergence of full dammaradienol synthase activity.
Comment 3: Please clarify the extent of loss in the CYP716A47 homolog (OeCYP716: 7633.2 kb - 7633.8 kb) in *O. elatus* and confirm if the remnant sequence encodes a functional protein. If so, the authors may need to test its activity. The authors should consider whether there is another CYP716A47 homolog in the second subgenome of *O. elatus*. If so, clarify whether it also has undergone pseudogenization.
Response 3: We thank the reviewer for this important and constructive comment. In response, we re-examined this genomic region and the corresponding residual sequences in greater detail. Our updated analysis shows that, in both homologous *O. elatus* chromosomes (Chr11 and Chr12), CYP716A47-homologous remnants can be identified within the syntenic regions corresponding to the *Aralia elata* AeCYP716A357 locus. However, neither region contains an intact full-length CYP716A47-like gene. Instead, only short truncated CYP-like remnants are retained, preserving

approximately 350 bp and 130 bp, respectively, from the 3′ end of the coding region, compared with the ~1.5-kb full-length CDS of functional CYP716A47 homologs. Because these remnant sequences do not contain a complete open reading frame, we conclude that they are unlikely to encode functional proteins and therefore were not pursued for heterologous activity assays.

We have now revised the manuscript to clarify that both CYP716A47-corresponding loci in the two *O. elatus* subgenomes have undergone pseudogenization or partial deletion, rather than being completely absent. These revisions are reflected in the updated Results text (lines 471–479; Fig. 6E and Supplementary Fig. 32-33).

Comment 4: Although there is no direct evidence, the authors may discuss why *O. elatus* lost PK ginsenosides. Possible reasons include metabolic balance shunts or lack of essentiality for this species. The physiological role of RK ginsenosides in Araliaceae should also be addressed.

Response 4: We thank the reviewer for the constructive suggestion. As noted by the reviewer, we have updated the Discussion section accordingly (lines 522–539). Specifically, we have discussed the possible reasons for the loss of RK-type ginsenosides in *Oplopanax elatus* as well as the physiological roles of RK-type ginsenosides in Araliaceae plants.

Comment 5: Although PgχAS shares high sequence similarity with OeOSC12, it cannot catalyze dammaradienol formation. The authors may need to explain why *P. ginseng* cannot synthesize RK ginsenosides endogenously.

Response 5: We thank the reviewer for this insightful comment. We agree that this point deserves clarification. In the revised manuscript, we now explain more explicitly that, although PgχAS shares relatively high sequence similarity with OeOSC12, it does not produce detectable dammaradienol in its wild-type form and therefore cannot provide the required precursor for endogenous RK-type ginsenoside biosynthesis in *P. ginseng*.

Our mutagenesis and docking analyses suggest that this difference is at least partly related to residue 259/260. In PgχAS, the native Y259 is associated with a binding mode that favors continuation of the cyclization cascade toward non-dammaradienol products, whereas introduction of the reciprocal substitution ($Pg\chi AS^{Y259N}$) enables acquisition of dammaradienol-producing activity, although at a relatively low level. These results indicate that the native *P. ginseng* enzyme is not optimized for dammaradienol formation.

Accordingly, we interpret the absence of endogenous RK-type ginsenoside biosynthesis in *P. ginseng* mainly as a consequence of the lack of a native dammaradienol synthase step, even though *P. ginseng* possesses downstream tailoring enzymes for dammarane-type triterpenes. Instead, *P. ginseng* naturally channels flux toward dammarenediol II-derived ginsenosides, whereas RK-type ginsenosides are typically generated only during post-harvest heat processing through non-enzymatic dehydration of pre-existing major ginsenosides. These updated results are now provided in Fig. 6A.

Minor

Comment 7: Supplemental Figure 23 is missing.

Response 7: We thank the reviewer for pointing this out. We have rechecked and revised the citations of all supplementary figures throughout the manuscript.

Comment 8: The retention time described in lines 253-256 seems slightly different from that shown

in Figure 4B.

Response 8: We thank the reviewer for pointing this out. We have carefully re-examined the retention times in Figure 3B (originally Figure 4B) and have revised the updated retention times in lines 269–272.

Comment 9: Figure 2A: Please add the scale bars

Response 9: We thank the reviewer for pointing this out. We have added a scale bar to the figure and updated the figure label to Figure 1B (formerly Figure 2A).

Comment 10: Line 484, 579, 652, 659, legend of Figure 3B, 7, 8A: italicized species names

Response 10: We thank the reviewer for pointing this out. We have revised the font of the species Latin names in the indicated locations to italics. Please see Line 576, 672, 745, 752, and the legend of Figures 2B, 6 and 7A for details.

Comment 11: Line 555, 572-578, 620, 625, 684: Add space between numbers and units, and outside parenthesis

Response 11: We thank the reviewer for pointing these out. We have revised the identified errors by adding spaces between numbers and units, as well as outside parentheses, in Lines 648, 665-671, 713, 718, and 776.

Comment 12: Line 671: keep capitalization of the title consistent with that of the others

Response 12: We thank the reviewer for pointing this out. We have revised the error to ensure consistency in the subheading format. Please see Line 764 for details.

Reviewer #3 (Remarks to the Author):

Comment 1: A previously published study reporting the presence of dammarandiol acetate in Oplopanax horridus should be properly cited.

Response 1: We thank the reviewer for pointing this out. We have now cited the previously published study reporting dammaradienol acetate in *Oplopanax horridus* in the Introduction. In the revised manuscript, we also note that this finding, together with the accumulation of dammaradienol in *O. elatus*, suggests that dammaradienol and its derivatives may occur more broadly in Araliaceae plants than previously recognized (Please see line 115-117).

Comment 2: Although a high level of dammarandiol is detected in the leaves and petioles of *Oplopanax elatus*, no dammarandiol-derived saponins have been reported to date. Further discussion is required to explain why saponins derived from dammarandiol have not been identified in this species.

Response 2: We thank the reviewer for this insightful comment. We agree that this point deserves further discussion. In the revised manuscript, we now discuss that, although dammaradienol accumulates to high levels in the leaves and petioles of *Oplopanax elatus*, no dammaradienol-derived saponins have been identified to date most likely because the downstream pathway is incomplete in this species.

Specifically, our results indicate that *O. elatus* lacks a functional C12 hydroxylase equivalent to PgCYP716A47, as both corresponding loci in the two subgenomes are disrupted, and no hydroxylated intermediates such as 12-hydroxydammaradienol or 6,12-dihydroxydammaradienol were detected. In addition, the downstream UGT genes we identified are predominantly expressed in roots, whereas *OeOSC14* is mainly expressed in leaves, suggesting a likely spatiotemporal mismatch between upstream and downstream pathway genes. Together, these observations suggest

that dammaradienol is produced efficiently but is not further converted into hydroxylated or glycosylated saponins. We have added this interpretation to the revised Discussion (lines 522–527).

Comment 3: It would be worthwhile to consider the possibility that, similar to *Oplopanax horridus*, dammarandiol in *Oplopanax elatus* may be converted into dammarandiol acetate and stored primarily in this acetylated form rather than as saponins. Additional speculation on the potential natural roles of these compounds would strengthen the discussion.

Response 3: We thank the reviewer for this thoughtful suggestion. We agree that this is an important possibility to consider. In the revised Discussion, we now note that, similar to the report in *Oplopanax horridus*, dammaradienol in *O. elatus* may potentially undergo further modification such as acetylation and be stored in a less polar form rather than being converted into glycosylated saponins. At present, however, we have not directly detected dammaradienol acetate in *O. elatus*, and we therefore present this as a plausible hypothesis rather than a demonstrated pathway.

We have also expanded the Discussion to note that the natural roles of dammaradienol and its possible derivatives in Araliaceae remain unclear. Given its high accumulation in leaves and petioles, these compounds may have ecological or physiological functions, for example in defense or stress adaptation, but this will require further experimental investigation. These points have now been added to the revised Discussion (lines 522–534).

Comment 4: A dammaradienol synthase, AarOSC20433, was identified for the first time in *Artemisia argyi*. This study should be cited, and a comparative analysis of the amino acid sequence of this enzyme with OeOSC14 would be informative.

Response 4: We thank the reviewer for this valuable suggestion. We have now cited the study reporting AarOSC20433 as the first functionally characterized dammaradienol synthase from *Artemisia argyi* in the Introduction. In addition, as suggested, we carried out a comparative amino acid sequence analysis between AarOSC20433 and OeOSC14, together with OeOSC12 and other related OSCs. These results are now included in Supplementary Fig. 26, and the relevant discussion has been added to the revised manuscript (Please see line 410-416).

Comment 5: The Introduction should include information on the major triterpenes and saponins that have been reported in *Oplopanax elatus*.

Response 5: We thank the reviewer for this helpful suggestion. We have now revised the Introduction to include the major triterpene and saponin types reported in *O. elatus*, including representative compounds such as cirenshenosides S, T, U, and V (Please see line 539-542)**.**

Comment 6: At line 77, ginsenoside Rk1 is described as having the strongest activity. Additional explanation and supporting evidence are required, particularly citing Reference 11, which reports the biological activity of ginsenoside Rk1.

Response 6: We thank the reviewer for pointing this out. We agree that the original statement required clearer explanation and stronger supporting evidence. In response, we have revised this part of the Introduction to provide additional explanation of the reported biological activity of ginsenoside Rk1 and have added the appropriate supporting citation, including Reference 11 (Please see line 76-78).

Comment 7: At line 111, the phrase "dammaradienol in its leaves but not RK-type ginsenosides" should be revised to "dammaradienol in its leaves but not hydroxylated dammaradienol or glycosylated sapogenins (RK-type ginsenosides)" for greater clarity and accuracy.

Response 7: We thank the reviewer for this helpful suggestion. We agree that the original phrasing could be more precise. In response, we have revised the sentence to read "dammaradienol in its leaves but not hydroxylated dammaradienol or glycosylated sapogenins (RK-type ginsenosides)" for greater clarity and accuracy (Please see line 117-120).

Comment 8: In Figure 5, the order of the legends for panels C and D appears to be incorrect and should be reversed.

Response 8: We thank the reviewer for pointing this out. We have corrected the order of the panel legends for Fig. 5C and Fig. 5D in the revised figure. In the updated manuscript, this figure is now presented as Fig. 4.

Comment 9: To date, no studies have reported genes responsible for the biosynthesis of ocotillol-type triterpenes. In Figure 5C, it is unclear whether the DDS genes belonging to clade III are proposed to produce ocotillol-type triterpenes. The authors should clarify whether this is supported by experimental evidence or is merely speculative.

Response 9: We thank the reviewer for this important comment. We agree that the original wording in this section could be interpreted too broadly and required clarification.

First, we would like to clarify that genes responsible for the biosynthesis of ocotillol-type triterpenes have in fact been reported, including in the studies "Comparative genomics reveals the diversification of triterpenoid biosynthesis and origin of ocotillol-type triterpenes in *Panax*" and "Promiscuous oxidosqualene cyclases from *Neoalsomitra integrifoliola* catalyzing the formation of tetracyclic, pentacyclic, and heterocyclic triterpenes." We have now cited these studies in the revised manuscript to more accurately reflect the current state of knowledge.

Second, in our manuscript, we do not intend to propose that all *DDS* genes in the clade III lineage are dedicated ocotillol-type triterpene synthases. Rather, our point was that experimentally characterized *Panax* DDS enzymes primarily produce dammarenediol II, while some reports have shown that they can also generate low-abundance side products, including ocotillol-type triterpenes. We agree that this distinction was not sufficiently clear in the original text.

We have therefore revised the corresponding Results text to state more explicitly that the clade III genes are interpreted as DDS-like enzymes primarily associated with dammarenediol II production, and that the mention of ocotillol-type triterpenes refers only to minor byproducts reported for some experimentally characterized enzymes, rather than to a dedicated or generalized function of the entire clade. We believe this revision avoids overinterpretation and better separates experimental evidence from evolutionary inference. The corresponding revisions can be found in lines 300–302.

Comment 10: The authors should discuss whether overexpression of OeOSC14 in ginseng plants could potentially enhance the production of RK-type ginsenosides (Rk1, Rk2, and Rk3).

Response 10: We thank the reviewer for this valuable suggestion. We agree that overexpression of OeOSC14 in *Panax* could be a promising strategy to enhance production of RK-type ginsenosides (Rk1, Rk2, and Rk3) by supplying the dammaradienol precursor in a host that already contains the downstream tailoring machinery. We have added this point to the revised Discussion. At the same time, we note that this remains speculative and will require experimental validation.

Comment 11: In Figure 6D, the term "Deprotonation" alone is insufficient. The description would be clearer if revised to "Deprotonation-mediated double bond formation."

Response 11: We thank the reviewer for pointing this out. We have revised the description of this sentence, and the updated figure is shown in Figure S27.

Comment 12: *Oplopanax elatus* is likely to possess multiple cytochrome P450 (CYP) genes involved in the conversion of triterpenes into saponins. Although this is not the primary focus of the present study, discussion of potential biosynthetic pathways and synthetic circuits for reported saponins such as cirenshenosides S, T, U, and V would add value to the manuscript.

Response 12: We thank the reviewer for this valuable suggestion. We agree that, although this aspect is beyond the main focus of the present study, discussion of the broader triterpene saponin biosynthetic capacity of *Oplopanax elatus* would strengthen the manuscript.

In the revised manuscript, we now note that we have functionally characterized three β-amyrin synthases (OeOSC6, OeOSC8, and OeOSC16) and two lupeol synthases (OeOSC10 and OeOSC15) from *O. elatus*, which likely provide the triterpene scaffolds for cirenshenoside-type saponins. We further point out that the *O. elatus* genome contains numerous CYP genes, although the enzymes responsible for scaffold modification leading to reported saponins such as cirenshenosides S, T, U, and V have not yet been functionally characterized. We therefore added a brief perspective to the revised Discussion highlighting that the *O. elatus* genome provides an important foundation for future identification of the relevant CYPs and UGTs involved in scaffold decoration and glycosyl chain assembly. The corresponding revision can be found in lines 539-542.

Comment 13: The new results showing that co-expression of OeOSC14 with two ginseng-derived CYP genes responsible for protopanaxadiol and protopanaxatriol biosynthesis enables the production of 12-hydroxydammaradienol and 6,12-dihydroxydammaradienol in tobacco are particularly interesting. Furthermore, the functional characterization of *Oplopanax elatus*–derived UGTs (OeUGT74AE2, OeUGT94Q2, and OeUGT71A2) and the successful reconstruction of a biosynthetic pathway leading to the production of RK-type ginsenosides (Rk1, Rk2, and Rk3) represent a significant achievement. These results strongly suggest the feasibility of synthesizing RK-type ginsenosides in ginseng and other plant systems in the future.

Response 13: We thank the reviewer for this positive comment. We have incorporated this point into the revised Discussion (line 527-531).

Comment 14: Finally, the reference list requires careful revision, as the use of uppercase and lowercase letters in the titles is inconsistent and does not follow a uniform formatting rule.

Response 14: We thank the reviewer for pointing this out. In the revised manuscript, we have carefully corrected the formatting of the references.

Reviewer #4 (Remarks to the Author):

In this study, leveraging genome sequencing of *Oplopanax elatus*, the authors identified OeOSC14, an oxidosqualene cyclase responsible for the biosynthesis of dammaradienol, the precursor of RK-type ginsenosides. They then investigated the evolutionary origin of OeOSC14 through phylogenetic and comparative genomic analyses. Furthermore, multiple sequence alignment, site-directed mutagenesis, and QM/MM multiscale simulations were employed to identify a key amino acid residue, N260, that governs the dammaradienol synthase specificity of OeOSC14. Finally, by combining PgCYP716A47 and PgCYP716A53v2 from *Panax ginseng* with three newly identified glycosyltransferases from *O. elatus*, the authors successfully reconstituted the biosynthetic pathway of RK-type ginsenosides in *Nicotiana benthamiana*. Nevertheless, several issues and questions regarding this study remain:

Comment 1: A dammaradienol synthase, AarOSC20433, has been identified from *Artemisia argyi* (ACS Synth. Biol. 2024, 13, 12, 4015–4026). The authors should cite this enzyme and discuss it appropriately in the manuscript.

Response 1: We thank the reviewer for this valuable suggestion. We have now cited the study reporting AarOSC20433 as a dammaradienol synthase from *Artemisia argyi* and added the relevant discussion in the revised manuscript. We also included a comparative analysis between AarOSC20433 and OeOSC14, which helps further contextualize the evolutionary and functional features of OeOSC14 relative to the only other reported plant dammaradienol synthase. The corresponding revisions can be found in lines 106–108, and 410-412.

Comment 2: The results shown in Figure 3B do not correspond to the descriptions provided in the manuscript.

Response 2: We thank the reviewer for this careful observation. We have revised the relevant text in the Results section so that it now accurately reflects the data presented. In addition, the original Fig. 3B has been reorganized and is now presented as Fig. 2C in the revised manuscript. The corresponding revisions can be found in lines 224–226.

Comment 3: Lines 241–245. These descriptions are confusing. The data presented in Figure 4A and Supplementary Figure 20 do not support these statements. It is unclear how the authors conclude that 15 triterpene-related OeOSC genes are predominantly expressed in leaves based on these data. Moreover, the eight candidates selected for functional characterization do not appear to meet the stated selection criterion, namely the exclusion of lowly expressed genes (FPKM < 1).

Response 3: We thank the reviewer for pointing this out. We agree that the original wording in lines 241–245 was confusing and did not accurately reflect the data shown in Fig. 4A and Supplementary Fig. 20. We have therefore revised this part of the Results section substantially. In the revised manuscript, we no longer describe the 15 triterpene-related OeOSC genes as being predominantly expressed in leaves. Instead, we now state that they show distinct tissue-specific expression patterns, with five candidates (OeOSC10, OeOSC12, OeOSC13, OeOSC14, and OeOSC16) predominantly expressed in leaves and the remaining selected candidates mainly expressed in roots and seeds. We also corrected the candidate-selection description by explicitly stating that six lowly expressed genes (FPKM < 1) were excluded and that nine candidates were selected for functional characterization (Please see line 252-259). We believe these revisions have substantially improved the clarity and accuracy of this section.

Comment 4: Based on Figure 4B, transient expression of OeOSC12 produced five, rather than four, additional peaks compared with the control, and transient expression of OeOSC14 produced two additional peaks rather than one. What are the identities of these peaks?

Response 4: We thank the reviewer for pointing this out. We agree that the original text did not accurately describe all of the additional peaks observed in the GC–MS chromatograms. In response, we have revised this section to explicitly distinguish between peaks that could be assigned by comparison with authentic standards and those that remain uncharacterized.

In the revised manuscript, we now clarify that transient expression of OeOSC12 produced five additional peaks compared with the control, corresponding to dammaradienol, β-amyrin, α-amyrin, Dammarenediol II, and one unidentified compound. Likewise, transient expression of OeOSC14

produced four additional peaks compared with the control, corresponding to dammaradienol and three unidentified compounds. Because these four unknown products accumulated at levels too low for structural elucidation by NMR, we have marked them with asterisks in Fig. 3B (previously Fig. 4B) and have provided their mass spectra in Supplementary Fig. 18. These revisions improve the accuracy and completeness of the product description in the Results section.

Comment 5: In the left panel of Figure 5A and in Supplementary Fig. 22, OeDDES should be highlighted using a distinct color or symbol to facilitate its identification. From an evolutionary perspective, plant triterpene synthases are thought to have originated from cycloartenol synthase (CAS). This view is consistent with the phylogenetic tree shown in Figure 4A, but appears to be inconsistent with the phylogenetic trees presented in Figure 5A (left) and Supplementary Fig. 22. How should these apparently conflicting results be interpreted?

Response 5: We thank the reviewer for these two important comments.

First, regarding the visual presentation of OeDDES, we agree that this enzyme should be more clearly identifiable in the phylogenetic trees. In the revised figures, we have therefore highlighted OeDDES using a distinct color (red) in the left panel of Fig. 4A and in Supplementary Fig. 22. We also highlighted the closely related OeOSC12 to facilitate comparison within this lineage.

Second, regarding the apparent inconsistency among the phylogenetic trees, we agree that this issue required clarification. The generally accepted evolutionary framework is that cycloartenol synthase (CAS) represents the ancestral form of plant OSCs, from which other OSC subfamilies, including LAS, LUS, and BAS, diversified through gene duplication and neofunctionalization. We recognized that the topology shown in the original version of Fig. 5A and Supplementary Fig. 22 was not fully consistent with this framework. To address this issue, we reconstructed the OSC phylogeny using the revised sequence set and alignment, and replaced the corresponding trees in the revised manuscript. The updated phylogenetic trees are now consistent with the established evolutionary trajectory of plant OSCs and provide a more coherent framework for interpreting the origin of OeDDES.

We believe these revisions have improved both the readability of the figures and the consistency of the evolutionary interpretation.

Comment 6: Supplementary Figure 23 is not shown in the Supplementary Information.

Response 6: We thank the reviewer for pointing this out. We have rechecked and revised all supplementary figures throughout the manuscript.

Comment 7: The data presented are not sufficient to support the conclusion that “dammaradienol synthase emerged from an ancestral β-amyrin synthase through gene duplication, neofunctionalization, and subsequent functional specialization.” Based on the phylogenetic tree shown in Figure 5A and the synteny analysis in Figure 5C (note that the panel is labeled C in the figure but D in the legend), an alternative interpretation is that OeDDES may have arisen from an mTTS via gene duplication, neofunctionalization, and subsequent functional specialization. Additional synteny analyses are necessary to substantiate a proposed evolutionary origin of mTTS from BAS.

Response 7: We thank the reviewer for this important comment and agree that our original wording was too strong. We have therefore revised the manuscript to state more cautiously that OeDDES most likely arose from an mTTS-like ancestor through gene duplication, neofunctionalization, and subsequent functional specialization, rather than directly from a canonical BAS. In this framework, the mTTS lineage is interpreted as having originated more indirectly from a BAS-like ancestor. This

revised interpretation is better supported by both the phylogenetic topology and the synteny analysis: the grape BAS locus shows clear syntenic relationships with mTTS-associated loci, but not a direct one-to-one syntenic relationship with OeDDES, while the functional properties of OeOSC12 and the reciprocal Y259/260N mutagenesis results indicate that dammaradienol synthase activity emerged on the basis of an existing mTTS scaffold. We have revised the relevant text and updated the corresponding figure interpretation in Fig. 4C (formerly Fig. 5C), and we also corrected the panel-label inconsistency noted by the reviewer (lines 296–339 and 546-548).

Comment 8: The syntenic blocks containing mTTS are well conserved among the eight Araliaceae species (Figure 5C). However, although no mTTS is present in the corresponding syntenic block of *E. senticosus*, putative mTTSs (EsχAS) that may have arisen through translocation events were identified at other chromosomal locations. By the same logic, the absence of a full-length CYP716A gene in the corresponding syntenic region of *O. elatus* does not necessarily indicate the loss of a C12 hydroxylase. Have the authors considered functionally characterizing Oela19G01405.1, which clusters in the same clade as PgCYP716A47 in the phylogenetic tree?

In addition, the failure to detect 12-hydroxydammaradienol, 6,12-dihydroxydammaradienol, or RK-type ginsenosides in different tissues of *O. elatus* may also result from spatiotemporal mismatches between the expression of the upstream gene OeOSC14 and downstream pathway genes. For example, the newly identified glycosyltransferases OeUGT94Q2, OeUGT74AE2, and OeUGT71A2 exhibit predominant expression in roots rather than leaves.

Response 8: We thank the reviewer for this insightful and constructive comment. We agree that the absence of a full-length CYP716A gene in the syntenic region alone does not exclude the possibility that a functionally related homolog may have been retained elsewhere in the genome, analogous to the case of EsχAS, which may have arisen through translocation. We have therefore clarified this point in the revised manuscript.

To address this possibility directly, we further examined the CYP716A family in the *O. elatus* genome. Our phylogenetic analysis did not identify any intact gene that could be confidently assigned as a functional C12 hydroxylase equivalent to PgCYP716A47. We also functionally characterized Oela19G01405.1, which clusters with PgCYP716A47 in the phylogenetic tree. Our results show that Oela19G01405.1 is not a C12 hydroxylase; instead, it exhibits the same activity as AeCYP716A355 and PgCYP716A141, catalyzing C16 hydroxylation of pentacyclic triterpenes. These data are now included in Supplementary Fig. 31, and the corresponding description has been added to the revised manuscript (lines 460–465). Thus, although we cannot exclude genome rearrangement in principle, our current functional evidence does not support the presence of an alternative C12 hydroxylase retained elsewhere in the *O. elatus* genome.

We also agree that spatiotemporal mismatch between upstream and downstream pathway genes likely contributes to the absence of downstream products. In particular, OeUGT94Q2, OeUGT74AE2, and OeUGT71A2 are predominantly expressed in roots, whereas OeOSC14 is mainly expressed in leaves, which may limit further glycosylation of dammaradienol. At the same time, we still consider loss of C12 hydroxylase to be the key factor, because: (1) we did not detect hydroxylated intermediates (12-hydroxydammaradienol, 6,12-dihydroxydammaradienol, PPD, or PPT) or downstream RK-/DM-type ginsenosides in any examined tissue; (2) the PgCYP716A47-corresponding loci are disrupted in both *O. elatus* subgenomes; and (3) dammaradienol accumulates to high levels in leaves, whereas other triterpene scaffolds are only detected at relatively low levels. We have now clarified in the revised manuscript that the absence of downstream dammarane-

derived products in *O. elatus* is most likely caused by the combined effects of C12 hydroxylase loss and spatiotemporal mismatch of pathway gene expression (lines 527–531).

Comment 9: There are multiple instances in the manuscript with grammatical or logical issues, for example in lines 100–103, 108–111, and 142–145. For clarity, please provide the full term when an abbreviation is used for the first time (e.g., BAS).

Response 9: We thank the reviewer for pointing this out. We have revised these sentences accordingly. Please see Lines 103-105, 117-122 and 154–156 in the revised manuscript for details.

“Point-by-Point” Responses to Reviewers’ Comments:

Reviewer #1 (Remarks to the Author):
All my previous comments were fully addressed by the authors. I appreciate the authors for their efforts to revise the manuscript. This study provides important insights on how the triterpenoid saponins have diversified. I have no more comments.

Reviewer #2 (Remarks to the Author):
The authors have addressed all comments, and I have no further suggestions.

Reviewer #3 (Remarks to the Author):
Overall, all questions and revision requirements raised by me have been addressed, and the responses are considered appropriate.
However, there are still a few points to be addressed; therefore, further revisions are required as follows

Comment 1. Regarding 'dammarenediol-II,' the usage of the hyphen is inconsistent. I recommend unifying it to a single format throughout the manuscript.
Response 1：We thank the reviewer for pointing out this inconsistency. We have now unified the format to dammarenediol II (without hyphen) throughout the entire manuscript, including the main text, figures, tables, and supplementary materials.

Comment 2. An Rtx-5MS capillary column (30m× 0.25mm×0.25 μm, Shimadzu) was used for the isolation of triterpene.
The term 'separation' seems more suitable than 'isolation' for the process described in this sentence
Response 2：We agree with the reviewer that “separation” is more appropriate in this context. The term has been revised accordingly in the manuscript (see lines 682).

Comment 3. I noticed that some journal names in the references are written in full, while others use abbreviations. I suggest standardizing them all to the appropriate abbreviations. Please review the references thoroughly for consistency.
In several references, full journal names are still used.

European Food Research and Technology
Journal of the American Chemical Society.
Plant Biotechnology Journal
ACS Synthetic Biology.
Chinese Herbal Medicines.
Pharmaceutical Chemistry Journal.

Nucleic Acids Research.

The references listed below are incomplete. Please provide the missing details.

34. Telomere-to-telomere reference genome for Panax ginseng highlights the evolution of saponin biosynthesis. (no further information)

66. Eddy, S. R. Accelerated Profile HMM Searches. PLoS Comput Biol. 7, e1002195 (2011).

Accelerated profile HMM Searches

Response 3： We thank the reviewer for carefully checking the reference list. We have now standardized all journal names to their official abbreviations throughout the manuscript. In addition, we have completed the incomplete references. The entire reference list has been thoroughly revised for consistency and completeness. We apologize for these oversights.

Reviewer #4 (Remarks to the Author):

Comment 1. The manuscript would benefit from improved conciseness, as certain sections contain redundant or overly detailed descriptions. Some of this information could be more appropriately placed in the Methods section or supplementary tables, rather than being extensively described in the main text—for instance, lines 184–188.

Response 1： We thank the reviewer for this constructive suggestion. We agree that some parts of the original manuscript, including the section in lines 184–188, contained overly detailed technical descriptions that were more appropriate for the Methods section or Supplementary Tables. We have therefore revised the manuscript to improve conciseness and readability. Specifically, we shortened the genome assembly and annotation section by retaining only the key information necessary to evaluate the quality of the *O. elatus* genome assembly, including chromosome-level anchoring, assembly size, contig N50 and BUSCO completeness. Detailed sequencing depth, genome size estimation, Hi-C anchoring procedures, contig statistics, repeat classification and annotation metrics are now provided in the Methods section and Supplementary Tables 1–9.

Comment 2. The explanation of the mechanistic role of the key residue Asn (N260) in Lines 387–416, together with the results presented in Fig. 5D, is not sufficiently clear and is difficult to follow. Specifically, illustrating either the distance between the hydrogen atom on the side-chain amide of Asn (N260) and the carbon atoms of the product dammaradienol, or the distance between the phenolic hydroxyl hydrogen of Tyr (Y260/259) and the carbon atoms of the phenyl ring of Phe (F413/412), does not adequately explain how residue N260 influences product structure formation.

Response 2：We thank the reviewer for this helpful comment. We agree that our previous explanation did not clearly distinguish between simple distance measurements and their mechanistic interpretation. We have therefore revised the corresponding section to clarify that these distances are not intended to demonstrate a direct catalytic contact, but rather to describe changes in the local active-pocket environment associated with residue 260.

In the revised text, we show that the N260Y substitution alters the modeled orientation of dammaradienol within the catalytic pocket. In wild-type OeOSC14, dammaradienol is surrounded within 5 Å by N260, F474, F729, L735 and Y737, with N260 positioned near C10 and F474 near C20 of dammaradienol. By contrast, in OeOSC14N260Y, the ligand-proximal environment is reorganized, F413 is recruited into the 5 Å range of docked dammaradienol, and Y260 is positioned

close to F413. We also added electrostatic surface potential analysis, which shows altered charge distribution and surface properties in the OeOSC14^{N260Y} pocket compared with wild-type OeOSC14.

Together, these revisions support a clearer model in which residue 260 influences product specificity not through a direct catalytic role in double-bond formation, but by reshaping the local pocket configuration, electrostatic environment and ligand-binding orientation. The revised text has been updated in the main manuscript (Line 386–419), and the new electrostatic analysis has been added as Supplementary Fig. 25.

Comment 3. Based on the description in Lines 503–505 and the data presented in Supplementary Figs. 36–37, it is not clear how OeUGT71A2 was identified.

Response 3： We thank the reviewer for highlighting this important point. We apologize for the lack of clarity regarding how OeUGT71A2 was identified. Our aim was to identify UGTs capable of catalyzing C6-OH glycosylation of 6,12-dihydroxydammaradienol. Therefore, UGTPg101 from *P. ginseng*, which catalyzes the transfer of the first sugar unit to the C6-OH of protopanaxatriol, was used as an important reference gene for candidate screening.

Candidate UGT genes were selected by integrating synteny and phylogenetic analyses. The synteny analysis identified only OeUGT71A1 as a putative ortholog of UGTPg101. However, phylogenetic analysis showed that OeUGT71A2 clustered more closely with UGTPg101 than OeUGT71A1 did. Consistently, sequence comparison showed that OeUGT71A2 shared higher amino acid sequence similarity with UGTPg101 than OeUGT71A1, with similarities of 88.5% and 77.0%, respectively.

Therefore, although OeUGT71A2 was not identified through synteny, its closer phylogenetic relationship and higher sequence similarity to UGTPg101 supported its inclusion as an additional candidate for functional validation. Functional assays further confirmed that OeUGT71A2 has activity toward the corresponding substrate. To make this clearer, we have added the reference gene names to the main text for candidate screening (Lines 502–506) and revised Supplementary Fig. 37 by highlighting OeUGT71A2 and the other candidate UGT genes, thereby clarifying the rationale for including OeUGT71A2 in the functional analysis.

Comment 4. Transient expression of OeOSC14 together with tHMGR in tobacco leaves yields nearly 4 mg/g DW (Fig. 5c). However, upon introduction of downstream pathway genes, the highest reported yield of the final products RK1/2/3 reaches only 23.07 μg/g DW. This substantial decrease suggests that the downstream hydroxylases and/or glycosyltransferases may have relatively low catalytic efficiency under the tested conditions, potentially limiting their suitability for efficient production of RK-type triterpenoid saponins.

Response 4：We thank the reviewer for this insightful observation. Indeed, the sharp decrease from ~3.63 mg/g DW of dammaradienol to ~23 μg/g DW of Rk3, with even lower yields of Rk1 and Rk2, suggests that downstream hydroxylases and glycosyltransferases are likely rate-limiting under the tested conditions. We have added a discussion of this limitation in the revised manuscript (Lines 538–556), noting that the current *N. benthamiana* system should be regarded as a proof-of-concept platform rather than an optimized production system. We also suggest future strategies to improve RK-type saponin production, including screening or engineering more efficient CYPs and UGTs, balancing pathway enzyme expression, enhancing metabolic flux toward hydroxylated and glycosylated intermediates, and exploring more compatible chassis systems.
