# Supplementary material for: The Oplopanax elatus genome reveals dammaradienol synthase evolution enabling reconstruction of RK type ginsenosides biosynthesis
Source: Nat Commun. 2026 Jun 24;17:7907. doi: 10.1038/s41467-026-74686-6 (PMC13444123; doi:10.1038/s41467-026-74686-6)
Supplement: Supplementary file 4 [file 41467_2026_74686_MOESM3_ESM.pdf]

Description of Additional Supplementary Files

File Name: Supplementary Data 1
Description: NMR data for dammaradienol

File Name: Supplementary Data 2
Description: Annotation of the 19 oxidosqualene cyclase (OSC) genes in Oplopanax elatus

File Name: Supplementary Data 3
Description: Coding gene and translated protein sequences of genes with functionally characterized in this study.

File Name: Supplementary Data 4
Description: The GeneBank ID of OSC proteins used for phylogenetic tree construction in this study.

File Name: Supplementary Data 5
Description: The GeneBank ID of CYP proteins used for phylogenetic tree construction in this study.

File Name: Supplementary Data 6
Description: The GeneBank ID of UGT proteins used for phylogenetic tree construction in this study.

File Name: Supplementary Data 7
Description: Plasmids used in this study.

File Name: Supplementary Data 8
Description: Primers used in this study.

File Name: Supplementary Data 9
Description: The summary of detection of triterpene saponins in this study.
